# Quantitative Single-Cell Mass Spectrometry Provides a Highly Resolved Analysis of Natural Product Biosynthesis Partitioning in Plants

**DOI:** 10.1021/jacs.4c06336

**Published:** 2024-08-14

**Authors:** Anh Hai Vu, Moonyoung Kang, Jens Wurlitzer, Sarah Heinicke, Chenxin Li, Joshua C. Wood, Veit Grabe, C. Robin Buell, Lorenzo Caputi, Sarah E. O’Connor

**Affiliations:** † Department of Natural Product Biosynthesis, Max Planck Institute for Chemical Ecology, Jena 07745, Germany; ‡ Center for Applied Genetic Technologies, University of Georgia, Athens, Georgia 30602, United States; § Department of Crop and Soil Sciences, University of Georgia, Athens, Georgia 30602, United States; ∥ Microscopic Imaging Service, Max Planck Institute for Chemical Ecology, Jena 07745, Germany; ⊥ Institute of Plant Breeding, Genetics, and Genomics, University of Georgia, Athens, Georgia 30602, United States

## Abstract

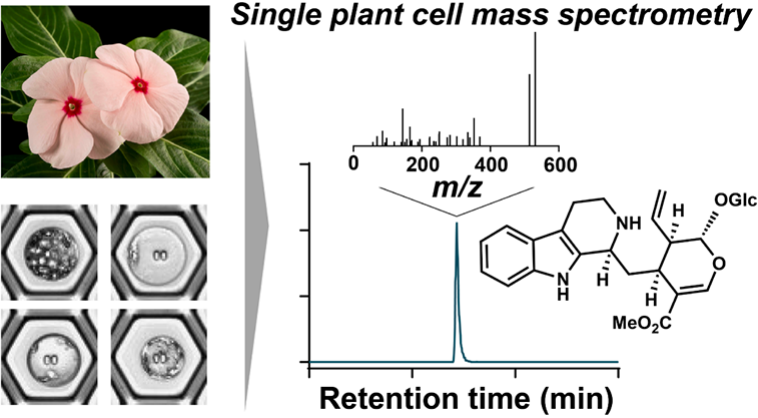

Plants produce an extraordinary array of natural products (specialized metabolites). Notably, these structurally complex molecules are not evenly distributed throughout plant tissues but are instead synthesized and stored in specific cell types. Elucidating both the biosynthesis and function of natural products would be greatly facilitated by tracking the location of these metabolites at the cell-level resolution. However, detection, identification, and quantification of metabolites in single cells, particularly from plants, have remained challenging. Here, we show that we can definitively identify and quantify the concentrations of 16 molecules from four classes of natural products in individual cells of leaf, root, and petal of the medicinal plant *Catharanthus roseus* using a plate-based single-cell mass spectrometry method. We show that identical natural products show substantially different patterns of cell-type localization in different tissues. Moreover, we show that natural products are often found in a wide range of concentrations across a population of cells, with some natural products at concentrations of over 100 mM per cell. This single-cell mass spectrometry method provides a highly resolved picture of plant natural product biosynthesis partitioning at a cell-specific resolution.

## Introduction

**Figure 1:**
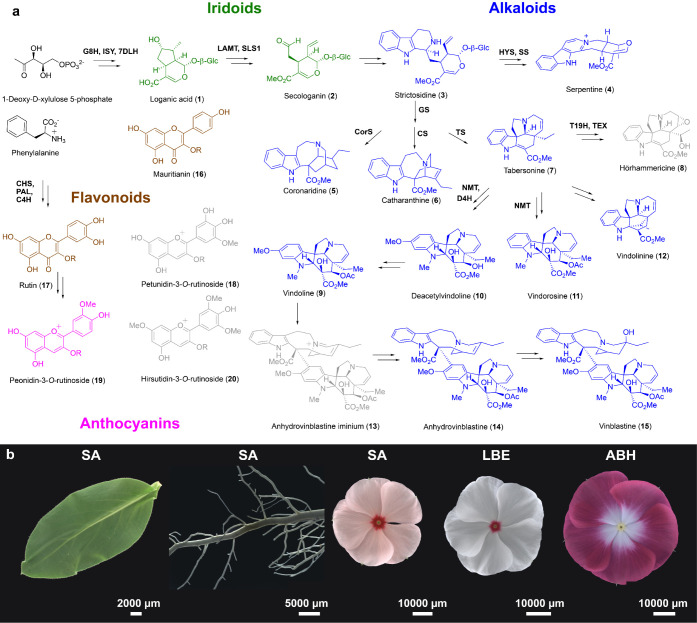
a, Simplified biosynthetic pathways of natural products produced in the medicinal plant *Catharanthus roseus*. Compounds colored green are iridoid monoterpenes, which are also precursors for monoterpene indole alkaloids, shown in blue. Flavonoids are shown in brown, and anthocyanins in pink. Compounds drawn in gray are mentioned in the main text but are not quantified with an authentic standard (see Table S1 for the definition of enzyme abbreviations). b, Photos of studied tissues (from left to right): Sunstorm Apricot (SA) leaf, SA root, SA flower, Little Bright Eyes (LBE) flower, and Atlantis Burgundy Halo (ABH) flower.

Plants synthesize valuable natural products that are widely used in the pharmaceutical, agrichemical, flavor, and fragrance industries. In plants, which are highly complex multicellular organisms, biosynthetic enzymes that construct these molecules are expressed only in a few specific types of cells.^1−3^ In one notable example, the ca. 40-enzyme biosynthetic pathway of the anticancer drug vinblastine (**15**) (Figure 1a), a natural product that is produced in *Catharanthus roseus* (Madagascar periwinkle), occurs in only three types of leaf cells:^4^ the first part of this alkaloid biosynthetic pathway is found in internal phloem-associated parenchyma (IPAP) cells, the middle module of the pathway occurs in epidermal cells, and finally, the last steps occur in idioblast/laticifer cells.

Since natural products are compartmentalized to specific cells, the biosynthetic pathways of plants can, in principle, be more rapidly elucidated using newly developed single-cell omics approaches. For example, single-cell RNA-sequencing (scRNA-seq) was recently used to show the cell-type specific expression profiles of the vinblastine (**15**) biosynthetic genes in *C. roseus* leaves.^5,6^ However, to fully understand natural product biosynthesis in plants, the corresponding products and biosynthetic intermediates must also be mapped at the single-cell level.

Unfortunately, existing single-cell mass spectrometry (scMS) methods, namely mass spectrometry imaging and live single-cell mass spectrometry, are limited by arduous sample preparation procedures and low throughput.^7−9^ Microfluidics-based methods that can rapidly sort cells have been adapted only for mammalian cells.^10^ More importantly, all of these methods lack the possibility of integrating mass spectrometry with chromatographic separation, which is required for both quantification and more definitive structural identification using retention times and fragmentation patterns in comparison to authentic standards.^11^

We recently showed that several alkaloids in individual cells derived from *C. roseus* leaf tissue could be detected by mass spectrometry.^5^ Here, we show that scMS can be broadly applied to cells derived from multiple tissues (leaf, root, and petal of plants (Figure 1b)), and can also be adapted to rigorously identify and quantify a range of metabolite classes (Figure 1a) at a throughput of approximately 180 cells/day. These data reveal that cell-specific localization patterns of alkaloid, phenylpropanoid, and monoterpene metabolite accumulation vary among organs. Moreover, these scMS data also show that metabolites accumulate at highly variable levels within cell populations, with a minority of individual plant cells having alkaloids, monoterpenes, and/or flavonoids at concentrations in excess of 100 mM. Overall, this scMS approach provides highly resolved profiles of how and where natural products are located at the cell-specific level, which, in combination with single-cell sequencing methods, provides an improved foundation for gene discovery efforts, plant metabolic engineering, and understanding the function of natural products.

## Results

### Bulk Tissue Analysis of Leaf, Root, and Petal Tissues

Before single-cell analysis, bulk tissue analyses were performed using an ultra-high-performance liquid chromatography high resolution mass spectrometry (UHPLC-HRMS) platform in the configuration used for single cells. The aim was to assess the chemical space of the plant tissues used for the single-cell experiments and validate instrument stability over three consecutive days of continuous measurements (Table S2). Principal component analysis (PCA) of the processed data showed a clear separation of the tissues, as expected (Figure S1). In these diluted bulk tissue samples, a total of 1014 features with a molecular formula assigned with less than 2 ppm error were detected (Supporting Information Data 1). This diluted sample, which yielded an MS signal comparable to what was observed in single-cell analyses (see below), was used to validate the MS method.

The identities of bulk analysis features were first predicted based on their fragmentation spectra and library searches via SIRIUS.^12−14^ The results from SIRIUS revealed the presence of 122 alkaloids, 66 flavonoids and anthocyanins, and 5 iridoids across all three tissues (Supporting Information Data 2). We were able to confirm the identity of 18 metabolites using authentic standards and quantify 16 of them using external calibration (Figures S2–S4, Tables S3 and S4, and Supporting Information MS^2^ spectra). These compounds included iridoids (loganic acid (**1**) and secologanin (**2**)), flavonoids (rutin (**17**) and mauritianin (**16**)), anthocyanin (peonidin-3-*O*-rutinoside (**19**)), and a variety of monoterpene indole alkaloids (Figures 1a and S2, Table S2). Mauritianin (**16**) is a glycosylated form of kaempferol, while rutin (**17**) is a quercetin glycoside, and the occurrence of both the kaempferol and quercetin aglycones in *C. roseus* flowers is known.^15^ The anthocyanin peonidin-3-*O*-rutinoside (**19**), for which a standard is also available, was detected in the petals. Since the authentic standards for no other detectable anthocyanins in *C. roseus* were available, we made tentative assignments based on the mass and fragmentation pattern of two additional anthocyanins, one (petunidin rutinoside-like) observed in all three cultivars, while the other (hirsutidin rutinoside-like) was exclusively present in the ABH cultivar (Figure S2). Petunidin and hirsutidin scaffolds have been previously reported in *C. roseus* petals.^16,17^

### The ScMS Workflow

**Figure 2:**
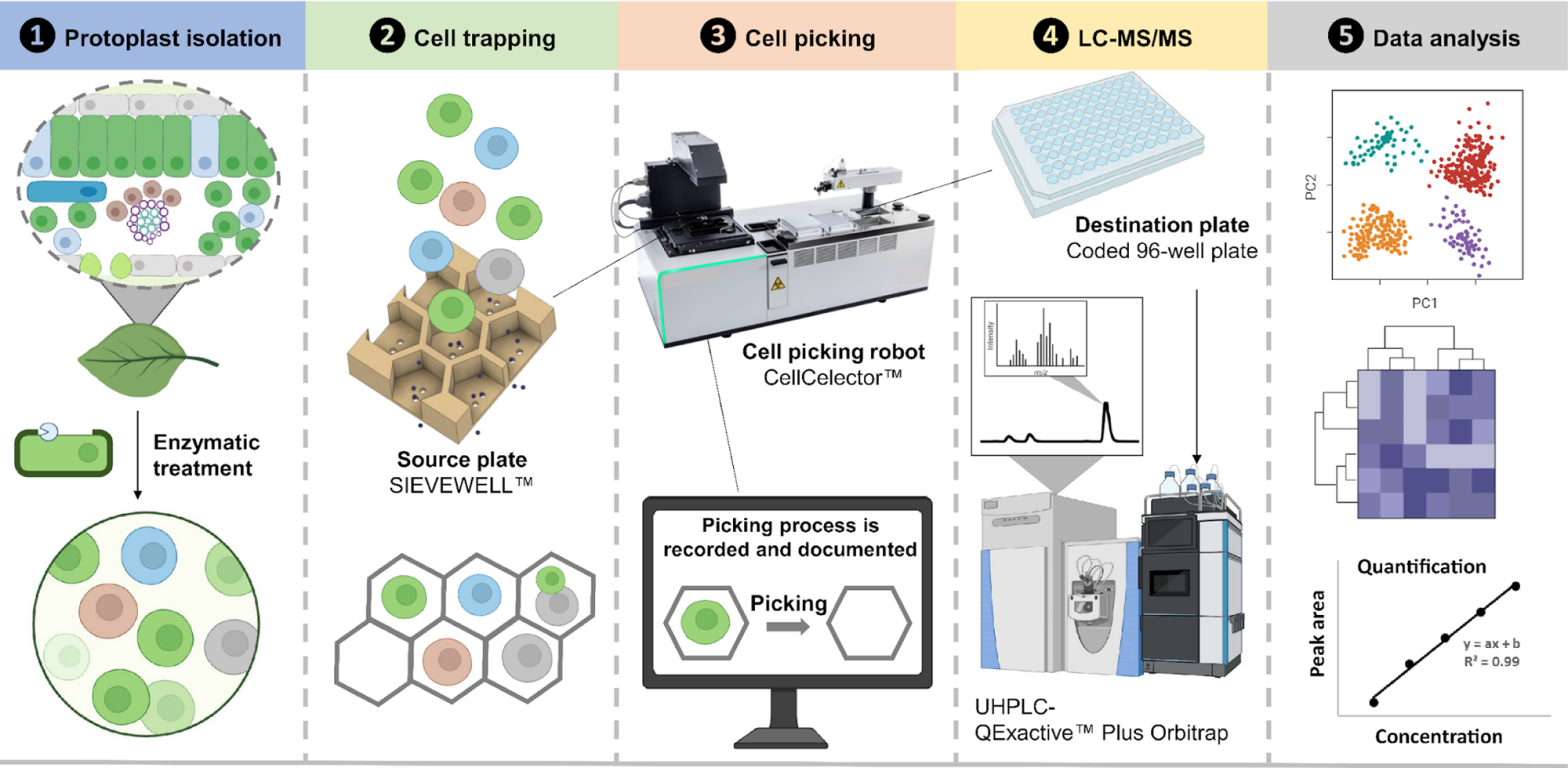
Workflow for the single-cell mass spectrometry (scMS) method described here. In step 1, protoplasts are isolated from leaf, root, or petal. Protoplasts are then trapped in the wells of a Sievewell plate (step 2). In step 3, individual cells are imaged and picked with a CellCelector robot. Single cells are transferred to 96-well plates, lysed, and subjected to LC-MS analysis (step 4). Targeted and untargeted mass spectrometry data are then processed to obtain quantitative and qualitative information (step 5). Created with BioRender.com.

Single-cell analysis uses protoplasts, which are cells in which the cell walls have been enzymatically disrupted. A method for obtaining healthy and viable protoplasts from leaf, root, and petal tissues of the Sunstorm Apricot (SA) cultivar was developed (see the Experimental section). In addition, we examined petal tissue of Little Bright Eyes (LBE) and Atlantic Burgundy Halo (ABH) cultivars (Figure 1b), since we anticipated that the petals of these differently colored cultivars would have different phenylpropanoid natural product profiles. Approximately 10 000 protoplasts were dispensed onto a microwell chip with cell-size micropores (50 μm) to capture single cells by gentle suction-induced sedimentation. Since the size of the protoplasts obtained from the tissues used in this study varied between 16 and 45 μm, 50 μm wide wells were used. Once situated in the micropores, cells were imaged by bright-field and fluorescence microscopy to record their size, morphology, and fluorescence (Figure S5b). In particular, we monitored the fluorescence signal as idioblast cells from *C. roseus* leaves display a characteristic blue fluorescence due to the accumulation of the alkaloid serpentine (**4**),^18^ and we also monitored the presence of colored cells, which accumulated anthocyanins (Figure S5c). Key parameters of the cell-picking process, such as the aspiration/dispensing speed and volume, were optimized to obtain a 95% success transfer rate with minimal cross-contamination. The cells were then collected in 96-well plates compatible with the autosampler of an UHPLC-HRMS system. Each 96-well contained 6 μL of 0.1% formic acid solution in water, which resulted in lysis of each transferred protoplast by osmotic shock. 6 μL of methanol containing ajmaline was added as an internal standard followed by mixing ensured complete disruption of the cells and release of the metabolites. 2 μL were used to prepare a pooled quality control sample. Collection of each single cell takes approximately 13 s, providing an output of one 96-well plate in ca. 20 min. Chromatographic conditions were optimized for rapid analysis (7 min per run) on a micro UPLC column (1 mm × 50 mm), allowing the analysis of approximately 180 cells per day (Figure 2). We previously showed that natural product profiles of *C. roseus* did not change substantially after protoplast isolation.^5^

### ScMS of Leaf-, Root-, and Petal-Derived Single Cells

**Figure 3:**
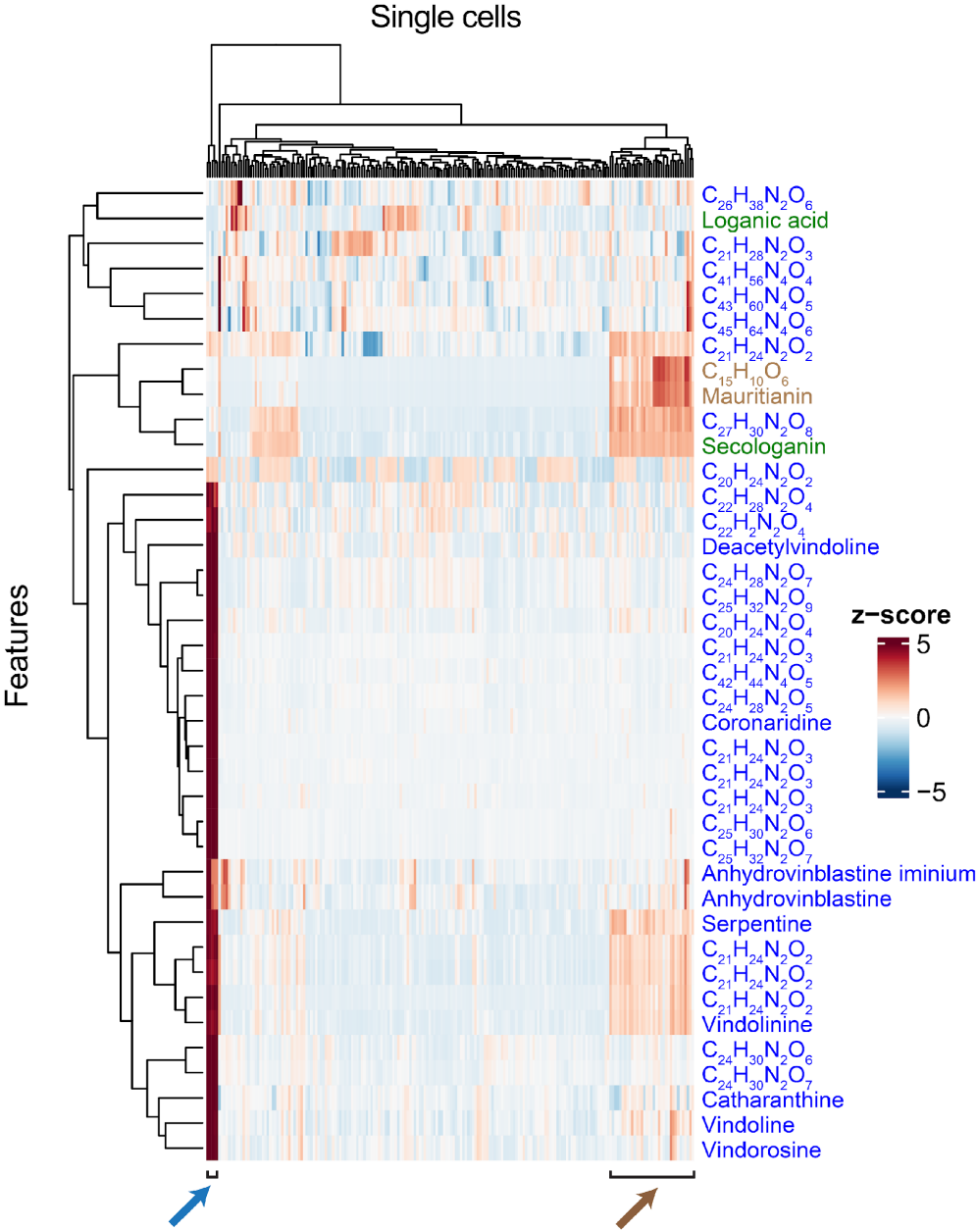
Hierarchical clustering analysis of 202 leaf protoplasts using a set of chemical features (39) that could be confidently assigned to iridoid (green compound names), alkaloid (blue compound names and formulas), or flavonoid (brown compound name and formula) types. The blue arrow indicates the group of cells with high levels of alkaloids. The brown arrow indicates the group of cells that accumulates primarily flavonoids and secologanin (**2**).

Untargeted scMS was performed on approximately 200 cells from each of the five tissue samples (Table S5 and Figures S6–S10). This untargeted metabolomic data processing pipeline extracted approximately 1000 features having a chemical formula assignment with less than a 2 ppm error (Table S5, Supporting Information Data 3). The chromatography method was optimized for the detection of alkaloid, iridoid, and phenylpropanoid natural products; primary metabolites such as amino acids and lipids were not captured in this analysis. The number of robust features identified in each analyzed cell was estimated from 10 randomly selected cells from each data set; in these representative cells, the number of features varied between 10 and 280, reflecting the difference in the natural product content among the cell population (Table S5). Hierarchical clustering analysis (HCA) was applied to the untargeted data sets in order to identify relationships between cells and metabolites, particularly the co-occurrence of different compounds in the same cell. When we applied HCA to a subset of 39 features identified in leaf protoplasts that could be confidently assigned as iridoid, phenylpropanoid, or alkaloid (Figure 3), it was immediately apparent that there was a small number of cells that were highly enriched in alkaloids, whereas a larger number of cells, accumulated flavonoids, secologanin (**2**), and a lower concentration of alkaloids. We repeated this analysis with roots and petals from each of the three varieties, again with features that could be confidently assigned as iridoid, phenylpropanoid, or alkaloid (Figures S11–S14). The analysis with root and petal protoplasts also showed the presence of a population of cells specializing in alkaloid accumulation, though specific patterns of flavonoid and secologanin (**2**) accumulation varied among these tissues (Figures S11–S14).

### Natural Product Concentrations Across Cell Populations

This scMS workflow allowed for simultaneous targeted and untargeted analysis of metabolites. Single-cell analysis was performed in full scan mode, allowing sufficient scanning events for quantification, while a pooled quality control (QC) sample was used for fragmentation analysis to permit structural characterization. To accurately identify and quantify levels of metabolites in a single cell, we used external calibration curves of the authentic standards to convert the measured peak area for each compound into an absolute quantity. Additionally, the diameter of each cell was measured from the images acquired during the cell picking, allowing us to estimate the volume of the cell, which was then used to calculate the concentration of each of these molecules in an individual cell (Supporting Information Data 4).

**Figure 4:**
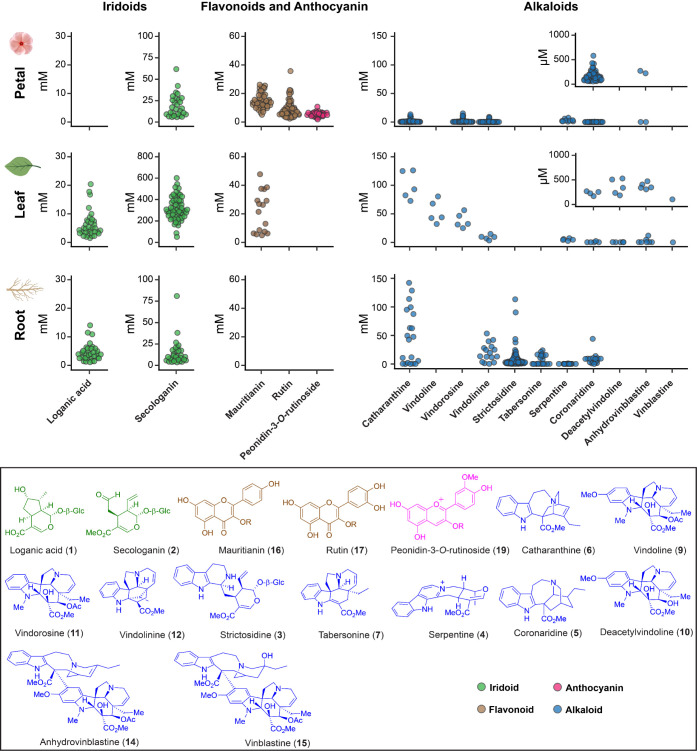
Quantification of 16 compounds in cells derived from leaf (202 cells), root (187 cells), and petal (232 cells) tissues. External calibration curves were generated using authentic standards. For information about the adducts considered in the quantification, the linearity range, and the LOQs, we refer to Tables S3 and S4. Plots show the concentration for each of these compounds in each cell. Colors of the data points represent the class of metabolites. All samples were from the Sunstorm Apricot (SA) cultivar. Created with BioRender.com.

Strikingly, many cells contained millimolar concentrations of the compounds subjected to targeted monitoring. The iridoid monoterpene secologanin (**2**) is by far the most abundant natural product observed in bulk leaf tissue (12.5 mg g^–1^ of fresh weight), and this is reflected by the fact that secologanin (**2**) is found at high concentrations (50–600 mM) in a high percentage (ca. 30%) of leaf cells sampled (Figure 4). Anhydrovinblastine (**14**), the precursor for vinblastine (**15**), is much less abundant in leaves, and this compound was observed in a range of 300 μM to 10 mM in a smaller number of cells (ca. 3% of all cells that were sampled). In contrast, vinblastine (**15**) is present at low levels in bulk tissue, and correspondingly, was observed at a maximum of 100 μM concentration in only one leaf-derived cell out of all cells analyzed (Figures 4 and S15).

High variability in the levels of all quantified compounds was observed in these cell populations from all five tissues. For example, secologanin (**2**) was detected at concentrations ranging from 5 to 60 mM in petal cells (Figure 4). Notably, although a few leaf and root cells accumulated catharanthine (**6**) to concentrations over 100 mM, the maximum concentration of catharanthine (**6**) that can be reached in solution (pH 5, the expected pH of the vacuole) is about 20 mM (see the Experimental section). Natural deep eutectic solvents have been proposed to aid in the solubilization of certain metabolites, such as anthocyanins in plants,^19−21^ and this may also be the case for alkaloids.

### Cell Type Specificity Compared Among Tissues

**Figure 5:**
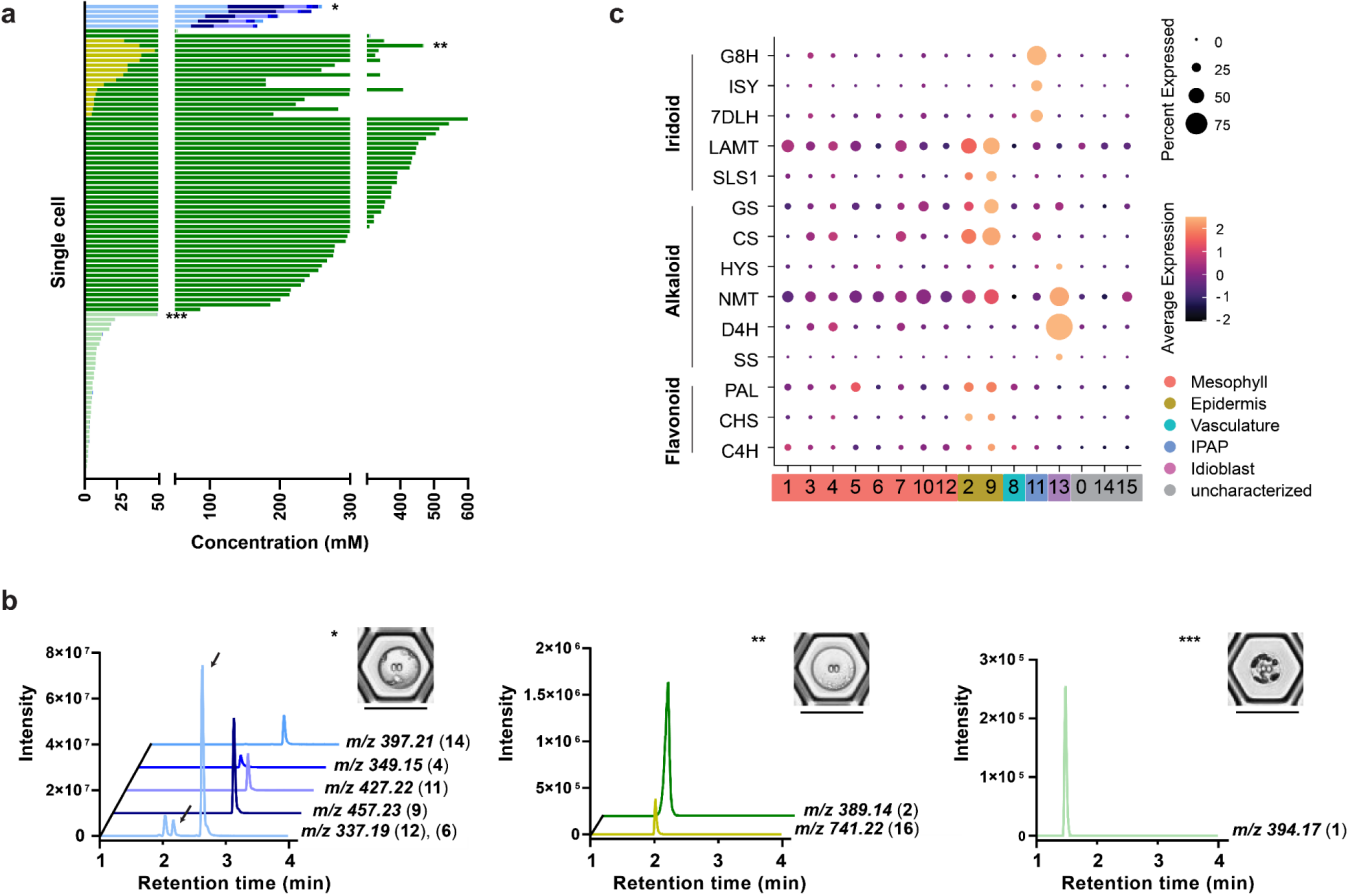
Metabolites found across a population of leaf-derived cells (202 cells). a, Stack plot showing the absolute concentration of each of the 16 quantified metabolites in each cell. Colors indicate classes of compounds: bars in blue shades represent alkaloids, green represents iridoids, and yellow represents flavonoids. Breaks in the *x*-axis indicate changes in the scale required to visualize both low- and high-abundant metabolites in the same graph. The asterisks indicate the cells for which the chromatograms are shown. b, The cell with one asterisk is an idioblast cell accumulating high amounts of alkaloids (*m*/*z* 337.19, vindolinine (**12**) and catharanthine (**6**); *m*/*z* 457.23, vindoline (**9**); *m*/*z* 427.22, vindorosine (**11**); *m*/*z* 349.15, serpentine (**4**); *m*/*z* 397.21, anhydrovinblastine (**14**); the cell with two asterisks is an example of epidermal cells in which both the iridoid secologanin (**2**) (*m*/*z* 389.14) and the flavonoid mauritianin (**16**) (*m*/*z* 741.22) coexist; the cell with three asterisks is an example of IPAP cell accumulating the iridoid loganic acid (**1**) (*m*/*z* 394.17). The scale bar is 50 μm. c, scRNA-seq data (Sunstorm Apricot leaf) for selected biosynthetic genes in iridoid, alkaloid, and flavonoid biosynthesis.

The untargeted metabolic analyses suggested the presence of subpopulations of cells that specialized in accumulating alkaloids or flavonoids (Figures 3 and S11–S14). To more accurately assess the ratio of compounds produced in each cell across the entire cell population that was measured, we used these quantitative data to generate stacked plots to show the absolute levels (mM) of each of the 16 quantified compounds in each cell (Figures 5a and S16b, S17, S20, and S21). In leaves, we observed a subpopulation of cells that specifically accumulate loganic acid (**1**) (Figure 5a). Since loganic acid (**1**) has been shown to be synthesized in internal phloem-associated parenchyma (IPAP) cells,^22^ we used the presence of this molecule as a marker for this cell type. Serpentine (**4**), an alkaloid that fluoresces under UV,^5,18^ was used as a marker for the assignment of idioblast cells, a rare cell type (2–3%^5^ of the total cell population) that accumulates the majority of the alkaloids. Therefore, the scMS data highlight that the high alkaloid levels observed in the bulk tissue are due to very few specialized cells containing large quantities of the compounds. Secologanin (**2**), which is observed in the bulk tissue at higher levels than any alkaloid, is detected in high concentrations in a much larger population of cells (Figure 5a).

To compare the gene expression of biosynthetic enzymes with metabolite locations at the cell-level resolution, we compared the scMS data with previously reported scRNA-seq data for *C. roseus* leaves.^5^ Notably, the partitioning of cells observed from the leaf-derived scMS data is only partially reflected in the expression of the biosynthetic genes from the scRNA-seq data (Figure 5c). While the gene expression data show that catharanthine (**6**) is synthesized in epidermal cells, this alkaloid almost exclusively accumulates in cells assigned as idioblasts (e.g., catharanthine (**6**) is found in the same cells as serpentine (**4**)), suggesting that an efficient transport mechanism of catharanthine (**6**) from epidermal to idioblast cells is in place. Additionally, the scMS data show two distinct populations of cells that accumulate secologanin (**2**) (green-colored bars, Figure 5a,b): one population that also accumulates the flavonoid mauritianin (**16**) (yellow-colored bars, Figure 5a,b) and one that does not contain quantifiable amounts of any flavonoid-like compounds (Figure 5a). This distinction is not readily apparent in the scRNA-seq data, as phenylpropanoid biosynthetic genes^23^ (e.g., PAL, CHS, and C4H) and secologanin (**2**) biosynthetic genes (LAMT and SLS) were detected in the same cell clusters by scRNA-seq (Figure 5c). Secologanin (**2**) may serve a defensive role in addition to being a biosynthetic intermediate;^24^ secologanin (**2**) may therefore be transported to additional cell types after synthesis to support the additional biological function of this molecule.^25^ On average, cells that accumulate both secologanin (**2**) and flavonoid have lower levels of secologanin (**2**) than cells that are specialized for secologanin (**2**) (Figure 5a).

We also compared scMS profiles between root and leaf tissues (Figure S16). As in leaves, roots have cell subpopulations that specialize in accumulating alkaloids (e.g., catharanthine (**6**)). We also identified the root-specific alkaloid hörhammercine (**8**) (that is derived from tabersonine (**7**) (Figure 1a). Although this could not be quantified accurately due to the scarcity of the authentic standard, we could definitively determine that this alkaloid colocalized with catharanthine (**6**) (Figure S11), providing further support for the observation that alkaloids accumulate in specialized cell types in roots analogous to leaves. A second cell subpopulation accumulates iridoids and the upstream alkaloid strictosidine (**3**), while a third subpopulation of cells accumulates only strictosidine (**3**) but no iridoids. Flavonoids were not detected in root-derived cells. These three distinct populations are not apparent from the scRNA-seq data (previously reported in ref^5^), which shows that iridoid and alkaloid biosynthetic genes are both expressed in ground cells (Figure S16a). Therefore, the root scMS data reveal cell-type specificity that cannot be detected from the scRNA-seq data. The mechanism by which the pattern of metabolite accumulation observed in the scMS data set is established remains to be determined.

Finally, we examined flower petals, which contain flavonoids, anthocyanins, iridoids, and alkaloids. In cells derived from petals of the SA cultivar, the majority of cells sampled are highly enriched in either rutin (**17**) (flavonoid)/peonidin 3-*O*-rutinoside (**19**) (anthocyanin), the flavonoid mauritianin (**16**), or a combination of monoterpene indole alkaloids (Figure S17). We also observe a fourth smaller subpopulation of cells that are specialized in secologanin (**2**) accumulation. To compare the scMS data with gene expression profiles, we also generated a scRNA-seq data set for petals, since this data set had not been previously reported (Figure S18 and Supporting Information Data 5). Surprisingly, many alkaloid and iridoid biosynthetic genes are expressed at negligible levels in flower petals (Figure S18). This was consistent with bulk RNA-seq data taken at time points after flower opening (Figure S19 and Supporting Information Data 6). Petals provide a striking case in which the prevalence of the metabolites – which are found in high levels in this tissue – is not correlated with gene expression. The metabolites, or biosynthetic intermediates of these metabolites, that are detected in petal-derived cells may be synthesized during different stages of flower development, or alternatively, these compounds could be transported from other tissues.

We also investigated petal-derived cells of LBE and ABH cultivars by scMS (Figures S20 and S21). In both LBE and ABH, cells that are specialized to accumulating alkaloids are observed. However, while SA and ABH have cells that specialize in secologanin (**2**) accumulation, in LBE, secologanin (**2**) nearly always colocalizes with flavonoids. Of all three cultivars, LBE has the highest levels of alkaloids in petals, and this is reflected in the single-cell data, with concentrations of 300–400 mM being reached for the total alkaloid level (Figure S20). The mechanisms by which these localization patterns are achieved, or whether these different metabolite colocalization patterns have functional or ecological significance, remain to be determined. Nevertheless, this scMS analysis clearly shows that leaves, roots, and petals of the three cultivars store metabolites differently at the cell-type level.

**Figure 6:**
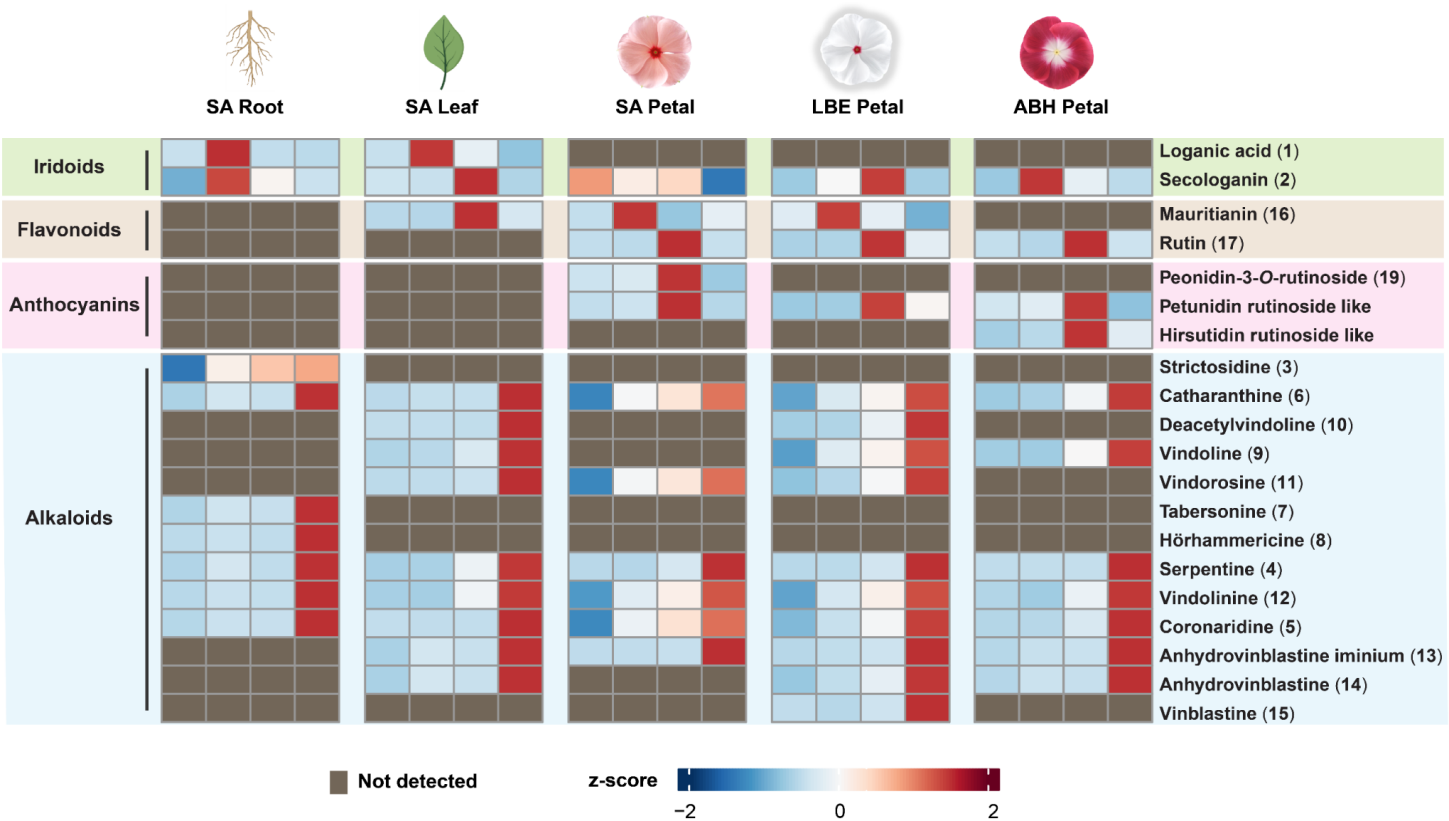
Comparison of the metabolite profiles of cell populations across tissues and cultivars. Cells were grouped into four clusters based on the levels of the 20 metabolites that could be confidently assigned using *k*-means clustering analysis (Figure S22). The intensity of the compounds represented in the heat map is obtained by measuring, log-transforming, normalizing according to the calculated cell volumes, and averaging the peak area of all cells in one cluster. The identities of all compounds except two were validated with an authentic standard. Authentic standards are not available for petunidin rutinoside-like and hirsutidin rutinoside-like compounds. The number of cells analyzed for each tissue is reported in Table S5. Created with BioRender.com.

To more easily visualize the differences in metabolite cell-type specificity across these five samples, we grouped the cell populations of each tissue into four clusters (using *k*-means clustering analysis) based on the peak area of the 20 natural products that could be structurally assigned with high confidence (Figures 6 and S22). While these 20 compounds represent only a small fraction of the natural product profile diversity of *C. roseus*, this analysis shows that all tissues have cells that specialize in monoterpene indole alkaloid accumulation. However, iridoid, flavonoid, and anthocyanin compounds show different colocalization patterns across these tissues.

## Discussion

The medicinal plant *C. roseus* produces a wide array of natural products, including iridoid-type monoterpenes, flavonoids, anthocyanins, and monoterpene indole alkaloids.^26,27^ The importance of two of these alkaloids, anhydrovinblastine (**14**) and vinblastine (**15**), which are used in cancer treatment,^28^ has made *C. roseus* one of the most studied medicinal plants. The discovery and engineering of the biosynthetic pathways responsible for construction of complex natural products such as vinblastine (**15**) is of paramount importance to improve access to these valuable plant-derived molecules (e.g., Zhang. J. et al.).^29^ The availability of scRNA-seq data sets, which reveal how biosynthetic elements are expressed in specific cell types, is transforming how we elucidate and engineer plant-derived natural product pathways. However, since the ultimate cellular location of natural products is not always accurately predicted by scRNA-seq, these transcriptomic data sets only provide a partial snapshot of natural product biosynthesis. A complete picture requires a comparison of metabolite and biosynthetic gene localization. Here, we report a method for single-cell mass spectrometry-based metabolomics (scMS) that can detect and quantify the iridoid-type monoterpenes, flavonoids, anthocyanins, and monoterpene indole alkaloids in individual cells from three different tissues (leaf, root, and petal) of *C. roseus*. Although we use a Q-Exactive Plus Orbitrap mass spectrometer in the work presented here, this scMS workflow is compatible with a variety of mass spectrometry instruments, making this method accessible to many laboratories. This approach is also, in principle, applicable for the detection of any metabolites that can be detected using LC-MS.

We performed both targeted and untargeted scMS analyses for leaf, root, and petal cells and then compared each of these scMS data sets to the corresponding scRNA-seq data from the same tissue. The comparison of the scMS and scRNA-seq data sets in each tissue highlighted distinct differences between metabolite location and biosynthetic gene expression. In leaves, the scMS data showed three major populations of cells, largely correlating with the scRNA-seq data. However, we saw that some alkaloids (e.g., catharanthine (**6**)) were transported to a different cell type. Additionally, we noticed two distinct populations of cells that contained secologanin (**2**) that were not immediately apparent from the biosynthetic gene expression profiles observed in the scRNA-seq data. Additionally, a comparison of scMS and scRNA-seq data of root and petal suggested that in these tissues, the expression of biosynthetic genes does not always correlate with the site of metabolite accumulation. The expression of biosynthetic genes in root suggested that iridoids and downstream alkaloids would be found in the same cell type (ground cells), but the scMS data showed that alkaloids are not found in the same cell type as the upstream iridoids. Although high levels of alkaloids were observed in petal cells, the biosynthetic gene expression was low, suggesting that these compounds are transported from other tissues, or that synthesis occurs at different stages of flower development. Overall, the comparison of scRNA-seq and scMS data strongly suggests the presence of many active intercellular transport processes. To date, only one intercellular transporter has been identified in *C. roseus*, the transporter responsible for the movement of loganic acid (**1**) from IPAP cells to epidermal cells.^25^ Therefore, these scMS and scRNA-seq data provide the foundation for the discovery of additional transporters.

The targeted scMS data show that individual cells can accumulate exceptionally high (>100 mM) concentrations of many of these natural products. Glucosinolates may also reach millimolar levels in cells,^30,31^ but this is indirectly inferred from bulk tissue measurements. Most of the metabolites that accumulate to these high concentrations, such as secologanin (**2**) and vindoline (**9**), are predicted to be localized in the vacuole, highlighting the storage capacity of this plant organelle.^32,33^ The high levels of loganic acid (**1**) may also accumulate because transport from IPAP to epidermal cells is a rate-determining step. Iridoids, monoterpene indole alkaloids, and phenylpropanoids all have unstable biosynthetic intermediates in the pathway, but none of these were observed in these scMS experiments. ScMS with protoplasts fed with isotopically labeled precursors may facilitate the identification of lower abundance compounds in these experiments.

Both targeted and untargeted MS suggest that a majority of monoterpene indole alkaloids accumulate in specialized cells that represent only a small fraction of the total cell population. The iridoid secologanin (**2**) is found in high concentrations across a larger fraction of sampled leaf cells, highlighting the capacity of the plant cell factory to produce and store exceptionally high levels of a range of complex natural products. The most medicinally valuable alkaloid, vinblastine (**15**), is found at micromolar concentrations in only a small fraction of cells that were sampled, which reflects the low levels observed in the bulk tissue.

## Conclusion

Although single-cell sequencing is now widely used in plants, rigorous structural characterization and quantification of metabolites in single cells have proven to be challenging. Here, we report a robust method for single-cell mass spectrometry in which we identify and quantify the concentrations of 16 metabolites across four natural product classes in individual cells of leaves, roots, and petals of the medicinal plant *C. roseus*. In combination with the scRNA-seq data, this scMS approach allows us to dissect the logistics of these pathways at a highly resolved level. The incorporation of this robust scMS pipeline into a single-cell omics analysis pipeline can be a step change in our understanding of the biosynthesis and biological function of these molecules.

## Supporting Information Available

Additional experimental details; materials and methods, including all cell picking and mass spectrometry parameters; untargeted metabolomic analysis (Figure S1); chemical structures of the compounds identified in this study (Figure S2); quantification of iridoids, flavonoids, anthocyanins, and alkaloids (Figures S3, S4, and S15); microscopic photos of representative tissue sections (Figure S5); heatmap representing untargeted metabolomic data (Figures S6–S10); heatmap showing the distribution of chemical features (Figures S11–S14); single-cell mRNA data (Figures S16 and S18); ratio of compounds found across the population of petal cells (Figures S17, S20, and S21); expression levels of key genes (Figure S19); PCA of single cells (Figure S22); definition of enzyme abbreviations (Table S1); validation of the reproducibility of the LC-MS method (Table S2); compounds used for identification (Table S3); analytical parameters of the compounds (Table S4); summary of parameters of scMS (Table S5); list of all chemicals used in this study (Table S6); Compound Discoverer important parameters (Table S7); MS^2^ spectral data for all compounds identified in this study (PDF)
Bulk untargeted analysis dataset (Data 1); feature classification into three main chemical classes using SIRIUS (Data 2); single-cell untargeted analysis dataset (Data 3); quantification analysis dataset for single cells (Data 4); source data for Figure S18 (Data 5); source data for Figure S19 (Data 6) (XLSX)
