# Supplementary material for: Quantitative Single-Cell Mass Spectrometry Provides a Highly Resolved Analysis of Natural Product Biosynthesis Partitioning in Plants
Source: J Am Chem Soc. 2024 Aug 14;146(34):23891–900. doi: 10.1021/jacs.4c06336 (PMC11363012; doi:10.1021/jacs.4c06336)
Supplement: Supplementary file 1 — ja4c06336_si_001.pdf [file ja4c06336_si_001.pdf]

Supporting Information

Quantitative single cell mass spectrometry provides a highly resolved analysis of natural product biosynthesis partitioning in plants

Anh Hai Vu[1], Moonyoung Kang[1], Jens Wurlitzer[1], Sarah Heinicke[1], Chenxin Li[2,3], Joshua C. Wood[2], Veit Grabe[4], C. Robin Buell[2,3,5*], Lorenzo Caputi[1*], Sarah E. O'Connor[1*]

[1]*Department of Natural Product Biosynthesis, Max Planck Institute for Chemical Ecology, 07745, Jena, Germany* [2]*Center for Applied Genetic Technologies, University of Georgia, 30602, Athens, GA, USA* [3]*Department of Crop and Soil Sciences, University of Georgia, Athens, 30602, GA, USA* [4]*Microscopic Imaging Service, Max Planck Institute for Chemical Ecology, 07745, Jena, Germany* [5]*Institute of Plant Breeding, Genetics, and Genomics, University of Georgia, 30602, Athens, Georgia, USA.*

Table of Contents

Pages

Experimental S4 – S8

Supplementary Figures S9 – S30

Figure S1. Untargeted metabolomic analysis of *C. roseus* SA leaf, root, and petal bulk tissue extracts.

Figure S2. Chemical structures of the compounds identified in this study.

Figure S3. Quantification of iridoids, flavonoids, and the anthocyanin peonidin-3-*O*-rutinoside in bulk tissues.

Figure S4. Quantification of alkaloids in bulk tissues.

Figure S5. Microscopic photos of representative tissue sections.

Figure S6. Heatmap representing untargeted metabolomic data of 202 leaf-derived protoplasts from the Sunstorm Apricot cultivar.

Figure S7. Heatmap representing untargeted metabolomic data of 187 root-derived protoplasts from the Sunstorm Apricot cultivar.

Figure S8. Heatmap representing untargeted metabolomic data of 232 petal-derived protoplasts from the Sunstorm Apricot cultivar.

Figure S9. Heatmap representing untargeted metabolomic data of 241 petal-derived protoplasts from the Little Bright Eyes cultivar.

Figure S10. Heatmap representing untargeted metabolomic data of 244 petal-derived protoplasts from the Atlantis Burgundy Halo cultivar.

Figure S11. Heatmap showing the distribution of chemical features among the clusters (Sunstorm Apricot root protoplasts).

Figure S12. Heatmap showing the distribution of chemical features among the clusters (Sunstorm Apricot petal protoplasts).

Figure S13. Heatmap showing the distribution of chemical features among the clusters (Little Bright Eyes petal protoplasts).

Figure S14. Heatmap showing the distribution of chemical features among the clusters (Atlantic Burgundy Halo petal protoplasts).

Figure S15. Quantification of iridoids, flavonoids, anthocyanins, and alkaloids in single cells isolated from flower petals from three different varieties.

Figure S16. Single cell mRNA data of root protoplasts (Sunstorm Apricot cultivar).

Figure S17. Ratio of compounds found across the population of petal cells (Sunstorm Apricot cultivar).

Figure S18. Single cell mRNA data of Sunstorm Apricot (SA) petals.

Figure S19. Expression levels of key genes in Sunstorm Apricot petals at different stages of flower opening (bulk RNAseq data).

Figure S20. Ratio of compounds found across the population of petal cells (Little Bright Eyes cultivar).

Figure S21. Ratio of compounds found across the population of petal cells (Atlantic Burgundy Halo cultivar).

Figure S22. PCA of single cells using only the compounds identified by co-elution with a reference standard.

Supplementary Tables S31 – S37

Table S1. Definition of enzyme abbreviations.

Table S2. Validation of the reproducibility of the LC-MS method. Results are presented as RSD% of the peak area and retention time.

Table S3. Compounds used for identification.

Table S4. Analytical parameters of the compounds quantified in this study.

Table S5. Summary of parameters of scMS for 5 tissues.

Table S6. List of all chemicals used in this study.

Table S7. Compound Discoverer™ important parameters.

Supplementary Data S38 – S61

Supplementary MS^2 Spectra. Fragmentation data from all compounds identified in this study.

Experimental

Chemicals

All solvents used in this study were of UPLC/MS grade. Information about chemicals and reagents is listed in Table S6.

Plant growth conditions

Catharanthus roseus (*C. roseus*) plants of Sunstorm Apricot (SA), Little Bright Eyes (LBE), and Atlantis Burgundy Halo (ABH) cultivars were germinated and grown in a York chamber at 23 °C, under a 16h/8h light/dark cycle.

Protoplast isolation

Leaf protoplast isolation

A healthy plant was watered and left in the dark the day before the leaves were harvested. Three leaves of ca. 3 cm in length (Figure 1b) were selected, rinsed gently with water, and cut in 1 mm strips with a sterile surgical blade. The leaf strips were immediately transferred to a Petri dish with 10 mL of digestion medium (2% (w/v) Cellulase Onozuka R-10, 0.3% (w/v) macerozyme R-10, and 0.1% (v/v) pectinase dissolved in Mannitol-MES (MM) buffer. MM buffer contained 0.4 M mannitol and 20 mM MES, pH 5.7-5.8, adjusted with 1 M KOH. The open Petri dish was put inside a desiccator and 100 mBar vacuum was applied for 15 min to infiltrate the medium into the leaf strips. The vacuum was gently disrupted for 10 s after every 1 min. The leaf strips were then incubated in the digestion medium for 2.5 h at room temperature. After the incubation, the Petri dish was placed on an orbital shaker at ca. 70 rpm, for 30 min at room temperature to help release the protoplasts. The protoplast suspension was filtered through nylon sieves of 70 µm and then 40 µm to remove cellular debris. After that, the suspension was transferred to two 15 mL conical tubes. The protoplast suspension was centrifuged at 70 x g with gentle acceleration/deceleration, for 5 min, at 23 ^{o}C to pellet the protoplasts. The supernatant was removed and the protoplasts were washed three times with 5 mL of MM buffer. Finally, the protoplasts were pooled together and resuspended in 1 mL of MM buffer. The protoplast concentration and viability were determined using a haemocytometer and fluorescein diacetate staining, respectively. The final concentration of protoplasts was adjusted to 10^6 protoplasts in 1 mL.

Petal protoplast isolation

The petals were removed from fully opened flowers (stage 1, Figure S19) and cut into 1 mm strips with a sterile surgical blade. Protoplast isolation was performed using the same protocol used for the leaves, except for the time of incubation in the digestion medium, which was decreased to 1.5 hours.

Root protoplast isolation

Roots from a young healthy plant (6-7 weeks old) were used for protoplasting (Fig. 1b). After removing the soil, the roots were washed with water and gently dried to avoid damaging the tissue. The roots were then finely sliced with a sterile surgical blade. Protoplast isolation was performed using the same protocol used for the leaves, with a few modifications: the concentration of macerozyme in the digestion solution, infiltration time, incubation time, and centrifugation speed were optimized to 0.6%, 30 minutes, 1.5 hours, and 200 g, respectively, for root protoplasting.

Single cell picking

SIEVEWELL™ chips (Sartorius) with 90,000 nanowells (50 µm x 50 µm, depth x diameter) were used for single-cell trapping and sorting. Chips were primed with 100% ethanol and washed twice with MM buffer. The chips were then incubated with 5% BSA in MM buffer for 30 minutes at room temperature. Subsequently, the 5% BSA in MM buffer solution was discarded through the side port and replaced with MM Buffer. Finally, 1 mL of diluted protoplasts suspension (approximately 10,000 cells) was carefully added and dispensed in a Z-shape across the chip. Liquid (1 mL) was discarded from the side ports.

The SIEVEWELL™ chip was then mounted on the CellCelector™ Flex (Sartorius) instrument and the cells were visualized using the optical unit, constituted by a fluorescence microscope (Spectra X Lumencor) and a CCD camera (XM-10). Photos in bright-field or fluorescence (DAPI filter) were acquired, depending on the experiment. Single protoplasts were picked together with 20 nL of well solution using a 50 µm glass capillary and dispensed into SureSTART™ WebSeal™ 96-Well Microtiter plates (Thermo Fisher Scientific) containing 6 µL of MilliQ water with 0.1% formic acid. Pictures of the nanowells before (containing a single cell) and after picking (without a cell) were recorded. After picking, 6 µL of MeOH containing 20 nM ajmaline (internal standard) was added to each well. Pooled QC samples consisting of 2 µL of each sample were made for each experiment and used for quality control and for MS^2 (fragmentation) analysis.

Preparation of bulk tissue extracts

We performed untargeted and targeted mass spectrometry analysis of bulk tissue extracts of leaves, roots, and petals of *C. roseus* SA, a cultivar with peach petals (Figure 1b). Bulk tissue samples were diluted so that the concentration of the alkaloid catharanthine matched the concentration ranges observed in the single cell datasets. We also characterized the metabolic profiles of petals from LBE and ABH cultivars, with white- and burgundy-colored petals, respectively (Figure 1b). All tissues were ground to a fine powder using a Tissuelyser II (Qiagen). Metabolites were extracted with pure MeOH containing 2 µM ajmaline as an internal standard at a ratio of 300 µL of solvent per 10 mg of tissue. After vortexing and sonication for 10 min, the tissue extracts were filtered through a 0.2 µm PTFE filter. The extracts from leaf, root, and petal were diluted 500-fold, 200-fold, and 50-fold, respectively before the analysis. Pooled QC samples of the extracts were also prepared.

LC-MS analysis

UHPLC-HRMS analysis was performed on a Vanquish (Thermo Fisher Scientific) system coupled to a Q-Exactive Plus Orbitrap (Thermo Fisher Scientific) mass spectrometer. For metabolite separation, a Waters™ ACQUITY UPLC BEH C18 130 Å column (1.7 µm, 1 mm x 50 mm) was used at a temperature of 40 °C. The binary mobile phases were 0.1% HCOOH (formic acid) in MilliQ water (aqueous phase) (A) and acetonitrile (ACN) (B). The gradient elution started with 1% ACN and increased linearly to 70% ACN over 5 minutes. The wash stage was performed at 99% ACN for 0.5 minutes before switching back to 1% ACN for 1.5 minutes to condition the column for the next injection. Total time for chromatographic separation was 7 minutes. The flow rate was 0.3 mL min^{-1} during the chromatographic separation. Injection volume was 4 µL. The autosampler was kept at 10 °C throughout the analysis. The needle in the autosampler was washed using a mixture of methanol and MilliQ water (1:1, v:v) for 20 seconds after draw and at a speed of 50 µL s^{-1}.

The Q-Exactive Plus Orbitrap mass spectrometer (Thermo Fisher Scientific) was equipped with a heated electrospray ionization (HESI) source. The mass spectrometer was calibrated using the Pierce positive and negative ion mass calibration solution (Thermo Fisher Scientific). The operating parameters of HESI were based on the UHPLC flow rate of 0.3 mL min^{-1} using source auto default: sheath gas flow rate at 48; auxiliary gas flow rate at 11; sweep gas flow rate at 2; spray voltage +3,500 V; capillary temperature at 256 °C; auxiliary gas heater temperature at 413 °C; and S-lens RF level at 50. Acquisition was performed in full scan MS mode (resolution 70,000-FWHM at 200 Da) in positive mode over the mass range *m/z* from 120 to 1,000. The full-scan and data-dependent MS/MS mode (full MS/dd-MS^2 Top10) was used for QC pooled samples to simultaneously record the spectra of the precursors as well as their MS/MS (fragmentation). Besides, the full MS/dd-MS^2 mode with inclusion list was also applied for the pooled QC samples to confirm fragments of the selected precursors. The parameters for dd-MS^2 were set up as follows: resolution 17,500, mass isolation window 0.7 Da and normalized collision energy (NCE) was set at 3 levels: 15%, 30%, and 45%. Spectrum data format was centroid. All the parameters of the UHPLC-HRMS system were controlled through Xcalibur software version 4.3.73.11 (Thermo Fisher Scientific).

Method partial validation and quantification

The repeatability of the method was tested by evaluating the stability of the retention time and detected peak areas of selected compounds during ten injections of the bulk methanolic extracts of three different tissues for three consecutive days. The inter-day precision was performed by applying the intra-day injections for three consecutive days (Table S2).

Alkaloids, iridoids, flavonoids and anthocyanins standard solutions were prepared in MeOH at a concentration of approximately 1 mM (exact concentration was recorded). Serial dilutions were made until 0.001 nM and analyzed by UHPLC-MS to determine limit of quantification (LOQ) and

calibration range (Table S4). Each calibration point was measured in triplicate. The extracted peak areas were used to calculate linear regression curves. Chromatographic peak area from extracted ion chromatograms (EIC) were integrated and extracted using the Xcalibur Quan Browser version 4.3.73.11 (Thermo Fisher Scientific).

LC-MS data processing and analyzing

Raw data were imported into Compound Discoverer™ software 3.2 (Thermo Fisher Scientific) for peak picking, deconvolution and formula assignment. Parameters are listed in Table S7.

The identity of selected ions was confirmed by comparison of their retention time and fragmentation spectra with analytical standards. Assignment of putative identities was performed with other glucosides with the same aglycon for two additional anthocyanin compounds. All the relevant features with MS^2 data were further analyzed with SIRIUS 5 software[1]. CANOPUS[2] was used to predict compound classes from mass spectra. Chemical classification of all features was performed using NPclassifier[3] results and only classification with natural product pathway probabilities of > 0.8 was selected.

Catharanthine solubility experiment

14.07 mg of catharanthine powder was dissolved in 20 µL of DMSO. After all the powder was dissolved, the catharanthine solution was added in 0.1 µL additions to 100 µL acetate buffer 0.1 M, pH 5. The maximum volume of catharanthine solution that could be dissolved in the buffer was 1 µL. The final concentration of dissolved catharanthine was approximately 21 mM.

Bioinformatics analysis

The feature list from Compound Discoverer™ was exported in .xlsx format. The peak areas were log-transformed and then scaled before performing principal components analysis (PCA) for reducing dimension, hierarchical clustering, and k-means clustering analysis[4]. Statistical analyses were performed using R (version 4.3.1). The heatmaps were visualized by pheatmap package (version 1.0.12) and ComplexHeatmap package (version 2.18.0)[5]. Violin point graphs were visualized by the ggplot2 (version 3.5.0) package. Stacked bar charts were visualized by GraphPad Prism software (version 9.5.1).

Bulk RNA sequencing of petals

Petals were collected from flowers at three different stages of development (Figure S19) and flash frozen in liquid nitrogen. Three biological replicates were prepared for each stage. After grinding with a Tissuelyser II (Qiagen), the total RNA was extracted using the RNeasy Plant kit (Qiagen) and sequenced by Biomarkers Technologies GmbH on an Illumina Novoseq X (PE150) platform.

Single Cell RNA-sequencing

Leaf and Root

For assessing single-cell transcriptome of leaf and root tissues, we reanalyzed 10x Genomics datasets from the previous study of *C. roseus*[6] which is publicly available at https://doi.org/10.5061/dryad.d2547d851. Downstream analysis and visualization were performed as described previously.

Petal

Protoplasts from *C. roseus* SA petals were isolated as described above, using four flowers for each biological replicate. ScRNA-Seq libraries were constructed using the PIPseq v4.0 Plus kit (Fluent Biosciences) according to the manufacturer's instructions, targeting ~ 3,154 (cro_bz), and ~ 3,306 cells (cro_ca)[7]. The libraries were sequenced on an Element Biosciences Aviti Instrument. For processing reads, pipseeker v3.1.3 (Fluent Biosciences) barcode pipeline was adopted to trim read 2 and detect barcode whitelist. Reads were then aligned to *C. roseus* (v3.0[6]) genome using the STARsolo pipeline (v2.7.10) with following parameters[8]: --soloBarcodeReadLength 0 --alignIntronMax 5000 --soloUMIlen 12 --soloCellFilter EmptyDrops_CR --soloFeatures GeneFull --soloMultiMappers EM --soloType CB_UMI_Simple --soloCBwhitelist using the barcode whitelists detected by pipseeker. Ambient RNA was removed by DecontX[9], and cells harboring RNA between 300 and 6,000 were kept for downstream analyses in seurat v5.0.1[10]. After log-normalization, top 3,000 variable genes were selected for integrating two replicates. Uniform manifold approximation and projection (UMAP) were calculated using the first 30 principal components and the same clustering parameters as leaf and root dataset. Cell types were annotated based on marker genes (vasculature, epidermis, idioblast) of previous studies[6, 11], and functional annotations of *de novo* marker genes (epidermis, parenchyma) in flower dataset.

Microscopy

Plant organ micrographs were acquired as tile scans (2x2 or 3x3) with a AXIO Zoom.V16 (Zeiss) equipped with a PlanApo Z 0.5x objective and a custom light box for homogenous indirect illumination. The tile scans were aligned and fused in ZEN (Zen lite 3.4, Zeiss) and background correction was done in Photoshop (Creative Cloud 2024, Adobe Inc.) after exporting.

Plant organ sections (approximately 50 µm thick) were prepared with a manual rotation Plant microtome (NK System MTH-1) and micrographs were subsequently acquired using an Apo Z 1.5x objective (Zeiss) with transmitted light illumination at the AXIO Zoom.V16. Micrographs of the protoplasts were acquired at an Imager.Z1 (Zeiss) equipped with a 20x/0.8 Plan-APOCHROMAT objective and DIC illumination. Lambda scans of the protoplasts were acquired with a cLSM 880 (Zeiss) equipped with a 20x/0.8 Plan-APOCHROMAT objective and 405nm laser diode (10% transmission) or 633 nm Helium-Neon laser (50% transmission) for excitation. Emission spectra were detected in lambda mode with a 9 nm binning ranging from 414 nm to 655 nm and 750 nm detector gain. The displayed micrographs are coded to show the actual color of the maximum intensity signal for each pixel.

Supplementary Figures

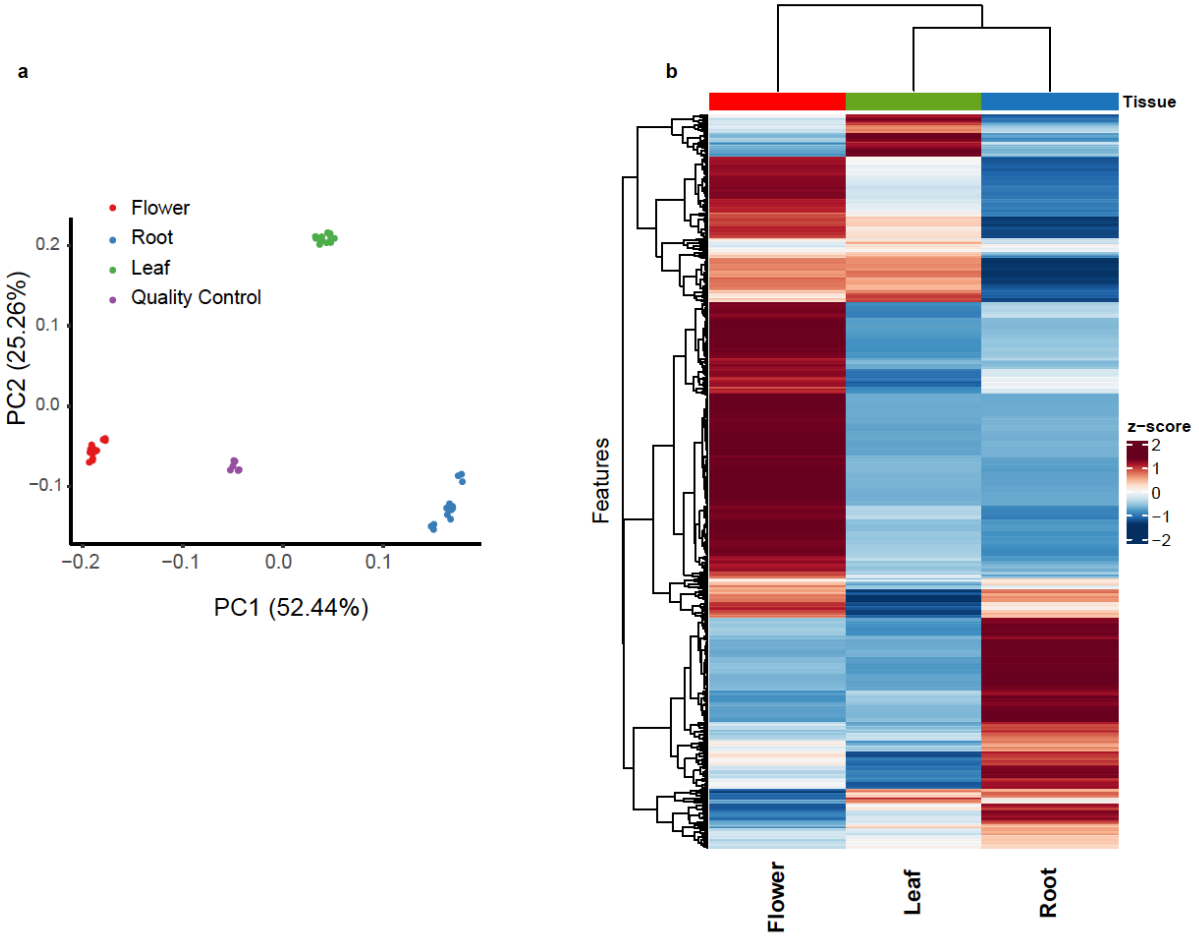

Figure S1. Metabolomic analysis of *C. roseus* SA leaf, root, and petal bulk tissue extracts. **a**, PCA plot of the chemical features (formula assignment) shows that the chemical composition varies substantially among the three tissues. **b**, Heatmap showing the occurrence of the different features across the three tissues. 5 biological replicates, 3 technical replicates for each biological replicate and 1014 features are listed (Supplementary Data 1).

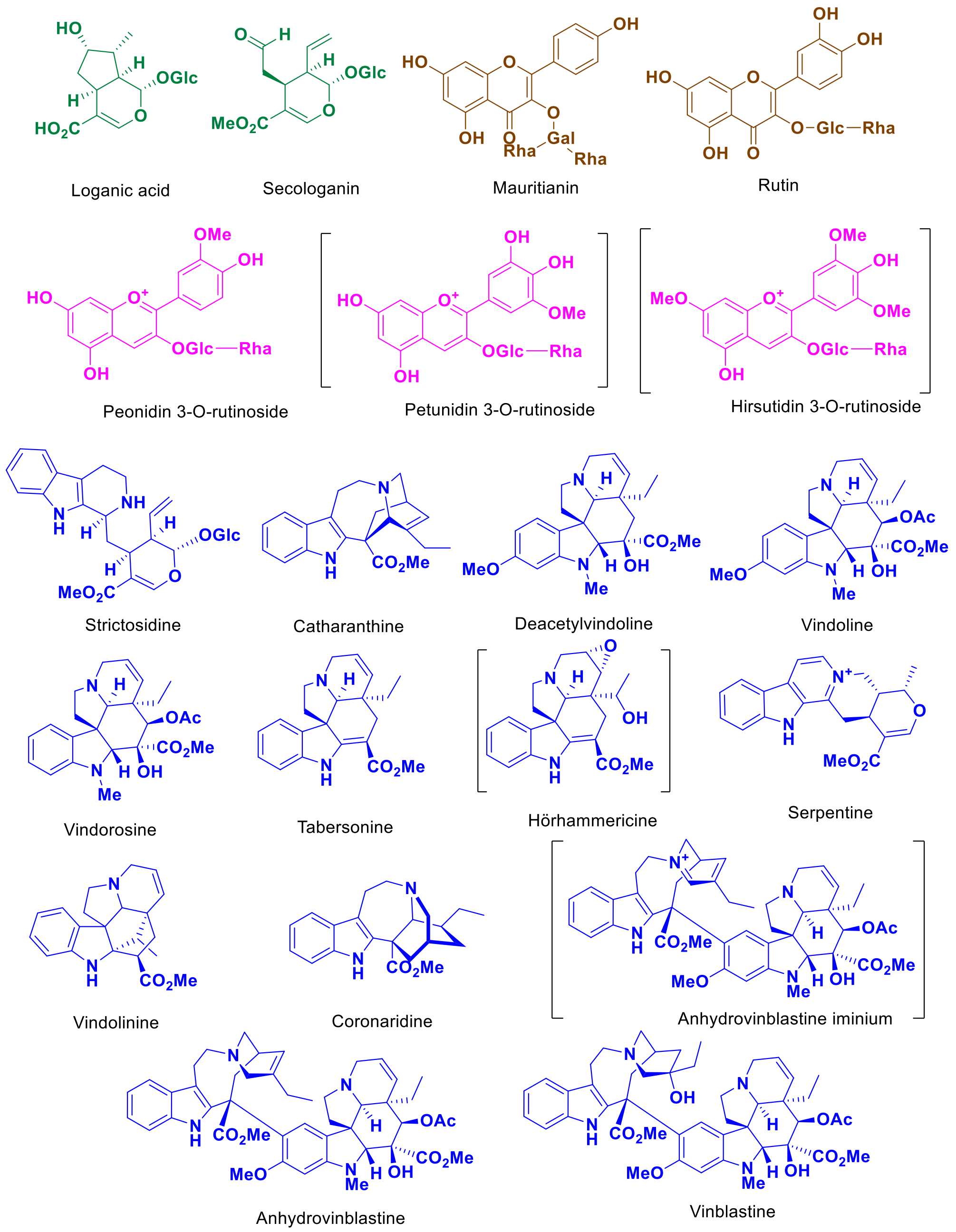

Figure S2. Chemical structures of the compounds identified in this study. The compounds in brackets were not quantified. Petunidin-3-*O*-rutinoside and hirsutidin-3-*O*-rutinoside are structural assignments based on mass and fragmentation. Authentic standards for anhydrovinblastine iminium and hörhammercine are available, but not in quantities sufficient for external calibration curves needed for quantification. All compounds not in brackets were quantified in the scMS studies using external calibration curves of authentic standards.

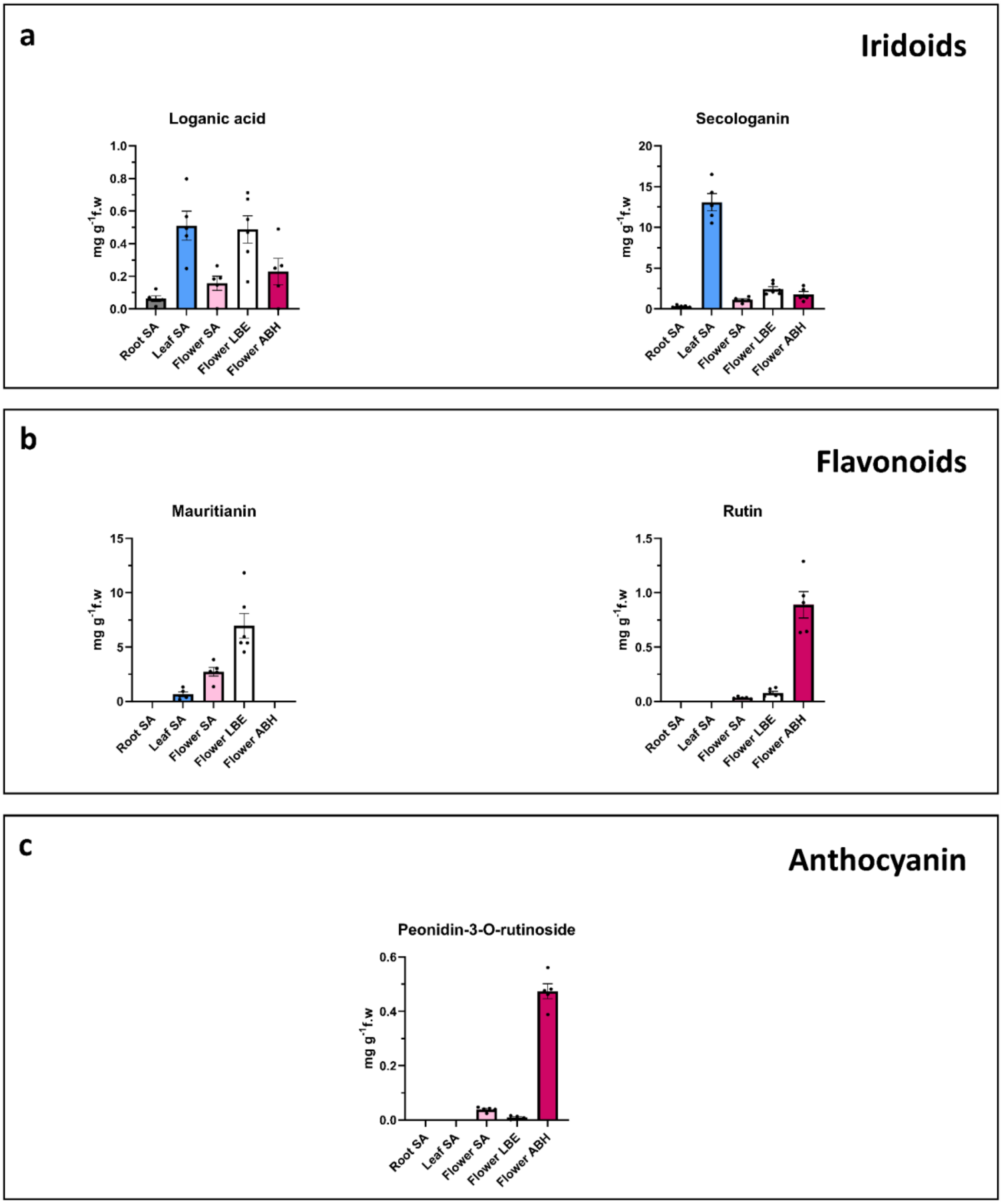

Figure S3. Quantification of key **a**, iridoids, **b**, flavonoids, and **c**, the anthocyanin peonidin-3-*O*-rutinoside in bulk tissues.

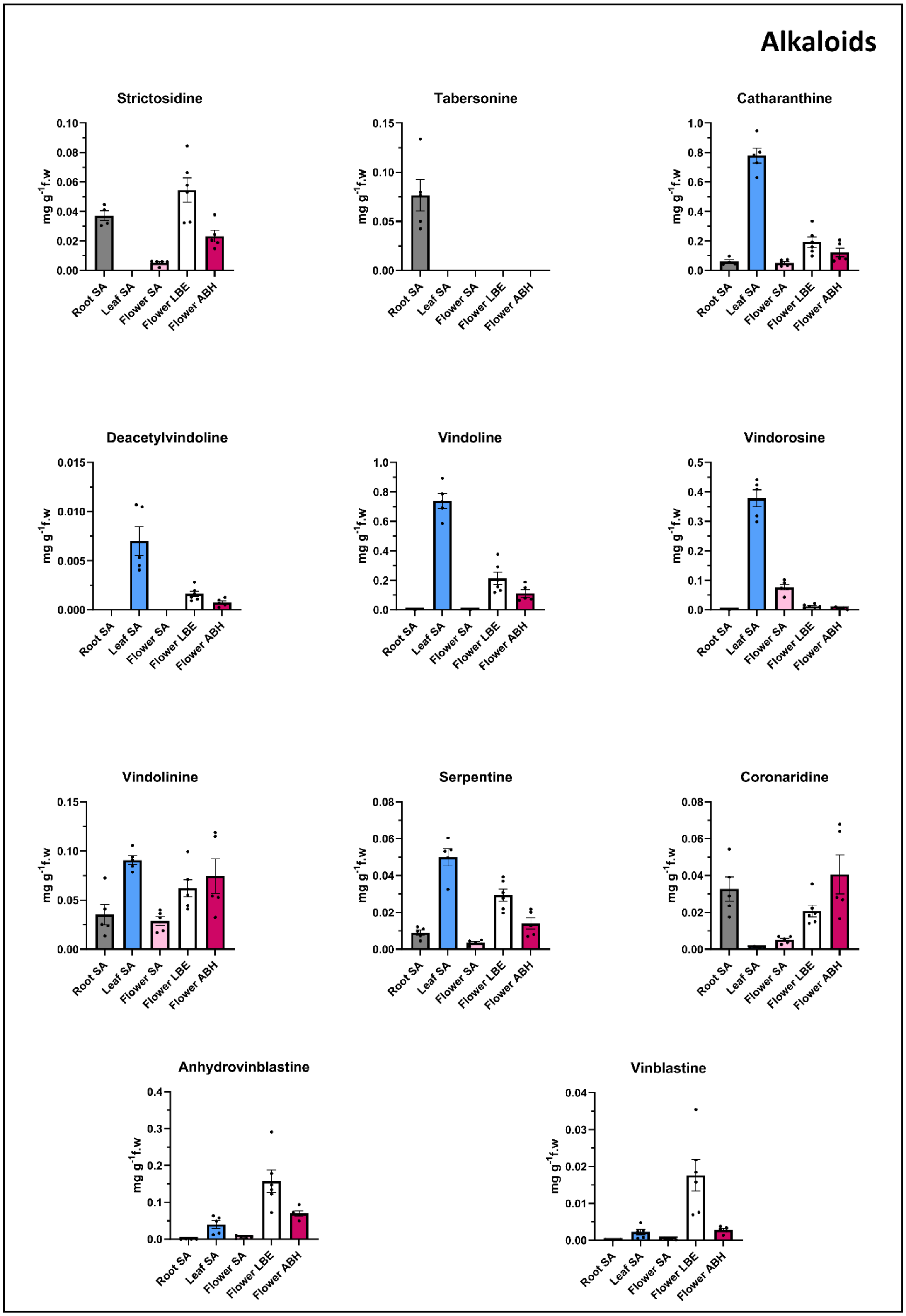

Figure S4. Quantification of alkaloids in bulk tissues.

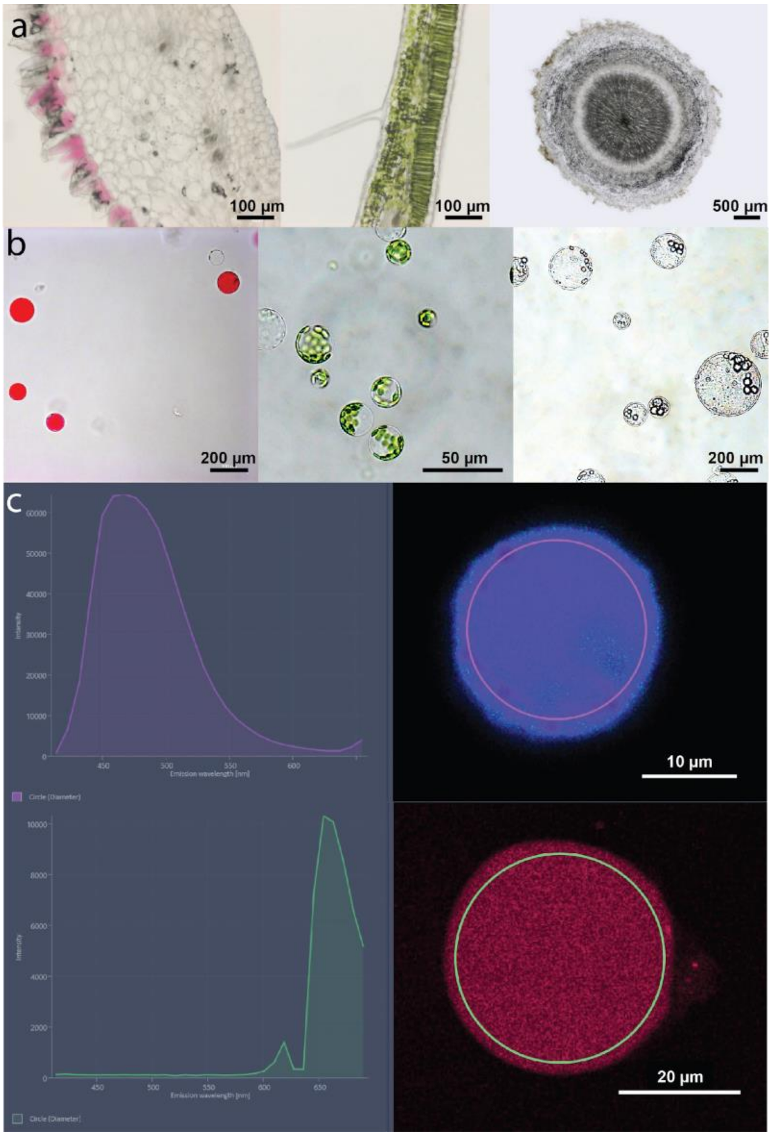

Figure S5. a, Microscopic photos of representative tissue sections (from left to right): SA petal, SA leaf, SA root. **b**, Photos of representative captured protoplasts (from left to right): ABH petal protoplasts, SA leaf protoplasts, SA root protoplasts. **c,** Photos of representative idioblast (top) and pigment (bottom) cells with their emission spectra.

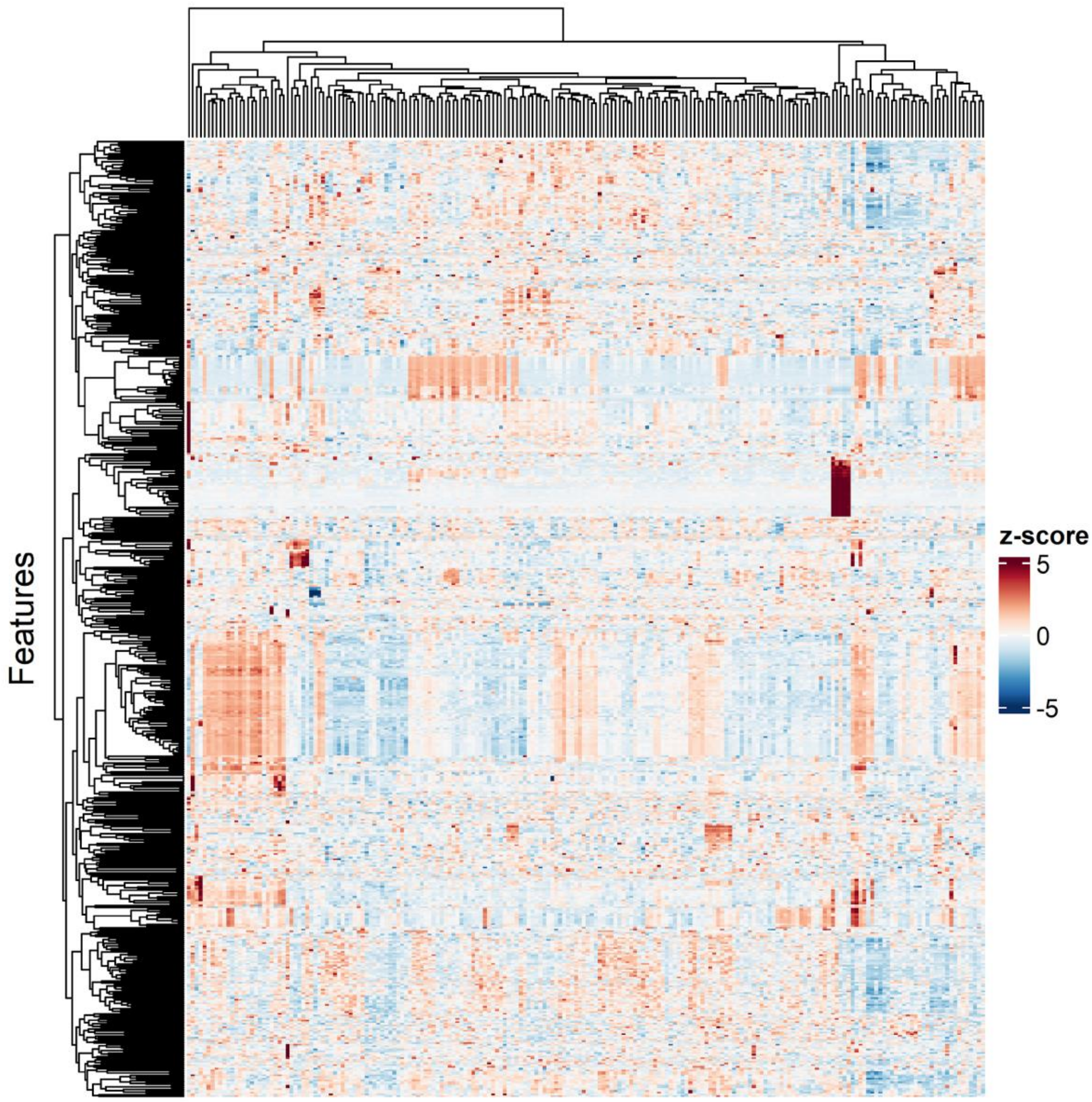

Figure S6. Heatmap representing untargeted metabolomic data of 202 leaf-derived protoplasts from the Sunstorm Apricot cultivar; 557 features were assigned a chemical formula (Table S5, Supplementary Data 3).

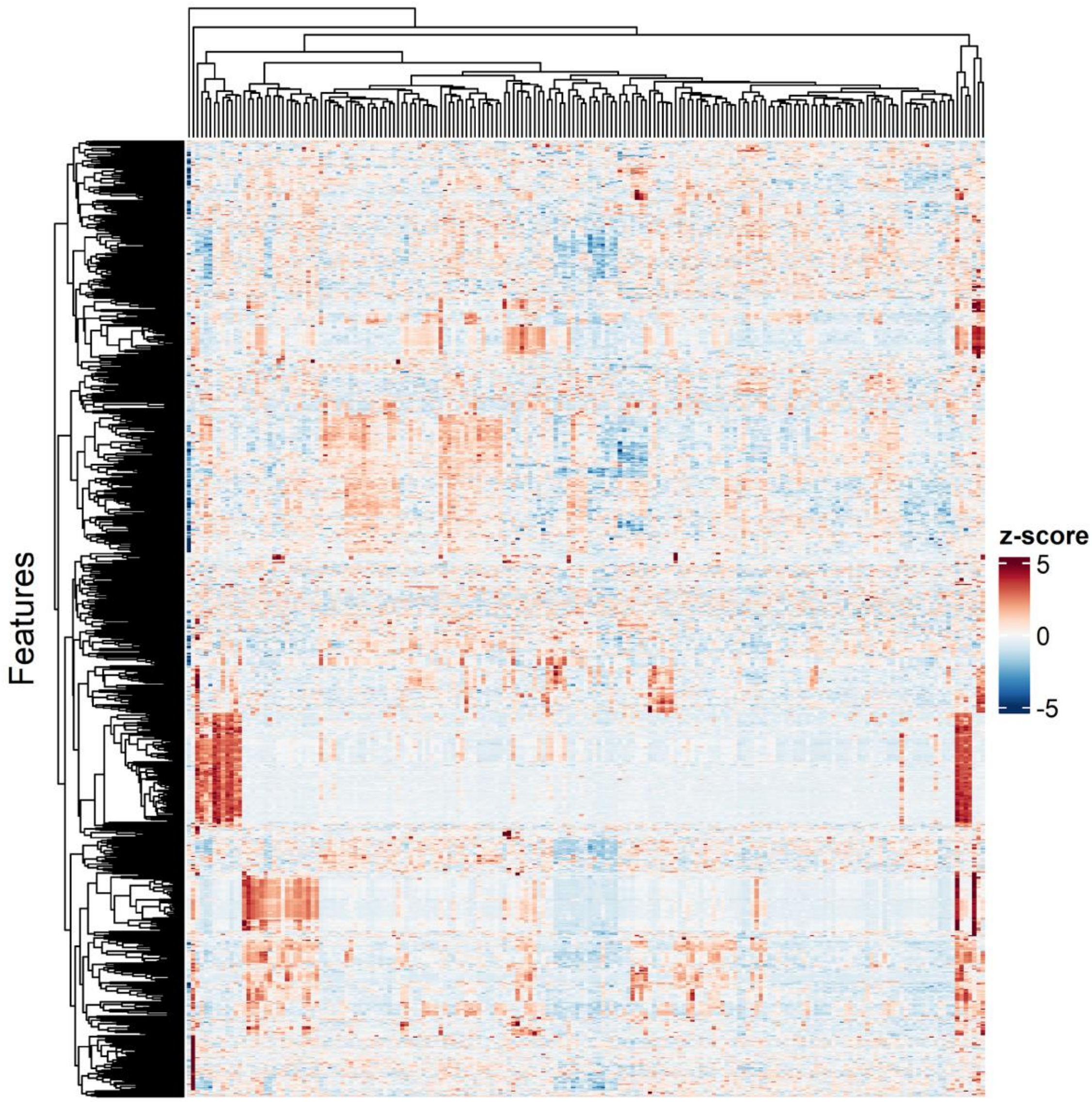

Figure S7. Heatmap representing untargeted metabolomic data of 187 root-derived protoplasts from the Sunstorm Apricot cultivar; 869 features were assigned a chemical formula (Table S5, Supplementary Data 3).

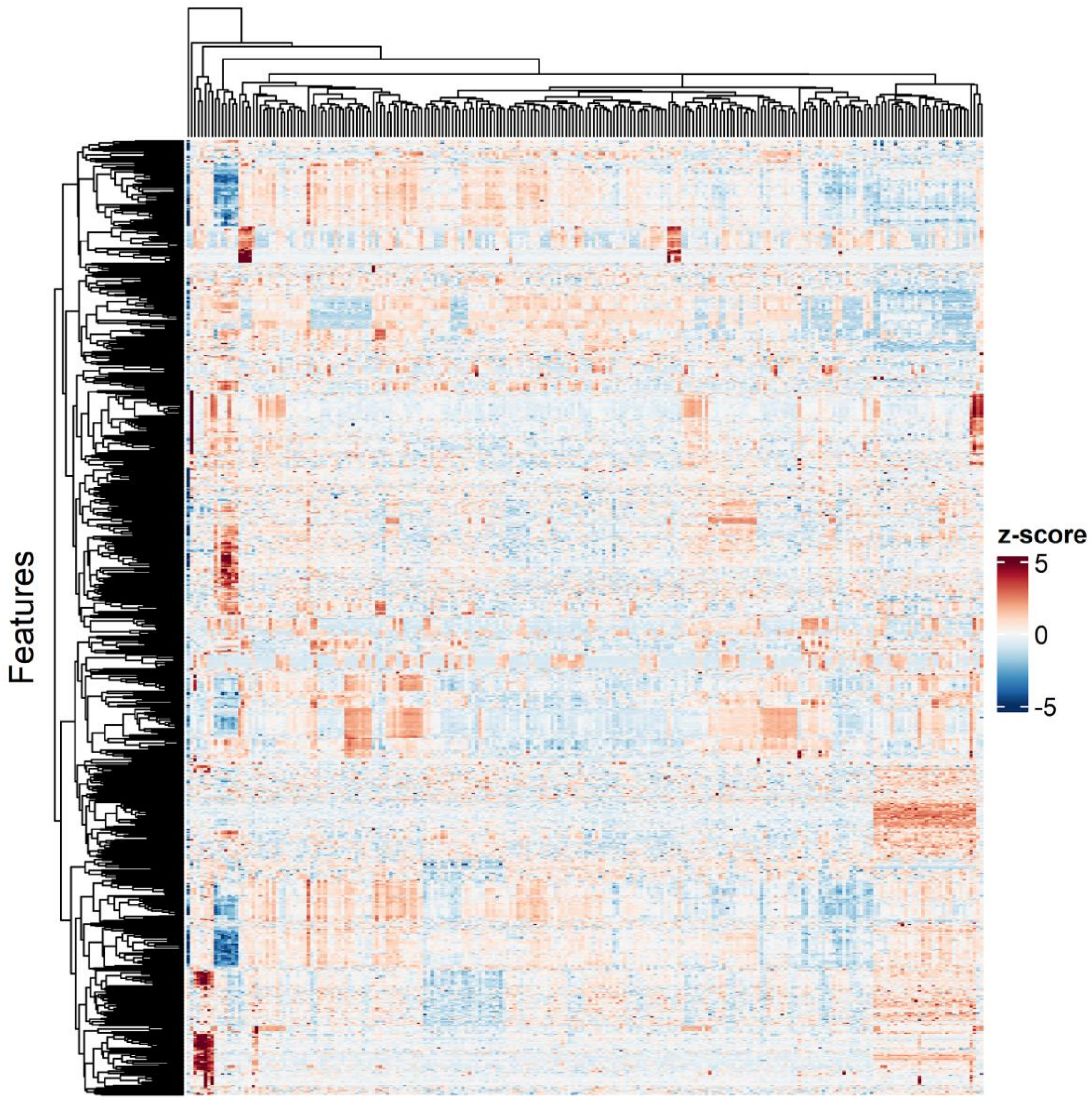

Figure S8. Heatmap representing untargeted metabolomic data of 232 petal-derived protoplasts from the Sunstorm Apricot cultivar; 765 features were assigned a chemical formula (Table S5, Supplementary Data 3).

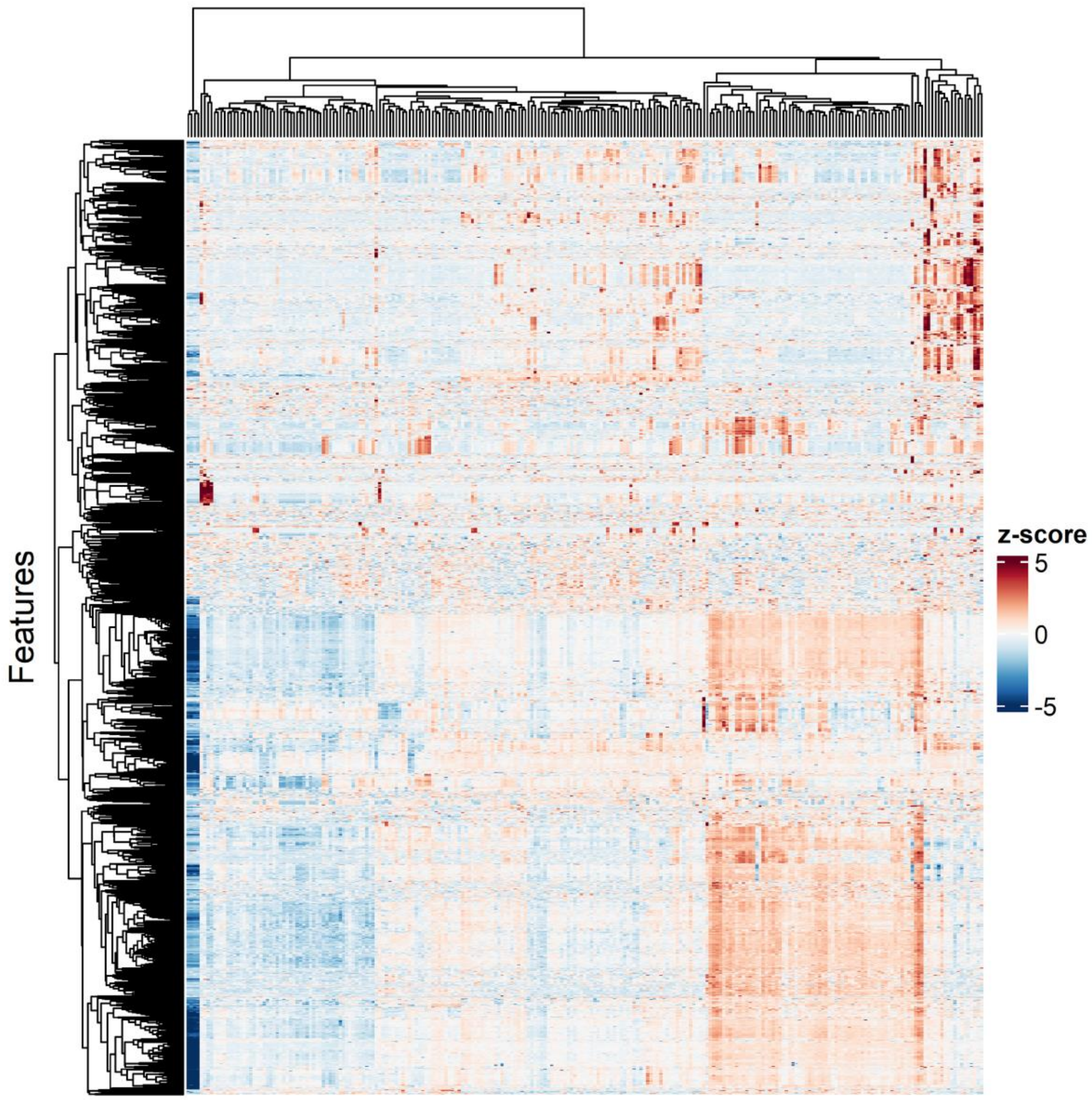

Figure S9. Heatmap representing untargeted metabolomic data of 241 petal-derived protoplasts from the Little Bright Eyes cultivar; 1373 features were assigned a chemical formula (Table S5, Supplementary Data 3).

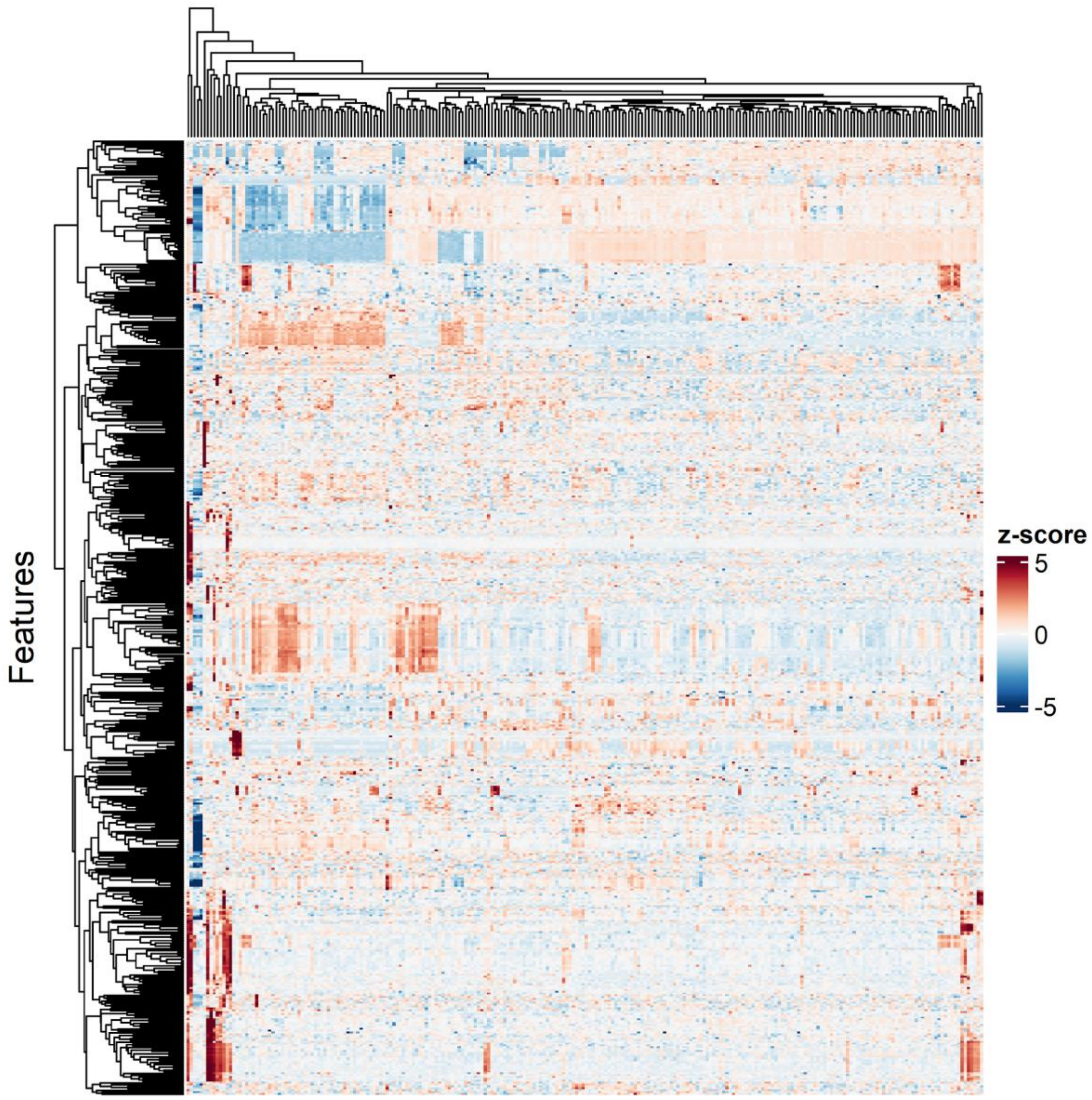

Figure S10. Heatmap representing untargeted metabolomic data of 244 petal-derived protoplasts from the Atlantic Burgundy Halo cultivar; 513 features were assigned a chemical formula (Table S5, Supplementary Data 3).

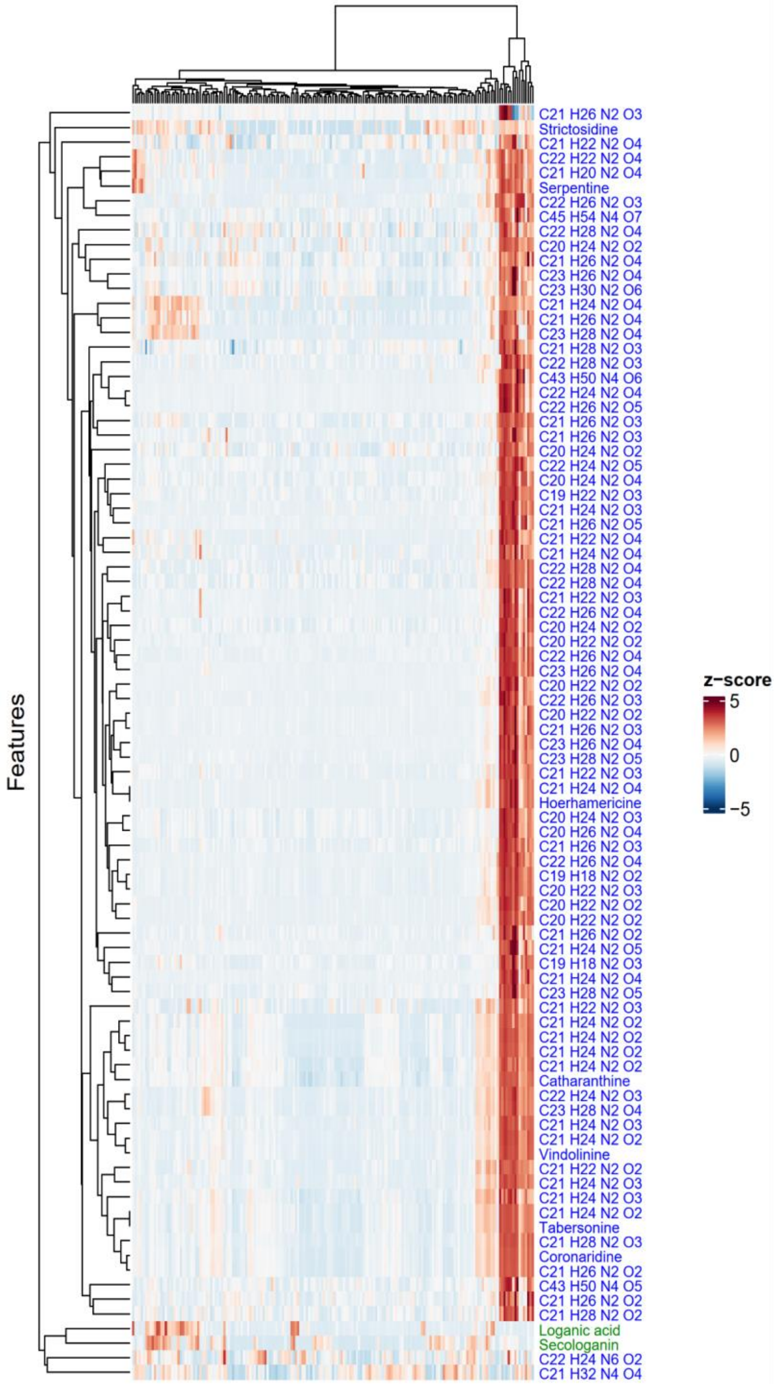

Figure S11. Heatmap showing the distribution of chemical features among the clusters. After analyzing 187 Sunstorm Apricot root protoplasts, 87 chemical features are confidently assigned to iridoid (2, in green), alkaloid (85, in blue) classes. Compounds for which a name is provided were structurally validated with an authentic standard (Figure S3) (See main text Figure 3 for comparable data for SA leaf-derived protoplasts).

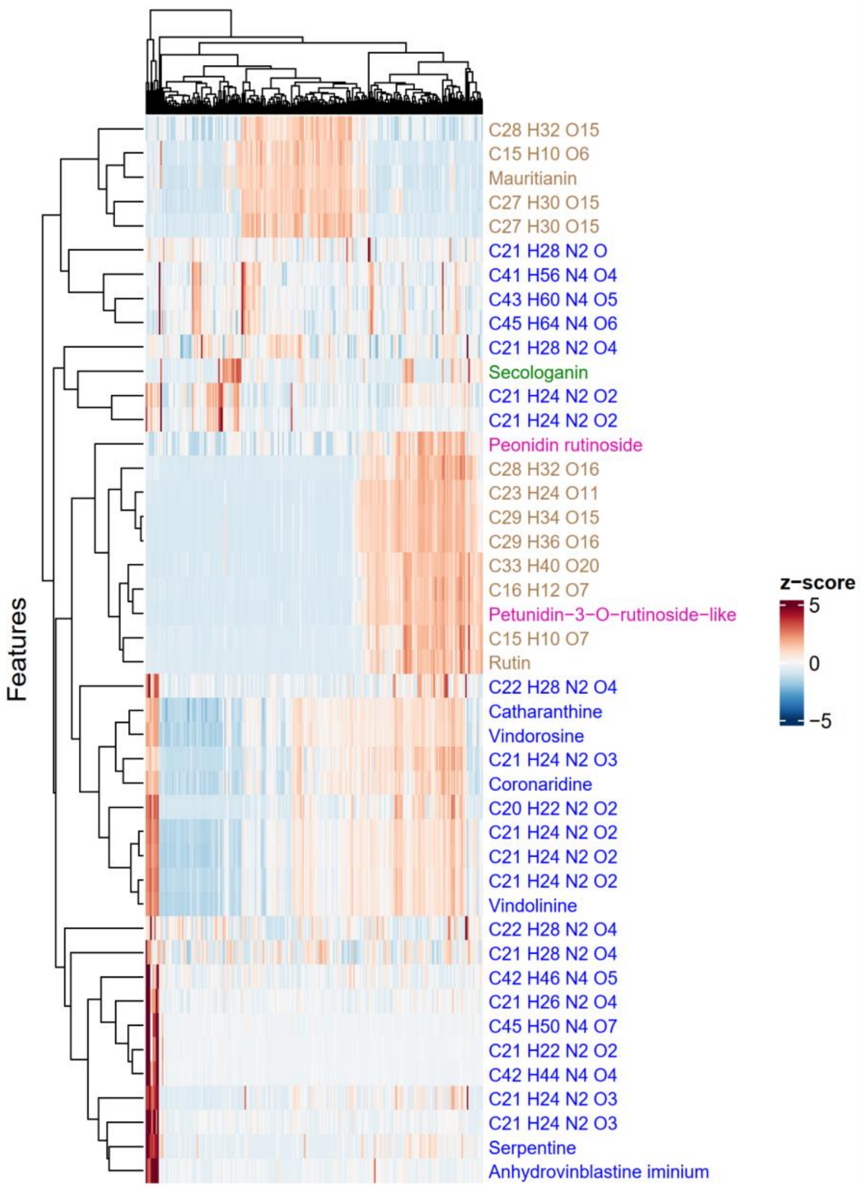

Figure S12. Heatmap showing the distribution of chemical features among the clusters. After analyzing 232 Sunstorm Apricot petal protoplasts, 44 chemical features are confidently assigned to iridoid (1, in green), alkaloid (28, in blue), flavonoid (13, in brown), or anthocyanin (2, in pink) classes. Compounds for which a name is provided were structurally validated with an authentic standard (Figure S3) (See main text Figure 3 for comparable data for SA leaf-derived protoplasts).

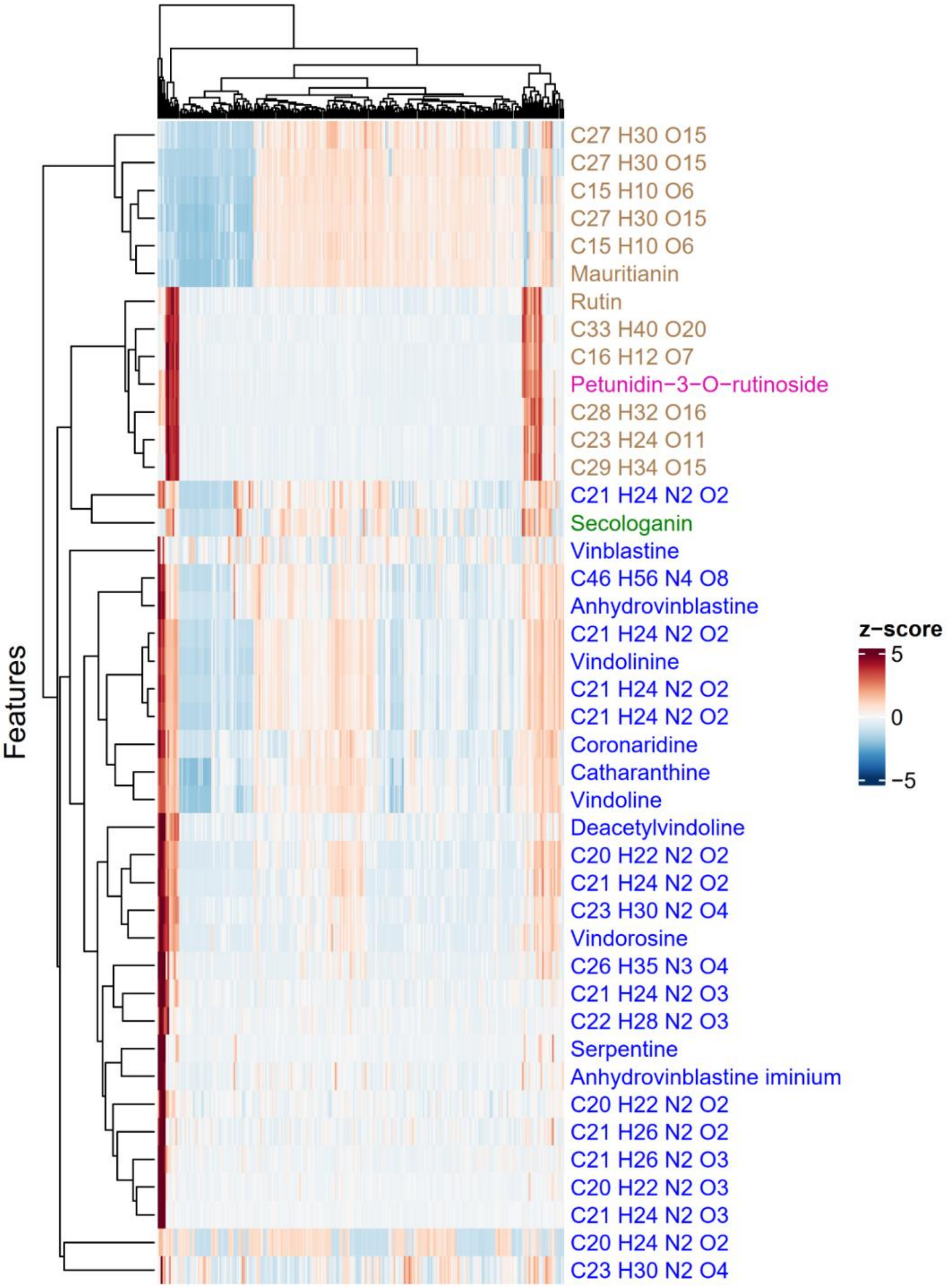

Figure S13. Heatmap showing the distribution of chemical features among the clusters. After analyzing 241 Little Bright Eyes petal protoplasts, 42 chemical features are confidently assigned to iridoid (1, in green), alkaloid (28, in blue), flavonoid (12, in brown), or anthocyanin (1, in pink) classes. Compounds for which a name is provided were structurally validated with an authentic standard (Figure S3) (See main text Figure 3 for comparable data for SA leaf-derived protoplasts).

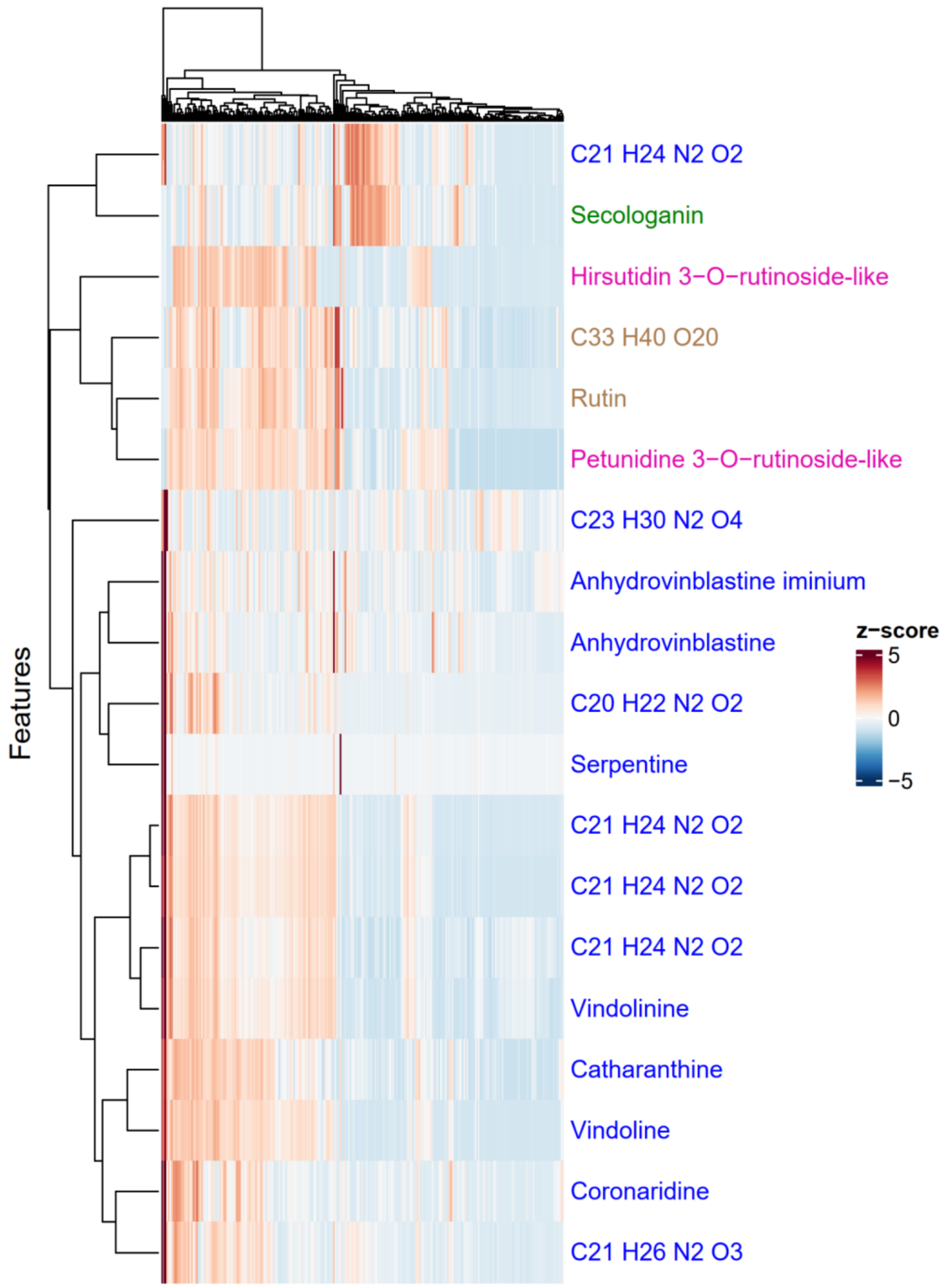

Figure S14. Heatmap showing the distribution of chemical features among the clusters. After analyzing 244 Atlantic Burgundy Halo petal protoplasts, 19 chemical features are confidently assigned to iridoid (1, in green), alkaloid (14, in blue), flavonoid (2, in brown), or anthocyanin (2, in pink) classes. Compounds for which a name is provided were structurally validated with an authentic standard (Figure S3) (See main text Figure 3 for comparable data for SA leaf-derived protoplasts).

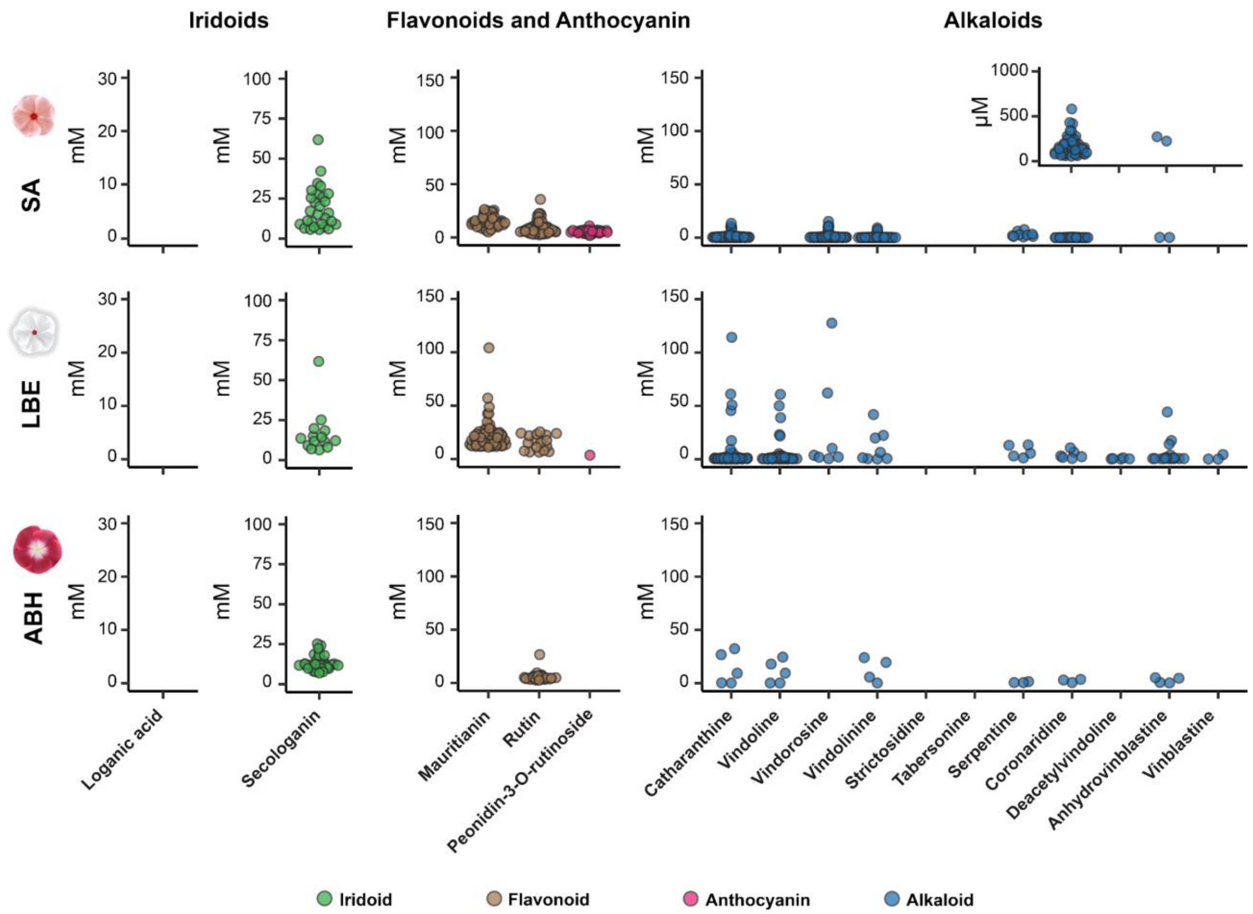

Figure S15. Quantification of iridoids, flavonoids, anthocyanins, and alkaloids in single cells isolated from flower petals from three different varieties (Supplementary Data 4).

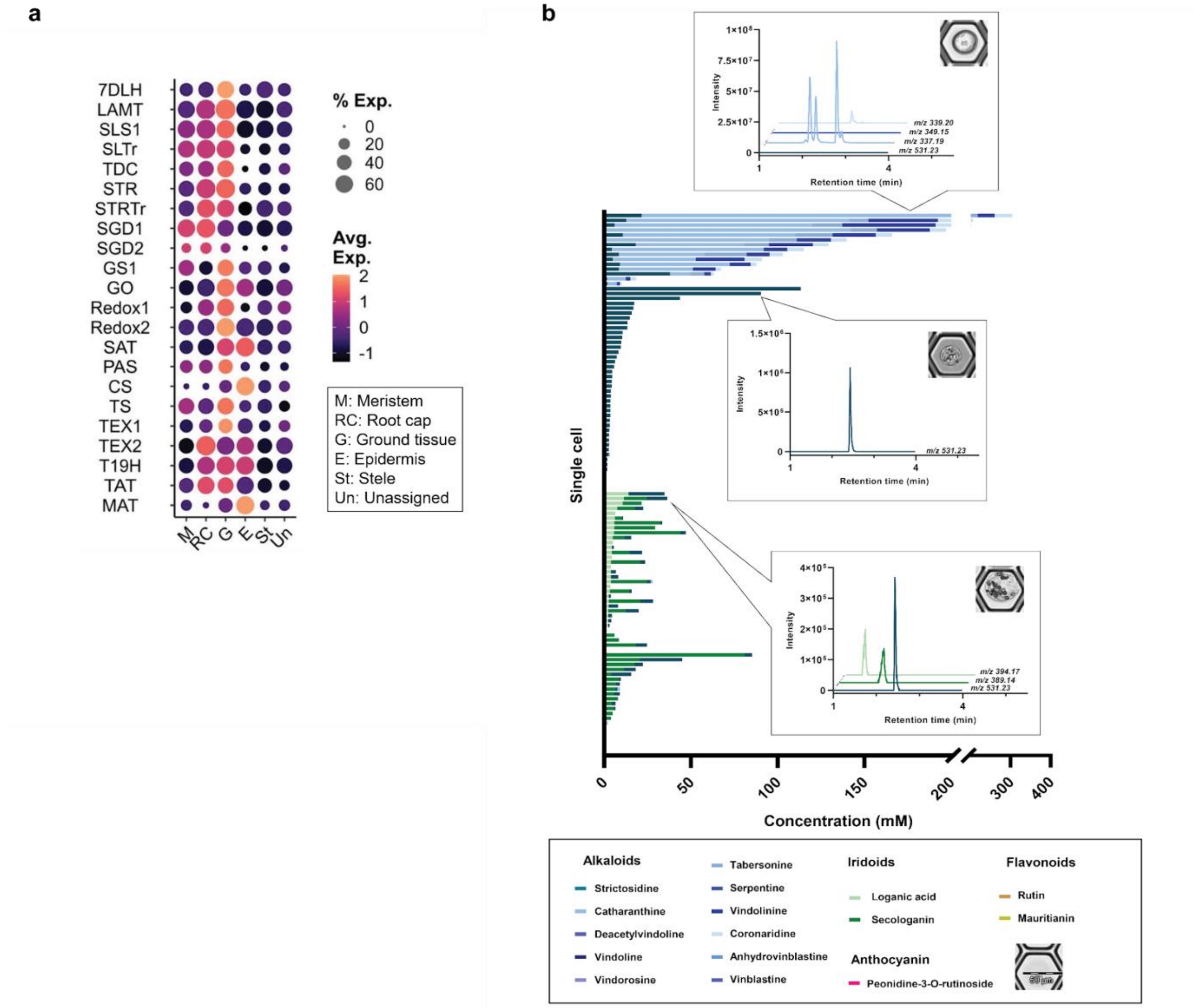

Figure S16. a, Single cell mRNA data of root protoplasts (Sunstorm Apricot cultivar). Data are taken from Li et al. 2023[6]. Dotplot shows cell-type specific expression patterns of MIA biosynthesis (See Table S1 for definition of enzyme abbreviations). **b,** Ratio of compounds found across the population of root cells that were measured (187 cells). Stack plot showing the absolute concentration of each of the quantified metabolites in each cell. Colors indicate classes of compounds: blue bars represent alkaloids, dark green represents secologain (iridoid), light green represents loganic acid (iridoid). Representative chromatograms of these compounds from individual cells. *m/z* 394.17, loganic acid; *m/z* 389.14, secologanin; *m/z* 531.23, strictosidine; *m/z* 337.19, catharanthine, and vindolinine; *m/z* 339.20, coronaridine; *m/z* 349.15, serpentine (See main text Figure 5 for comparable data for SA leaf-derived protoplasts) (Supplementary Data 4).

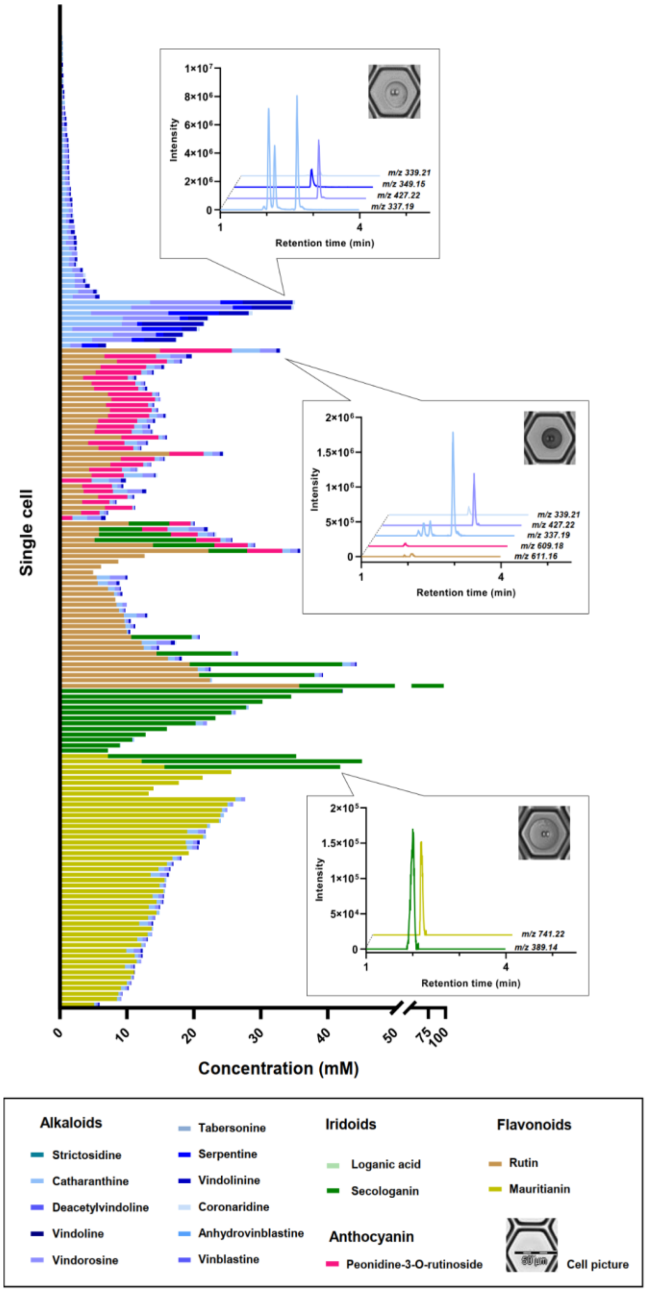

Figure S17. Ratio of compounds found across the population of petal cells (Sunstorm Apricot cultivar) that were measured (232 cells). Stack plot showing the absolute concentration of each of the quantified metabolites in each cell. Colors indicate classes of compounds: blue bars represent alkaloids, dark green represents secologain (iridoid), light green represents loganic acid (iridoid), mustard represents mauritanin (flavonoid), brown represents rutin (flavonoid), and pink represents peonidin 3-*O*-rutinoside (anthocyanin). Representative chromatograms of these compounds from individual cells. *m/z* 389.14, secologanin; *m/z* 741.22, mauritanin; *m/z* 611.16, rutin; *m/z* 609.18, peonidin 3-*O*-rutinoside; *m/z* 337.19, catharanthine, and vindolinine; *m/z* 339.21, coronaridine; *m/z* 427.22, vindorosine; *m/z* 349.15, serpentine (See main text Figure 5 for comparable data for SA leaf-derived protoplasts) (Supplementary Data 4).

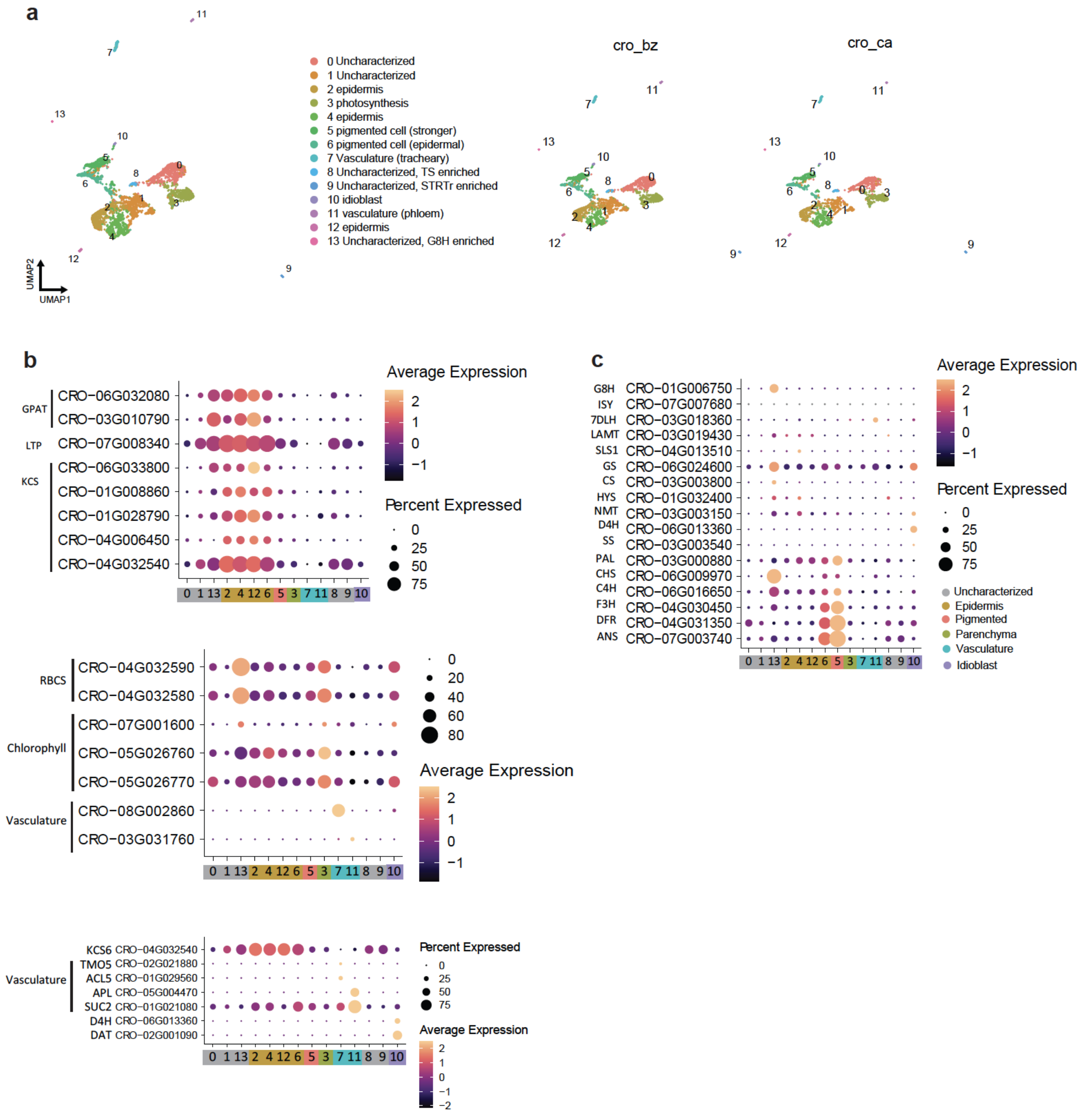

Figure S18. Single cell mRNA data of Sunstorm Apricot (SA) petals. **a**, UMAP plot of scRNA-Seq for SA petals. **b**, Dotplots for marker genes. Cuticle biosynthesis genes (GPAT, LTP, KCS), chloroplast-related genes (RBCS, Chlorophyll binding proteins), vasculature-related genes were identified as de novo marker genes for the clusters. Marker genes previously used in leaf datasets (Li et al. 2023[6]) also confirmed the cell annotations. **c,** Dotplot shows cell-type specific expression patterns of MIA biosynthetic enzymes (See Table S1 for definition of enzyme abbreviations) (Supplementary Data 5).

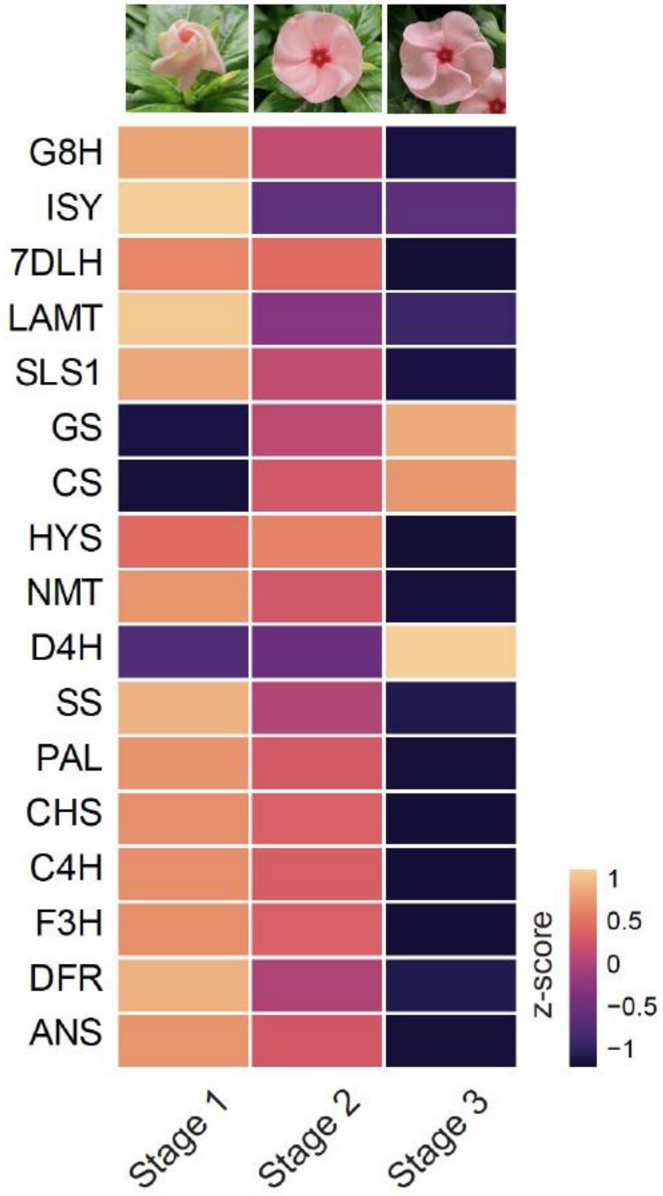

Figure S19. Bulk RNAseq data at a variety of time points immediately after Sunstorm Apricot flower opening (See Table S1 for definition of enzyme abbreviations) (Supplementary Data 6).

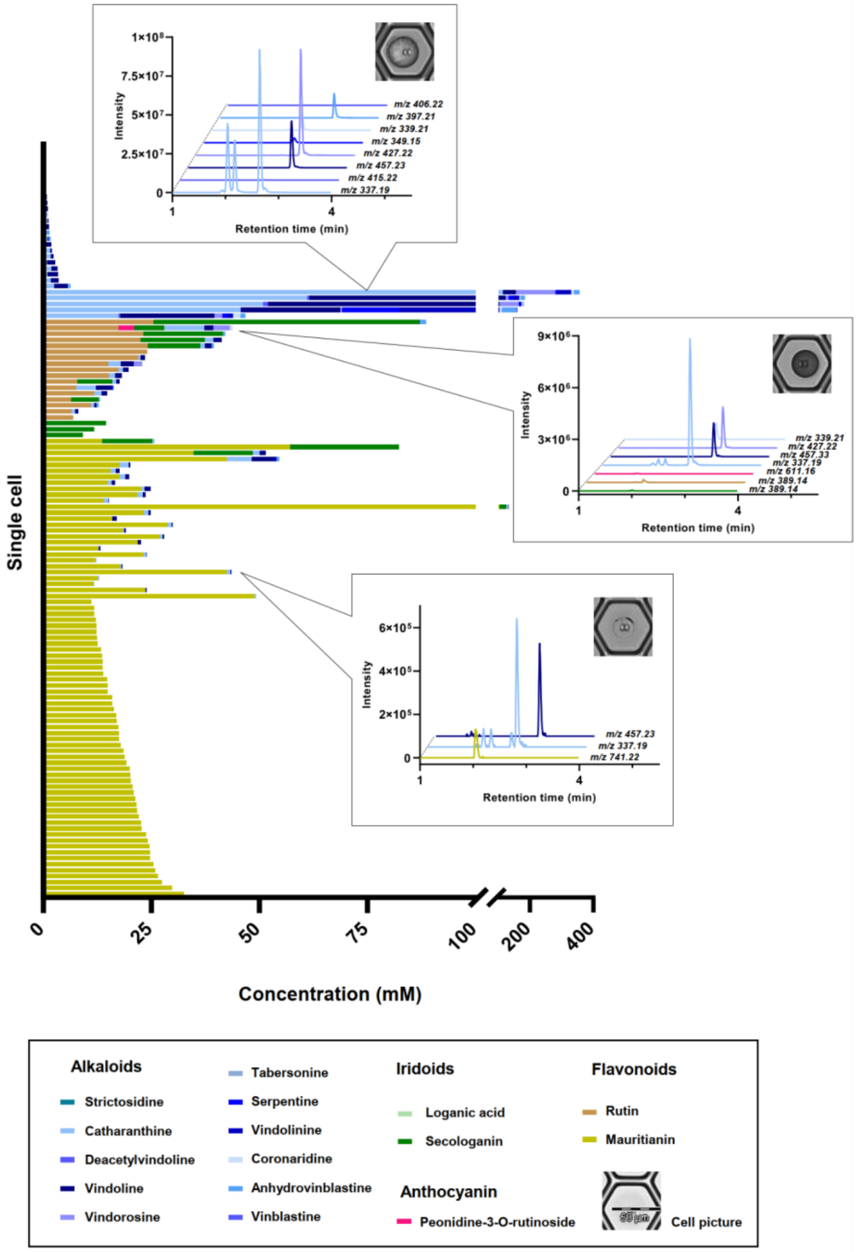

Figure S20. Ratio of compounds found across the population of petal cells (Little Bright Eyes cultivar) that were measured (241 cells). Stack plot showing the absolute concentration of each of the quantified metabolites in each cell. Colors indicate classes of compounds: blue bars represent alkaloids, dark green represents secologain (iridoid), mustard represents mauritanin (flavonoid), brown represents rutin (flavonoid), and pink represents peonidin 3-*O*-rutinoside (anthocyanin). Representative chromatograms of these compounds from individual cells. *m/z* 389.14, secologanin; *m/z* 389.14, rutin; *m/z* 741.22, mauritanin; *m/z* 611.16, peonidin-3-*O*-rutinoside; *m/z* 337.19, catharanthine, and vindolinine; *m/z* 457.23, vindoline; *m/z* 427.22, vindorosine; *m/z* 349.15, serpentine; *m/z* 415.22, deacetylvindoline; *m/z* 339.21, coronaridine; *m/z* 397.21, anhydrovinblastine; *m/z* 406.22, vinblastine (See main text Figure 5 for comparable data for SA leaf-derived protoplasts) (Supplementary Data 4).

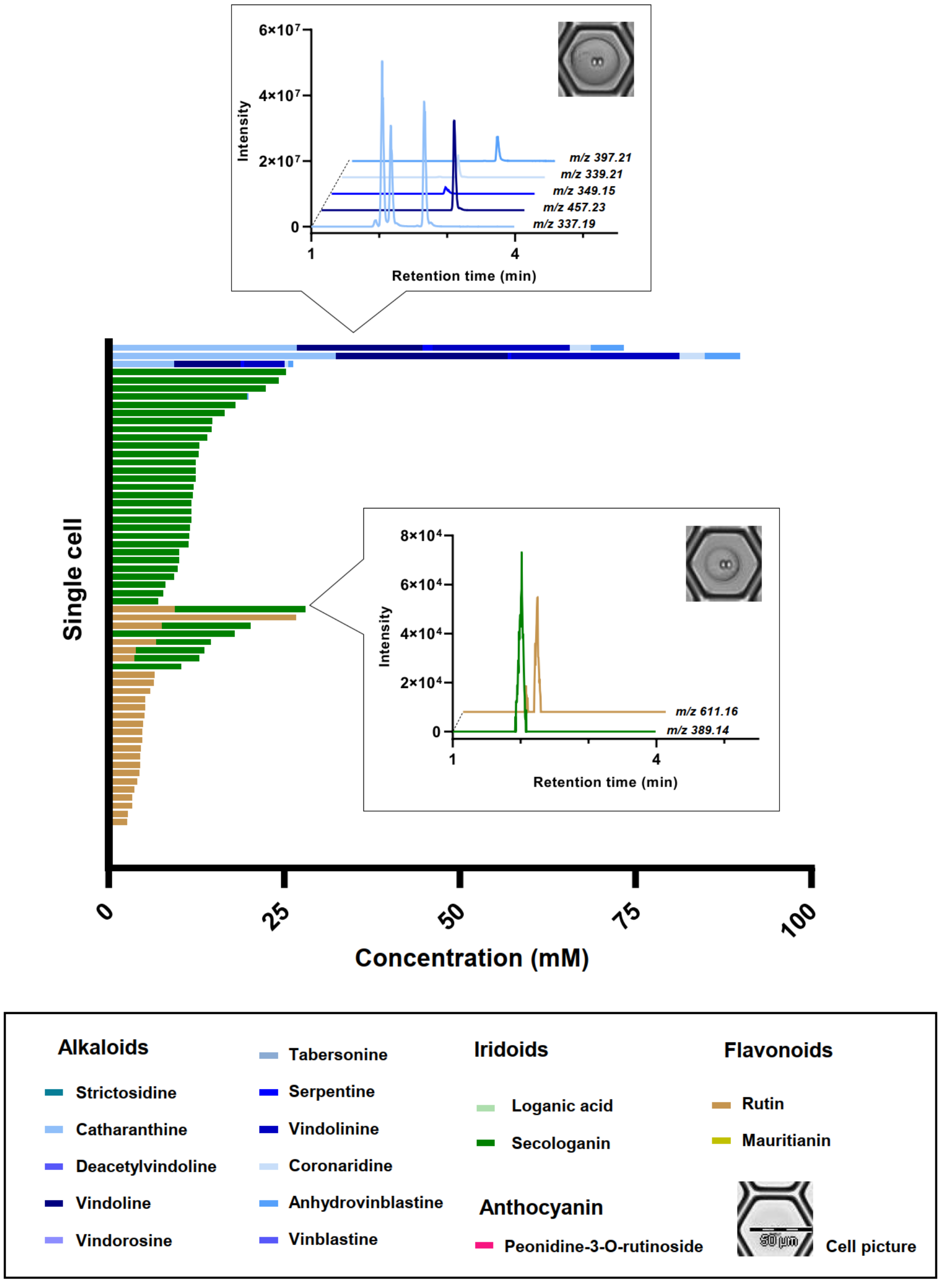

Figure S21. Ratio of compounds found across the population of petal cells (Atlantic Burgundy Halo cultivar) that were measured (244 cells). Stack plot showing the absolute concentration of each of the quantified metabolites in each cell. Colors indicate classes of compounds: blue bars represent alkaloids, dark green represents secologain (iridoid), light green represents loganic acid (iridoid), mustard represents mauritanin (flavonoid), brown represents rutin (flavonoid), and pink represents penodine-3-*O*-rutinoside (anthocyanin). Representative chromatograms of these compounds from individual cells. *m/z* 389.14, secologanin; *m/z* 611.16, rutin; *m/z* 337.19, catharanthine, and vindolinine; *m/z* 457.23, vindoline; *m/z* 349.15, serpentine; *m/z* 397.21, anhydrovinblastine (See main text Figure 5 for comparable data for SA leaf-derived protoplasts) (Supplementary Data 4).

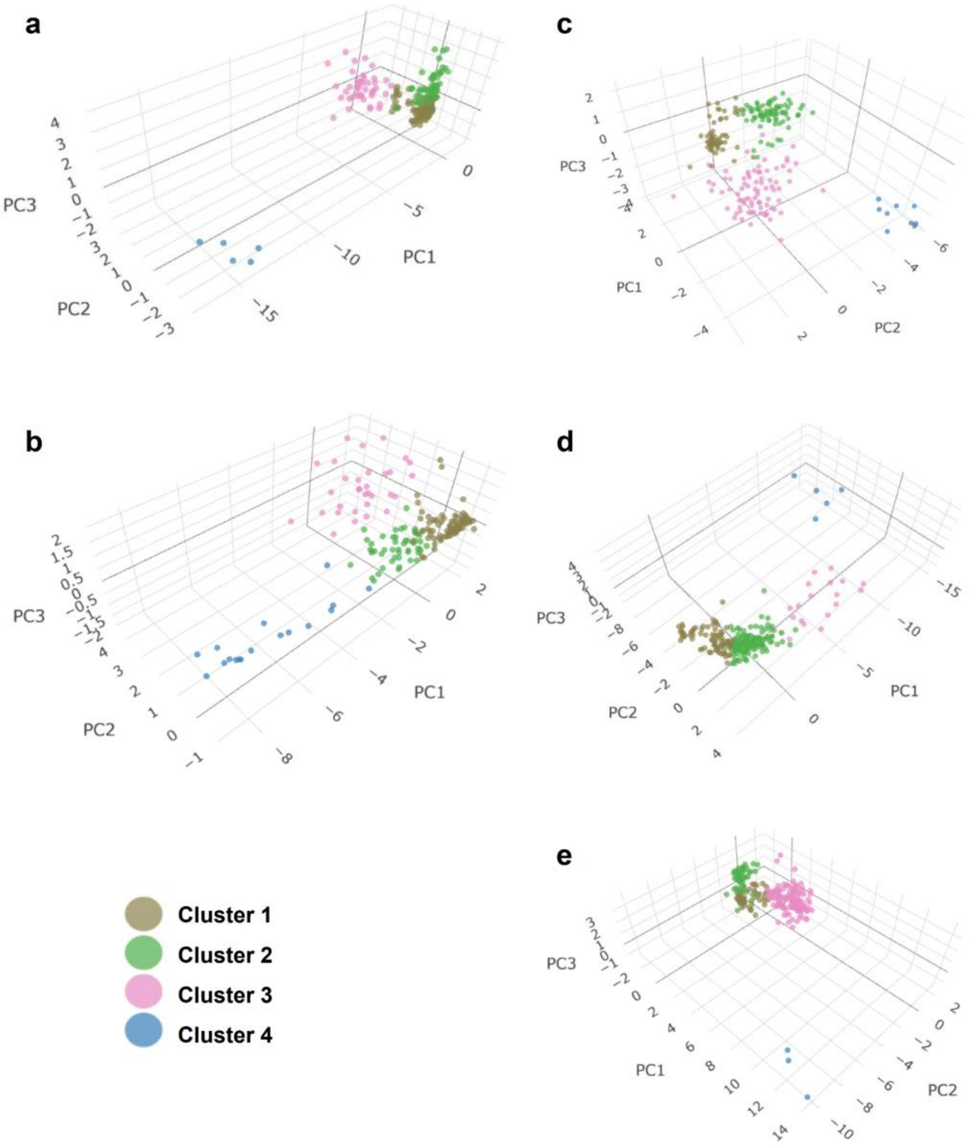

Figure S22. PCA of single cells using only the compounds identified by co-elution with a reference standard from 5 different tissues: a. Sunstorm Apricot leaf, **b,** Sunstorm Apricot root, **c**, Sunstorm Apricot petal, **d**, Little Bright Eyes petal, **e,** Atlantic Burgundy Halo petal. Using k-means clustering analysis, the optimal number of clusters was defined as 4 using the Elbow method[4]. Using these parameters, cells were classified into four well-defined and distinct clusters.

Supplementary Tables

Table S1. Definition of enzyme abbreviations.

Abbreviation	Enzyme
7DLH	7-deoxyloganic acid 7-hydroxylase
ANS	Anthocyanidin synthase
C4H	Cinnamate 4-hydroxylase
CHS	Chalcone synthase
CS	Catharanthine synthase
D4H	Desacetoxyvindoline-4-hydroxylase
DFR	Dihydroflavonol 4-reductase
F3H	Flavanone–3-hydroxylase
G8H	Geraniol 8-hydroxylase
GO	Geissoschizine oxidase
GPAT	Glycerol-3-phosphate O-acyltransferase
GS	Geissoschizine synthase
HYS	Heteroyohimbine synthase
ISY	Iridoid synthase
KCS	β-Ketoacyl-CoA synthase
LAMT	Loganic acid methyltransferase
LTP	Lipid transfer protein
MAT	Minovincinine 19-hydroxy-O-acetyltransferase enzymes
NMT	16-hydroxy-2,3-dihydro-3-hydroxytabersonine N-methyltransferase
PAL	Phenylalanine ammonia lyase
PAS	Precondylocarpine acetate synthase
RBCS	Ribulose bisphosphate carboxylase (small chain)
Redox	Reductive enzyme
SAT	Stemmadenine acetyl transferase
SGD	Strictosidine β-glucosidase
SLS	Secologanin synthase
SLTr	Secologanin transporter
SS	Serpentine synthase
STR	Strictosidine synthase
STRTr	Strictosidine transporter
T19H	Tabersonine/lochnericine 19-hydroxylase
TAT	Tabersonine derivative 19-O-acetyltransferase
TDC	Tryptophan decarboxylase
TEX	Tabersonine 6,7-epoxidase
TS	Tabersonine synthase

Table S2. Validation of the reproducibility of the LC-MS method. Results are presented as RSD% of the peak area and retention time; d=dilution factor.

Tissue	Compounds	Intra-day repeatability (%RSD)		Inter-day repeatability (3 consecutive days) (%RSD)	
		Peak area (n=10)	Retention time (n=10)	Peak area (n=3)	Retention time (n=3)
Petal d=50	Mauritianin	1.40	0.50	1.58	0.45
	Rutin	1.95	0.42	3.26	0.58
	Catharanthine	1.28	0.49	4.17	0.46
	Serpentine	1.83	0.54	4.92	0.50
	Coronaridine	1.06	0.52	3.63	0.46
	Vindorosine	0.97	0.40	3.57	0.36
	Internal standard	1.87	0.29	3.68	0.36
Leaf d=500	Mauritianin	3.54	0.40	4.19	0.42
	Catharanthine	1.82	0.30	3.75	0.40
	Serpentine	2.04	0.38	2.54	0.44
	Coronaridine	3.48	0.37	3.56	0.39
	Vindoline	0.96	0.15	1.46	0.25
	Vindorosine	1.76	0.12	3.75	0.24
	Internal standard	2.53	0.32	3.45	0.35
Root d=200	Catharanthine	0.97	0.38	2.89	0.39
	Serpentine	4.52	0.43	4.70	0.43
	Coronaridine	0.95	0.35	2.84	0.38
	Tabersonine	1.19	0.33	2.78	0.32
	Internal standard	2.17	0.26	2.88	0.31

Table S3. Compounds used for identification.

Compound class	Compound name	Chemical formula	Ion	Measured m/z	Δppm	Retention time (min)
Iridoids	Loganic acid	$C_{16}H_{24}O_{10}$	$[M+NH_4]^+$	394.17062	0.38	1.48
	Secologanin	$C_{17}H_{24}O_{10}$	$[M+H]^+$	389.14429	0.18	2.02
Flavonoids	Mauritianin	$C_{33}H_{40}O_{19}$	$[M+H]^+$	741.22357	0.12	2.09
	Rutin	$C_{27}H_{30}O_{16}$	$[M+H]^+$	611.16064	0.03	2.08
Anthocyanins	Peonidin 3-*O*-rutinoside	$C_{28}H_{33}O_{15}$	$[M]^+$	609.18164	0.39	1.80
	Petunidin 3-*O*-rutinoside*	$C_{28}H_{33}O_{16}$	$[M]^+$	625.17554	1.23	2.30
	Hirsutidin 3-*O*-rutinoside*	$C_{30}H_{37}O_{16}$	$[M]^+$	653.20673	1.35	2.04
Alkaloids	Strictosidine**	$C_{27}H_{34}N_2O_9$	$[M+H]^+$	531.23383	0.24	2.43
	Catharanthine	$C_{21}H_{24}N_2O_2$	$[M+H]^+$	337.19064	1.22	2.65
	Deacetylvindoline	$C_{23}H_{30}N_2O_5$	$[M+H]^+$	415.22247	0.67	2.58
	Vindoline	$C_{25}H_{32}N_2O_6$	$[M+H]^+$	457.23325	0.13	2.97
	Tabersonine	$C_{21}H_{24}N_2O_2$	$[M+H]^+$	337.19080	0.74	2.77
	Hörhammericine**	$C_{21}H_{24}N_2O_4$	$[M+H]^+$	369.18051	0.79	1.77
	Serpentine	$C_{21}H_{20}N_2O_3$	$[M]^+$	349.15448	0.54	2.65
	Vindolinine	$C_{21}H_{24}N_2O_2$	$[M+H]^+$	337.19077	0.83	2.19
	Coronaridine**	$C_{21}H_{26}N_2O_2$	$[M+H]^+$	339.20657	0.38	2.70
	Vindorosine**	$C_{24}H_{30}N_2O_5$	$[M+H]^+$	427.22241	0.80	2.99
	Anhydrovinblastine iminium**	$C_{46}H_{54}N_4O_8$	$[M+2H]^{2+}$	396.20432	0.10	3.00
	Anhydrovinblastine	$C_{46}H_{56}N_4O_8$	$[M+2H]^{2+}$	397.21222	0.10	3.16
	Vinblastine	$C_{46}H_{58}N_4O_9$	$[M+2H]^{2+}$	406.21741	0.15	2.94

*Putative assignment based on mass and fragmentation of other glucosides with the same aglycon, standard is not available.
** Standards were synthesized/isolated in our laboratory.

Table S4. Analytical parameters of the compounds quantified in this study.

Compound	Calibration range (nM)	Regression equation[a]	Correlation Coefficient (R^2)	LOQ[b] (nM)
Loganic acid	3.5-9000	y=16211x + 242751	0.9999	3.5
Secologanin	8.0-3000	y = 13483x + 429913	0.9987	8
Mauritianin	10.0-3000	y = 13958x + 42965	0.9998	10
Rutin	3.5-8000	y=18915x + 298272	0.9998	3.5
Peonidin 3-*O*-rutinoside	5-900	y=14348x + 35023	0.9997	5
Strictosidine	1.0-500	y = 27175x + 11179	0.9998	1
Catharanthine	0.1-200	y = 1362842x + 856727	0.9997	0.1
Deacetylvindoline	0.1-100	y = 1076941x + 12409	1	0.1
Vindoline	0.1-300	y = 1341203x + 707158	0.9995	0.1
Vindorosine	0.2-450	y= 859082x + 1547496	0.9995	0.2
Tabersonine	0.1-250	y = 928020x + 48163	0.9997	0.1
Serpentine	0.12-600	y = 1382354x + 1665432	0.9993	0.12
Vindolinine	0.1-245	y=1438007x + 1085239	0.9997	0.1
Coronaridine	0.1-200	y = 1918865x + 663204	0.9999	0.1
Anhydrovinblastine	0.15-150	y = 1509391x + 273636	0.9996	0.15
Vinblastine	0.06-100	y = 1207682x + 444112	0.9991	0.06

[a]Each point of calibration curve was measured in triplicate.
[b]The LOQ was estimated in the lowest analyte concentration injected that yielded a signal-to-noise (S/N) ratio of ≥10 in three replicates.

Table S5. Summary of parameters of scMS for 5 tissues.

Tissue of origin	No. of cells analyzed	No. of features with formula	No. of features with MS^2 data	No. of features/cell[a,b]
Leaf	202	557	295	17-119
Root	187	869	438	10-280
Petal SA	232	765	321	27-106
Petal LBE	241	1373	567	72-221
Petal ABH	244	513	239	28-140

[a]No. of features with formula assignment with error lower than 2 ppm and after blank filtering.
[b]10 cells/dataset were randomly selected for this calculation.

Table S6. List of all chemicals used in this study.

No.	Compound	CAS	Supplier	SKU/Code
**Alkaloids**				
1	Catharanthine	2468-21-5	Sigma Aldrich	SML0259
2	Tabersonine	4429-63-4	Sigma Aldrich	SMB00452
3	Vindoline	2182-14-1	Acros Organics BVBA	462270010
4	Anhydrovinblastine disulfate	81165-17-5	TRC Canada	TRC-A659750
5	Vinblastine sulfate	143-67-9	Acros Organics BVBA	203450050
6	Serpentine hydrogen tartrate	58782-36-8	Sequoia Research Products Ltd	SRP01300s
7	Strictosidine (10 mM)	20824-29-7	Synthesized	
8	Ajmaline	4360-12-7	Extrasynthese	0623
9	Ajmalicine	483-04-5	Sigma Aldrich	41111
10	Coronaridine	467-77-6	Isolated	
11	Deacetylvindoline	3633-92-9	TRC Canada	D288745
12	Vindolinine	5980-02-9	Advanced ChemBlocks Inc	U105985
13	Hörhammericine		Synthesized	
14	Vindorosine	5231-60-7	Isolated	
**Iridoids**				
1	Secologanin	19351-63-4	Sigma Aldrich (SAFC)	50741
2	Loganic acid	22255-40-9	Extrasynthese	0230 S
**Anthocyanins**				
1	Petunidin-3-*O*-glucoside chloride	6988-81-4	Extrasynthese	0919
2	Peonidin-3-*O*-glucoside chloride	6906-39-4	Extrasynthese	0929 S
3	Peonidin-3-*O*-rutinoside chloride	27539-32-8	Extrasynthese	0945
4	Hirsutidin chloride	4092-66-4	TRC Canada	TRC-H356800
5	Peonidin chloride	134-01-0	Extrasynthese	0906 S
**Flavonoids**				
1	Rutin trihydrate	250249-75-3	Sigma Aldrich	78095
2	Mauritianin	109008-28-8	BIOMOL GmbH	34326
**Protoplast isolation**				
1	Macerozyme R-10 from Rhizopus sp. Lyophil.	9032-75-1	SERVA	28302.02

2	Cellulase »Onozuka« R-10 aus Trichoderma viride ca. 1 U/mg	9012-54-8	SERVA	16419.03
3	Pectinase from Aspergillus niger	9032-75-1	Sigma Aldrich	P4716
4	Fluorescein Diacetate	596-09-8	TCI	F0240
5	D-Mannitol	69-65-8	Sigma Aldrich	6356
6	MES hydrate	1266615-59-1	Sigma Aldrich	M8250
7	Bovine serum albumin	9048-46-8	Sigma Aldrich	A2153
**Sample preparation and mobile phases**				
1	Formic Acid Optima LC/MS	64-18-6	Fisher Scientific	A117
2	Acetonitrile OPTIMA® LC/MS GRADE	75-05-8	Fisher Scientific	A955-212
3	Methanol UHPLC-MS	67-56-1	Fisher Scientific	A458-1

Table S7. Compound Discoverer™ important parameters.

**Nodes**	**Parameters**		
Select Spectra	Spectrum Properties Filter	Lower RT Limit	0
		Upper RT Limit	0
	Scan Event Filters	Polarity Mode	Is +
Align Retention Times	General Settings	Alignment Model	Adaptive curve
		Maximum Shift (min)	0.5
		Mass Tolerance	5 ppm
Detect Compounds	Mass Tolerance	Mass Tolerance	5 ppm
		Intensity Tolerance	30
		Min Peak Intensity	10000
Group Compounds	Compound Consolidation	Mass Tolerance	5 ppm
		RT Tolerance	0.2 min
Fill Gaps	General Settings	Mass Tolerance	5 ppm
		S/N Threshold	1.5
Apply QC Correction	General Settings	Min. QC Coverage (%)	25
		Max. QC Area RSD (%)	80
		Max. Corrected QC Area RSD (%)	50
Mark Background Compounds	General Settings	Max. Sample/Blank	5
		Max. Blank/Sample	0
		Hide Background	True
Assign Compound Annotations	General Settings	Mass Tolerance	2 ppm
	Data Sources	Data Source#1	Predicted Compositions
	Scoring Rules	Use mzLogic	True
		Use Spectral Distance	True
		SFit Threshold	20
		SFit Range	20
Predict Compositions	Prediction Settings	Mass Tolerance	2 ppm
		Min. RDBE	0
		Max. RDBE	40
		Min. H/C	0.1
		Max. H/C	4
		Max. #Candidates	10
	Pattern Matching	Intensity Tolerance(%)	30
		Intensity Threshold (%)	0.1
		S/N Threshold	3
		Use Dynamic Recalibration	True
	Fragments matching	Use Fragments Matching	True
		Mass Tolerance	5 ppm
		S/N Threshold	3

Supplementary MS^2 Spectra

Fragmentation data from all compounds identified in this study. Empty panels indicate that it was not possible to acquire MS^2 data from that particular sample, either because the compound was not present or because the signal was insufficient to trigger the dd-MS^2 acquisition.

**Compound**	**Chemical formula**	**Ion**	**m/z of precursor ion**	**Page(s)**
Loganic acid	$C_{16}H_{24}O_{10}$	$[M+NH_4]^+$	394.17062	S39
Secologanin	$C_{17}H_{24}O_{10}$	$[M+H]^+$	389.14429	S40
Mauritianin	$C_{33}H_{40}O_{19}$	$[M+H]^+$	741.22357	S41
Rutin	$C_{27}H_{30}O_{16}$	$[M+H]^+$	611.16064	S42
Peonidin rutinoside	$C_{28}H_{33}O_{15}$	$[M]^+$	609.18164	S43
Petunidin glucoside	$C_{22}H2_{3}O_{12}$	$[M]^+$	479.11776	S44
Petunidin rutinoside like	$C_{28}H_{33}O_{16}$	$[M]^+$	625.17554	S45
Hirsutidin	$C_{18}H_{17}O_{7}$	$[M]^+$	345.09656	S46
Hirsutidin rutinoside like	$C_{30}H_{37}O_{16}$	$[M]^+$	653.20673	S47
Strictosidine	$C_{27}H_{34}N_{2}O_{9}$	$[M+H]^+$	531.23383	S48
Catharanthine	$C_{21}H_{24}N_{2}O_{2}$	$[M+H]^+$	337.19064	S49
Deacetylvindoline	$C_{23}H_{30}N_{2}O_{5}$	$[M+H]^+$	415.22247	S50
Vindoline	$C_{25}H_{32}N_{2}O_{6}$	$[M+H]^+$	457.23325	S51
Vindorosine	$C_{24}H_{30}N_{2}O_{5}$	$[M+H]^+$	427.22241	S52
Tabersonine	$C_{21}H_{24}N_{2}O_{2}$	$[M+H]^+$	337.19080	S53
Hörhammericine	$C_{21}H_{24}N_{2}O_{4}$	$[M+H]^+$	369.18051	S54
Serpentine	$C_{21}H_{20}N_{2}O_{3}$	$[M]^+$	349.15448	S55
Vindolinine	$C_{21}H_{24}N_{2}O_{2}$	$[M+H]^+$	337.19077	S56
Coronaridine	$C_{21}H_{26}N_{2}O_{2}$	$[M+H]^+$	339.20657	S57-S58
Anhydrovinblastine iminium	$C_{46}H_{54}N_{4}O_{8}$	$[M+2H]^{2+}$	396.20432	S59
Anhydrovinblastine	$C_{46}H_{56}N_{4}O_{8}$	$[M+2H]^{2+}$	397.21222	S60
Vinblastine	$C_{46}H_{58}N_{4}O_{9}$	$[M+2H]^{2+}$	406.21741	S61

Loganic acid

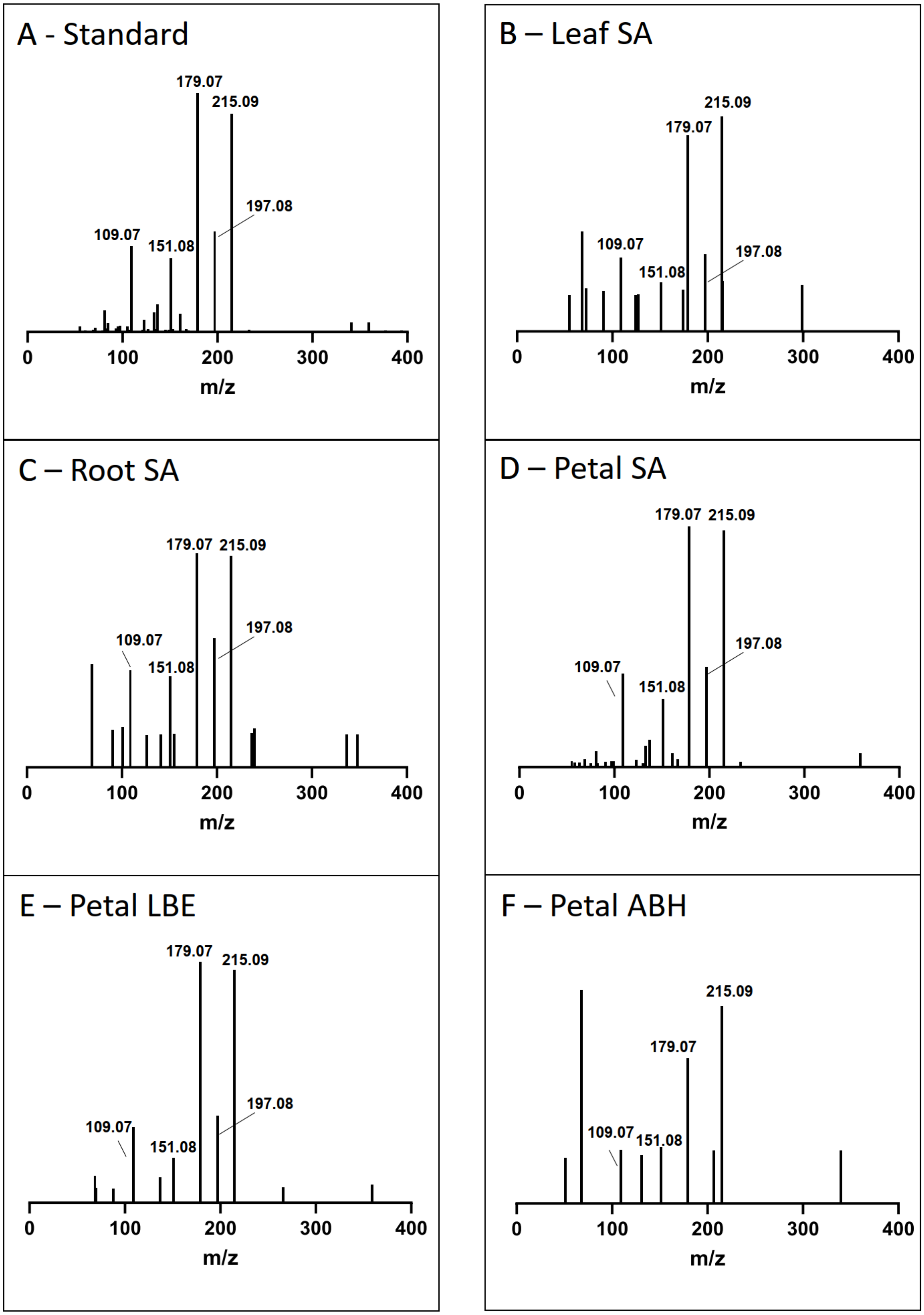

Secologanin

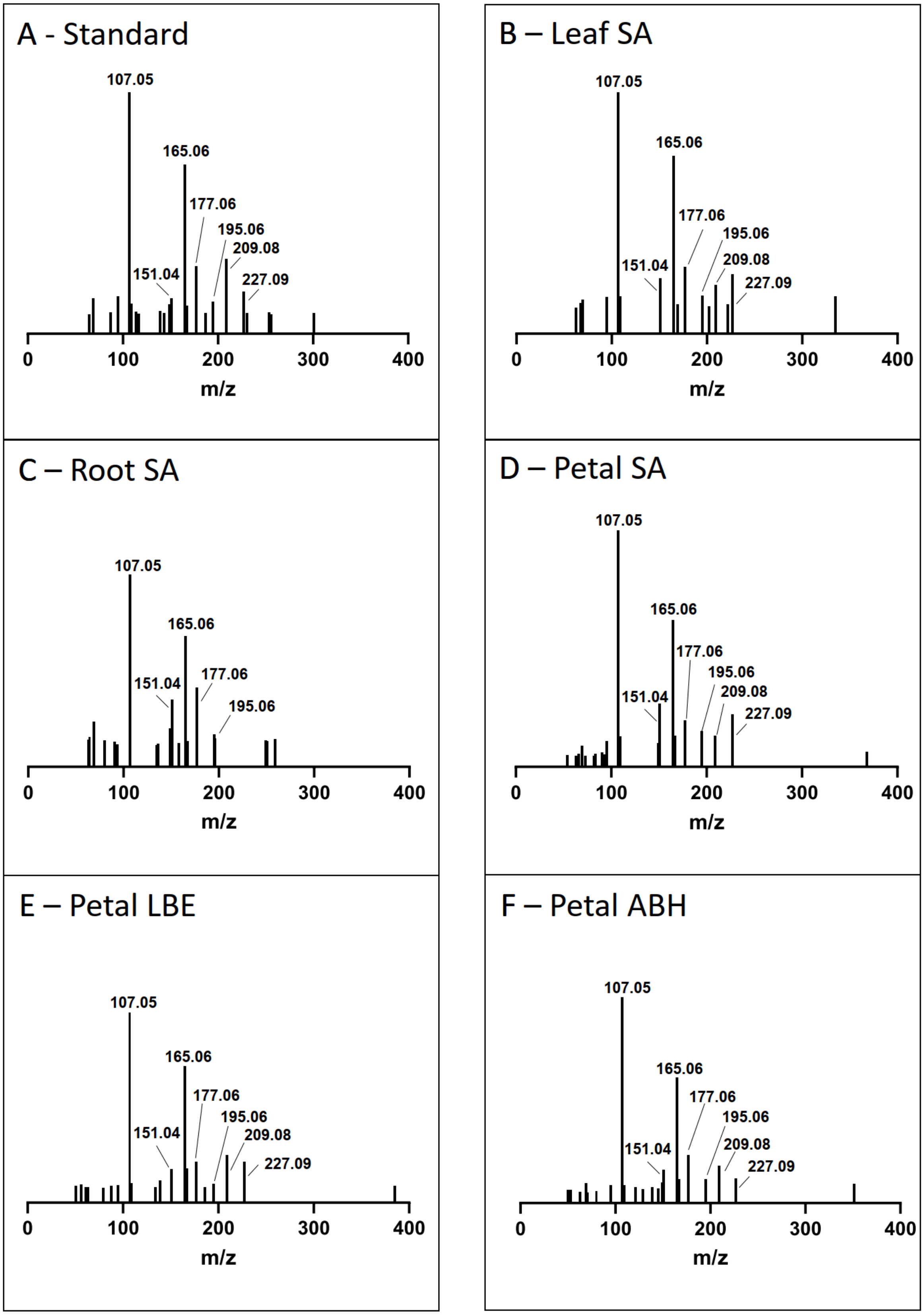

Mauritianin

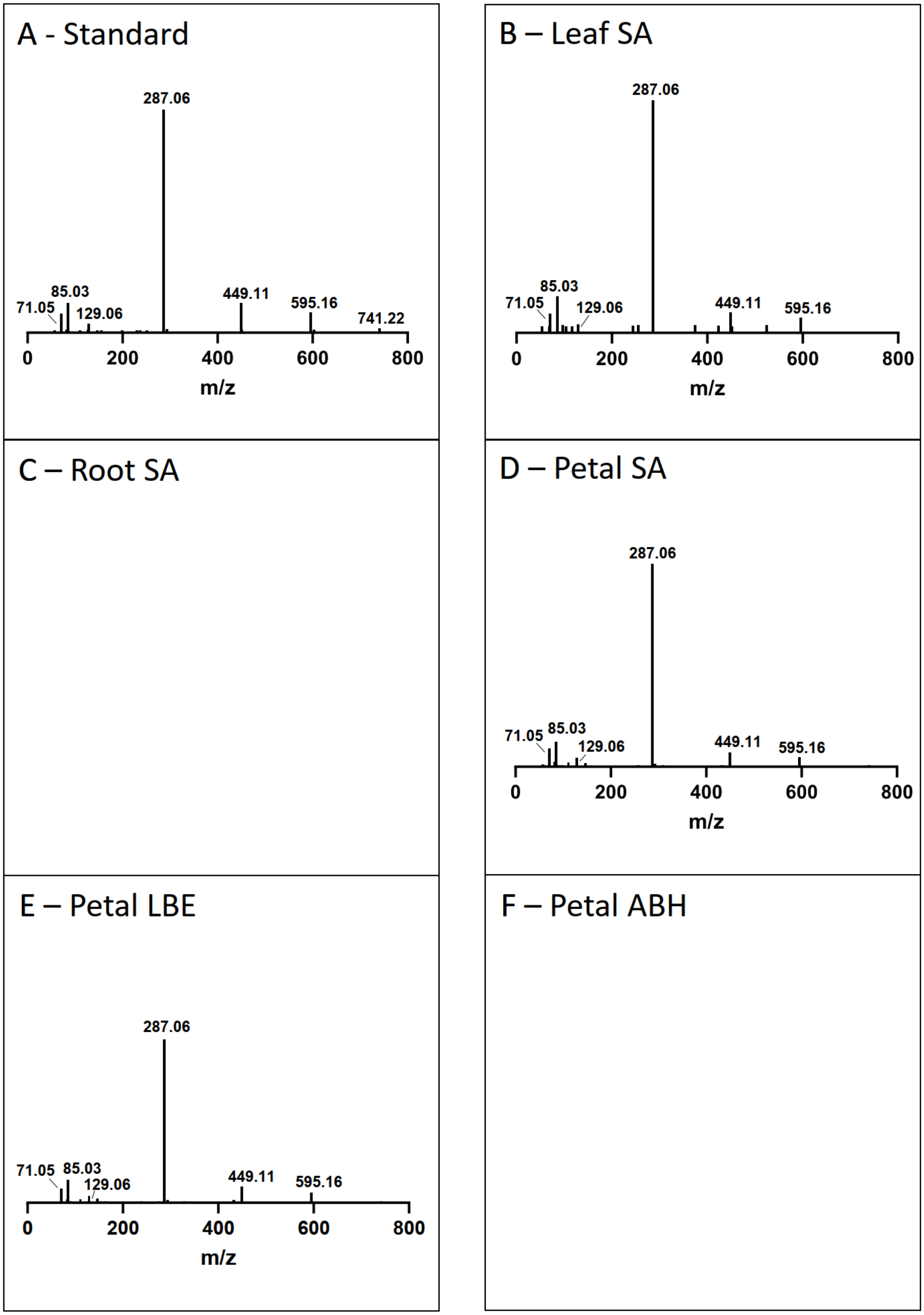

Rutin

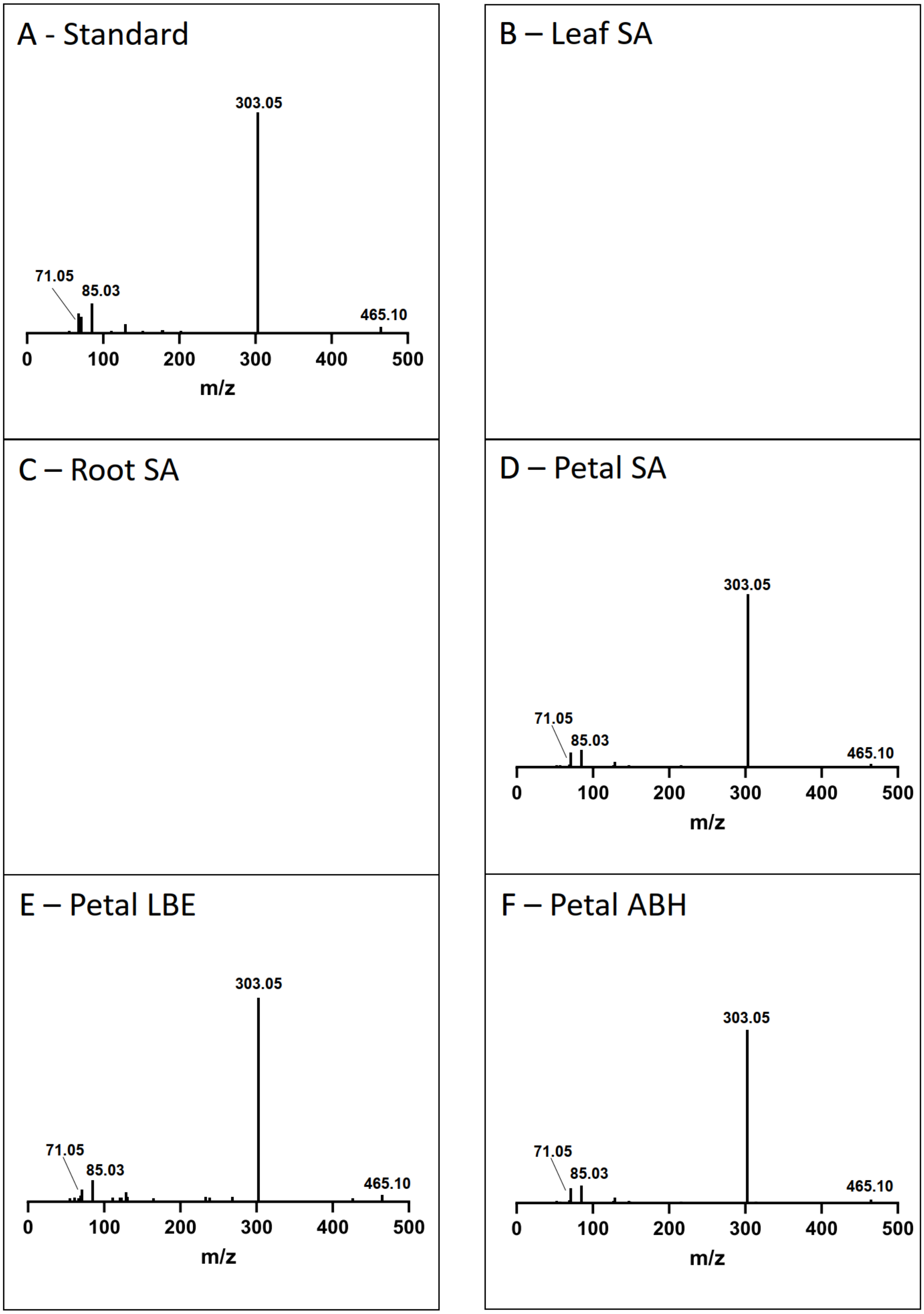
A - Standard
303.05
71.05
85.03
465.10
0
100
200
300
400
500
m/z
B – Leaf SA
C – Root SA
D – Petal SA
303.05
71.05
85.03
465.10
0
100
200
300
400
500
m/z
E – Petal LBE
303.05
71.05
85.03
465.10
0
100
200
300
400
500
m/z
F – Petal ABH
303.05
71.05
85.03
465.10
0
100
200
300
400
500
m/z

Peonidin rutinoside

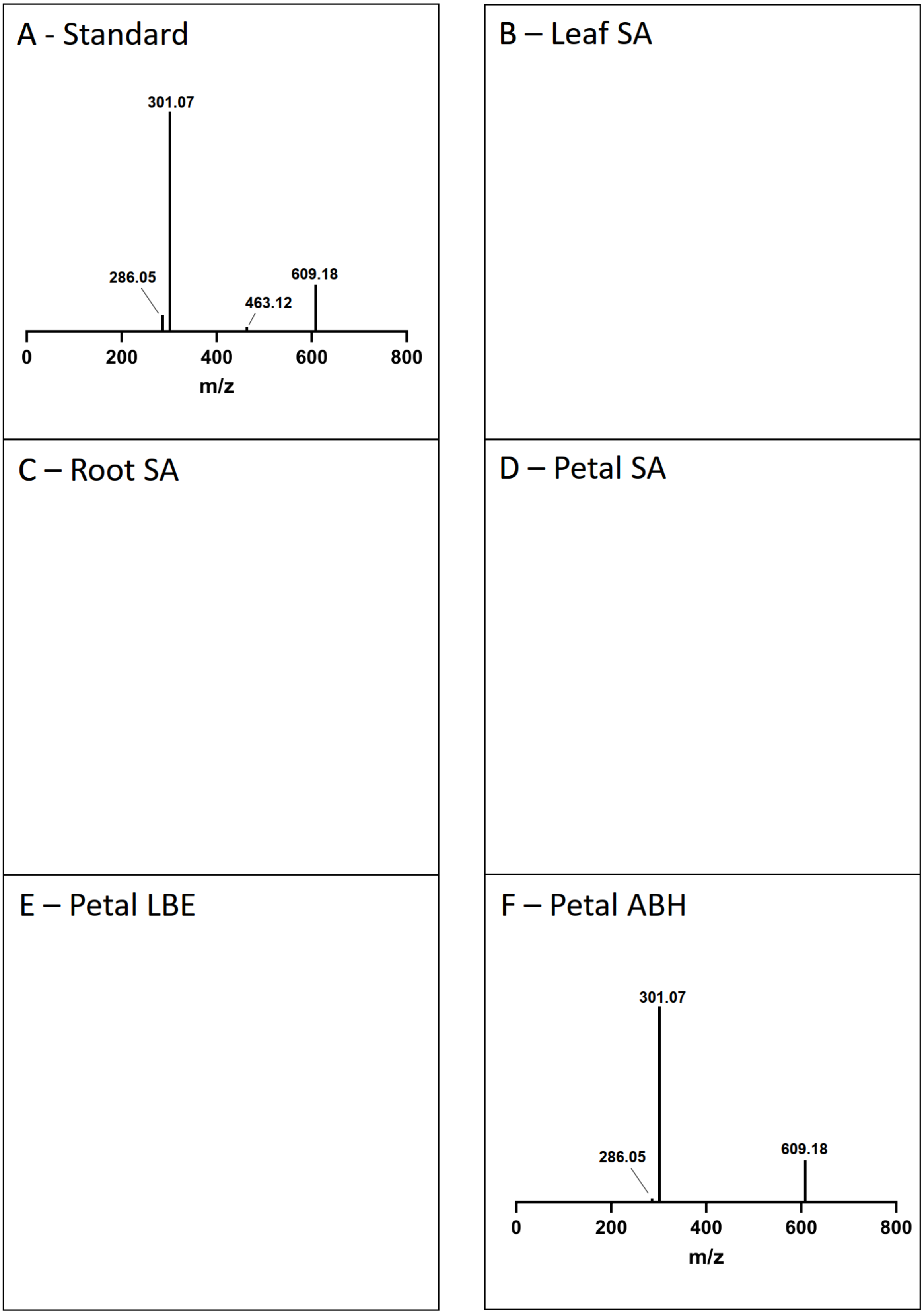

Petunidin glucoside

A - Standard

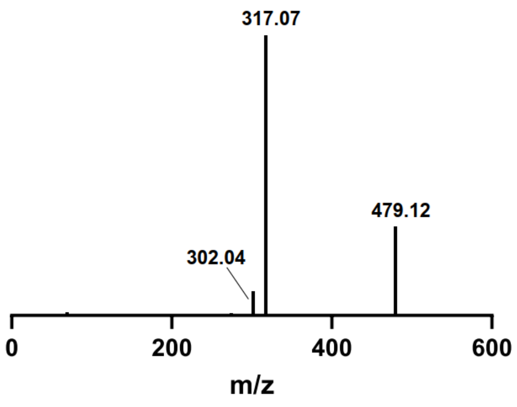

B – Leaf SA

C – Root SA

D – Petal SA

E – Petal LBE

F – Petal ABH

Petunidin rutinoside like

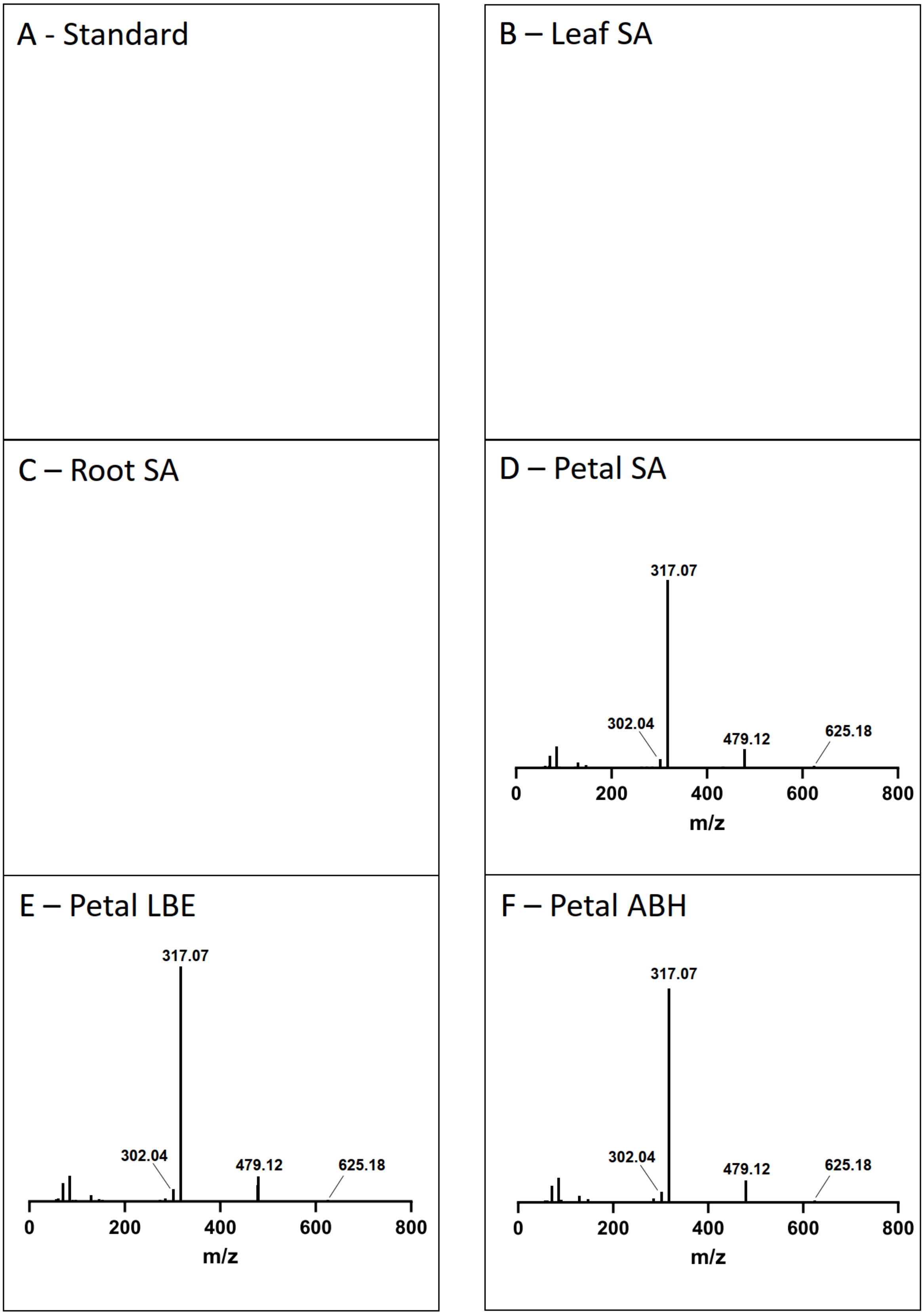

Hirsutidin

A - Standard

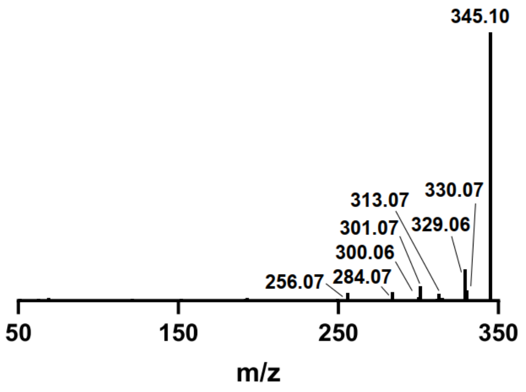

B – Leaf SA

C – Root SA

D – Petal SA

E – Petal LBE

F – Petal ABH

Hirsutidin rutinoside like

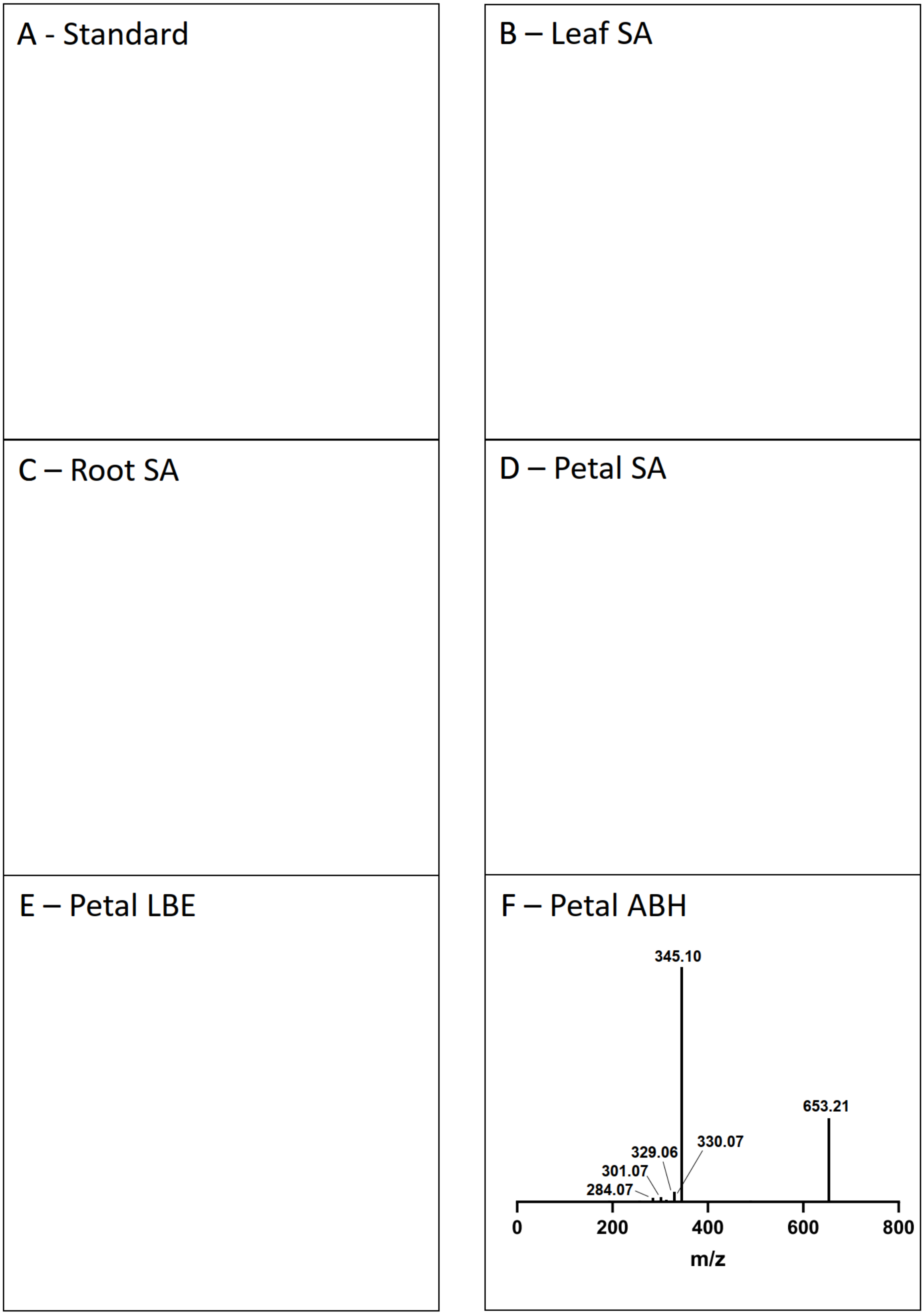

Strictosidine

A - Standard

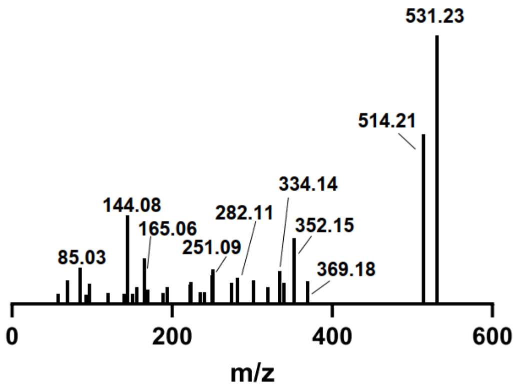

B – Leaf SA

C – Root SA

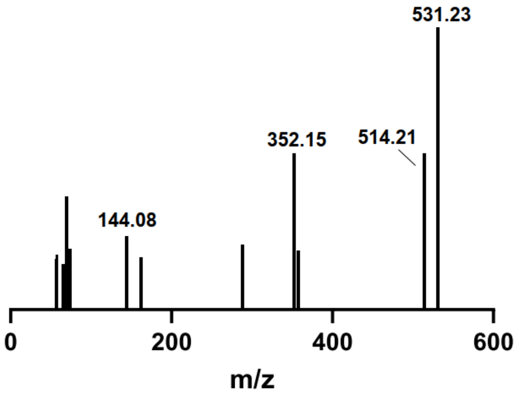

D – Petal SA

E – Petal LBE

F – Petal ABH

Catharanthine

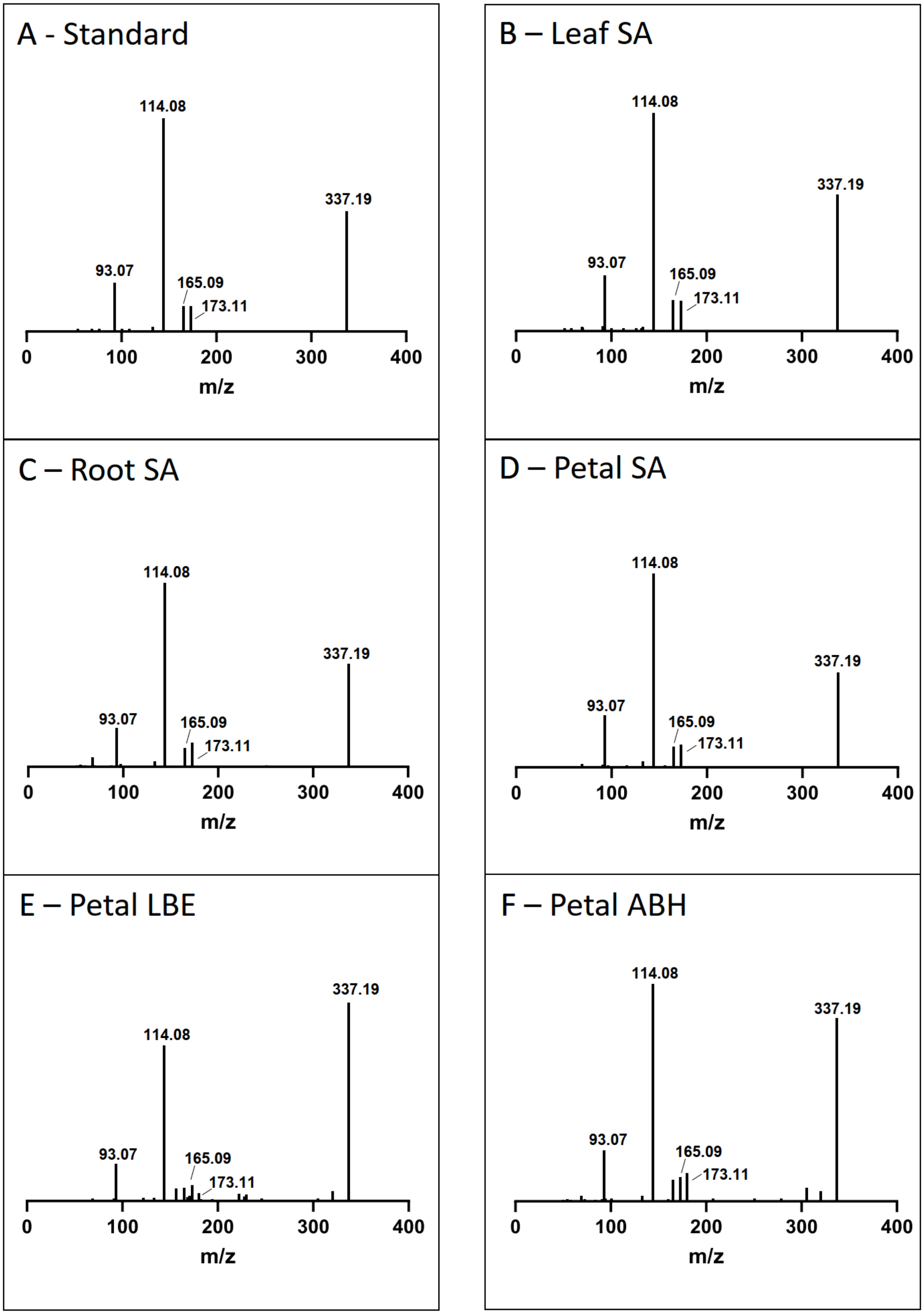

Deacetylvindoline

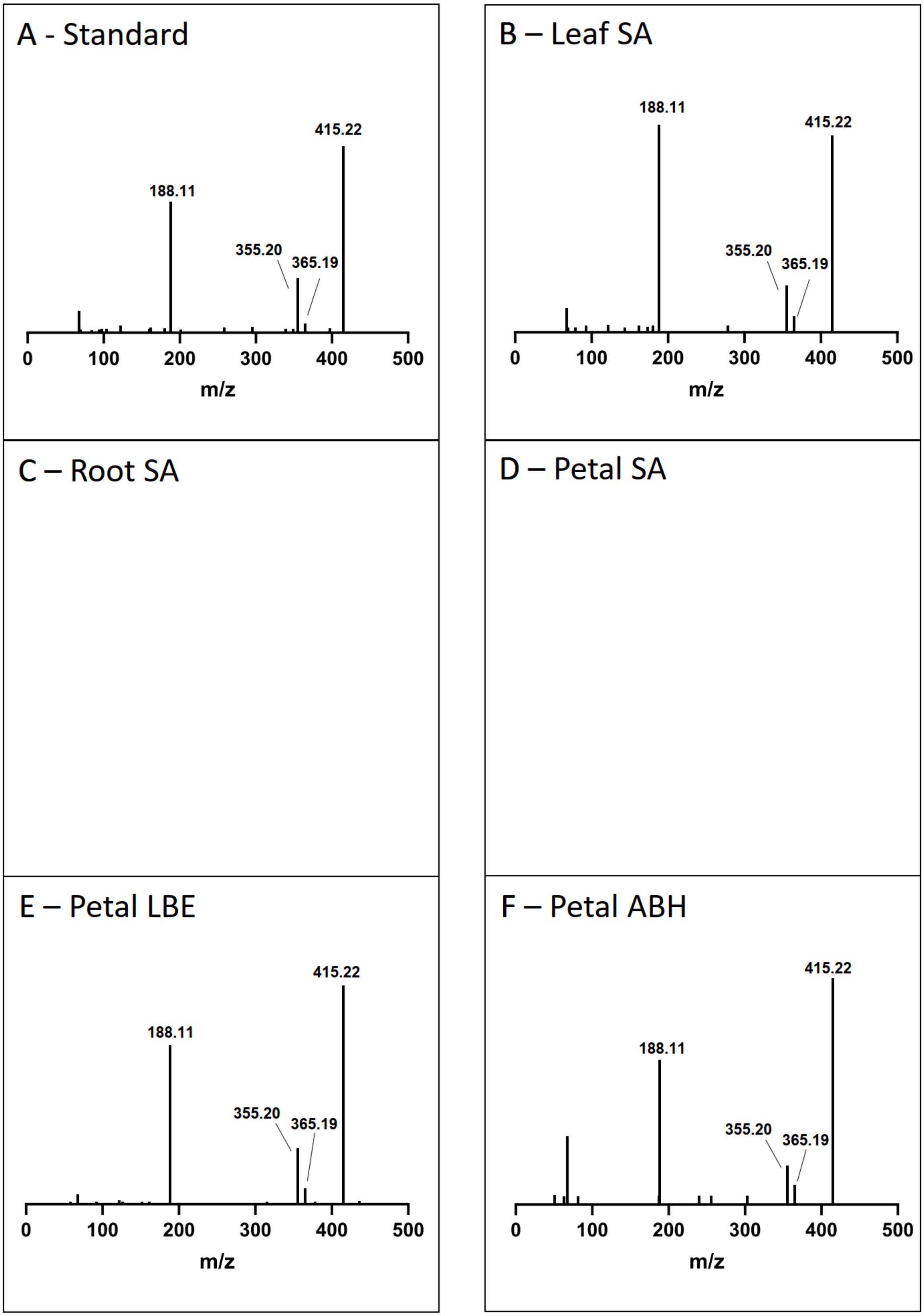

Vindoline

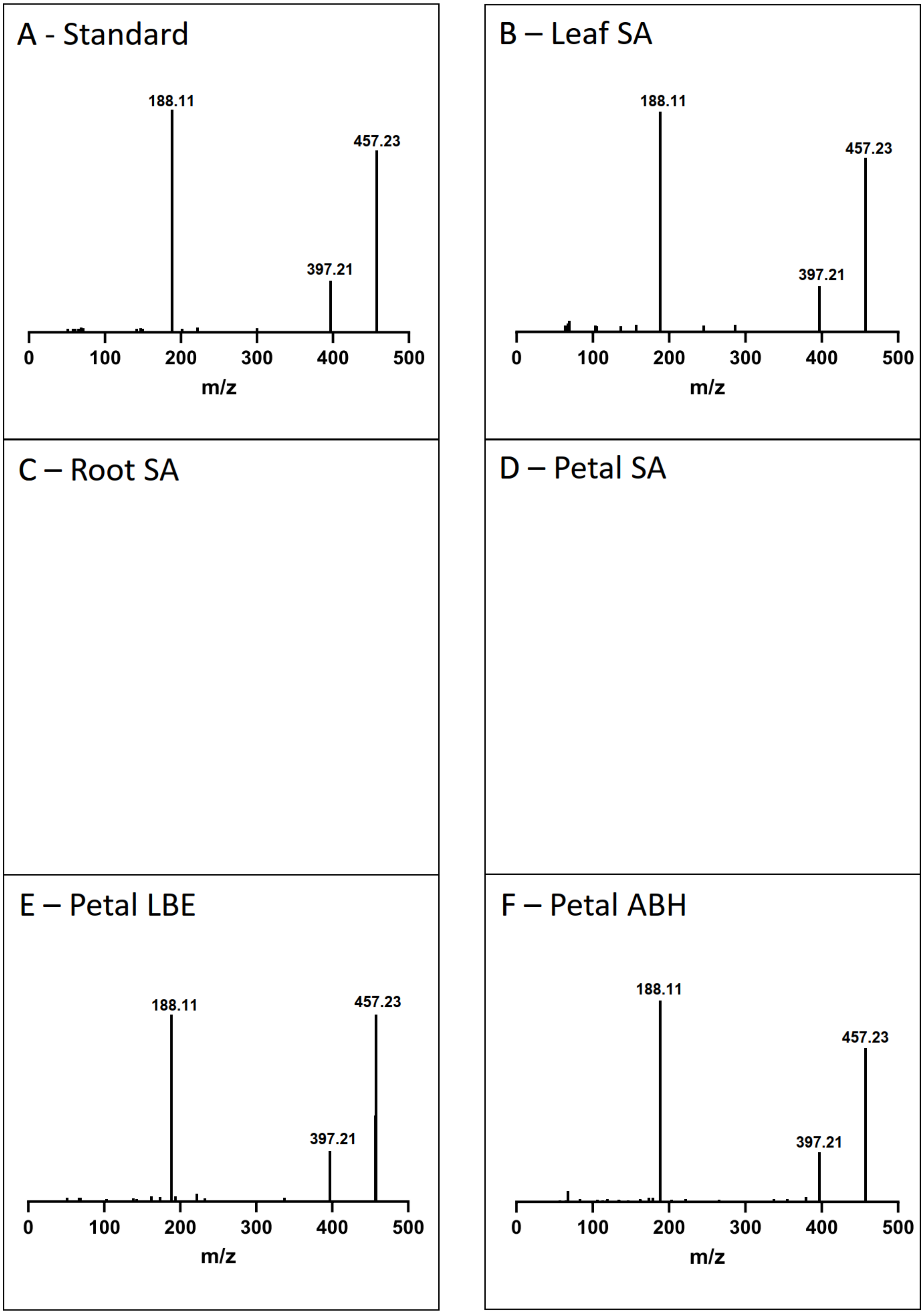
A - Standard
188.11
457.23
397.21
0
100
200
300
400
500
m/z
B – Leaf SA
188.11
457.23
397.21
0
100
200
300
400
500
m/z
C – Root SA
D – Petal SA
E – Petal LBE
188.11
457.23
397.21
0
100
200
300
400
500
m/z
F – Petal ABH
188.11
457.23
397.21
0
100
200
300
400
500
m/z

Vindorosine

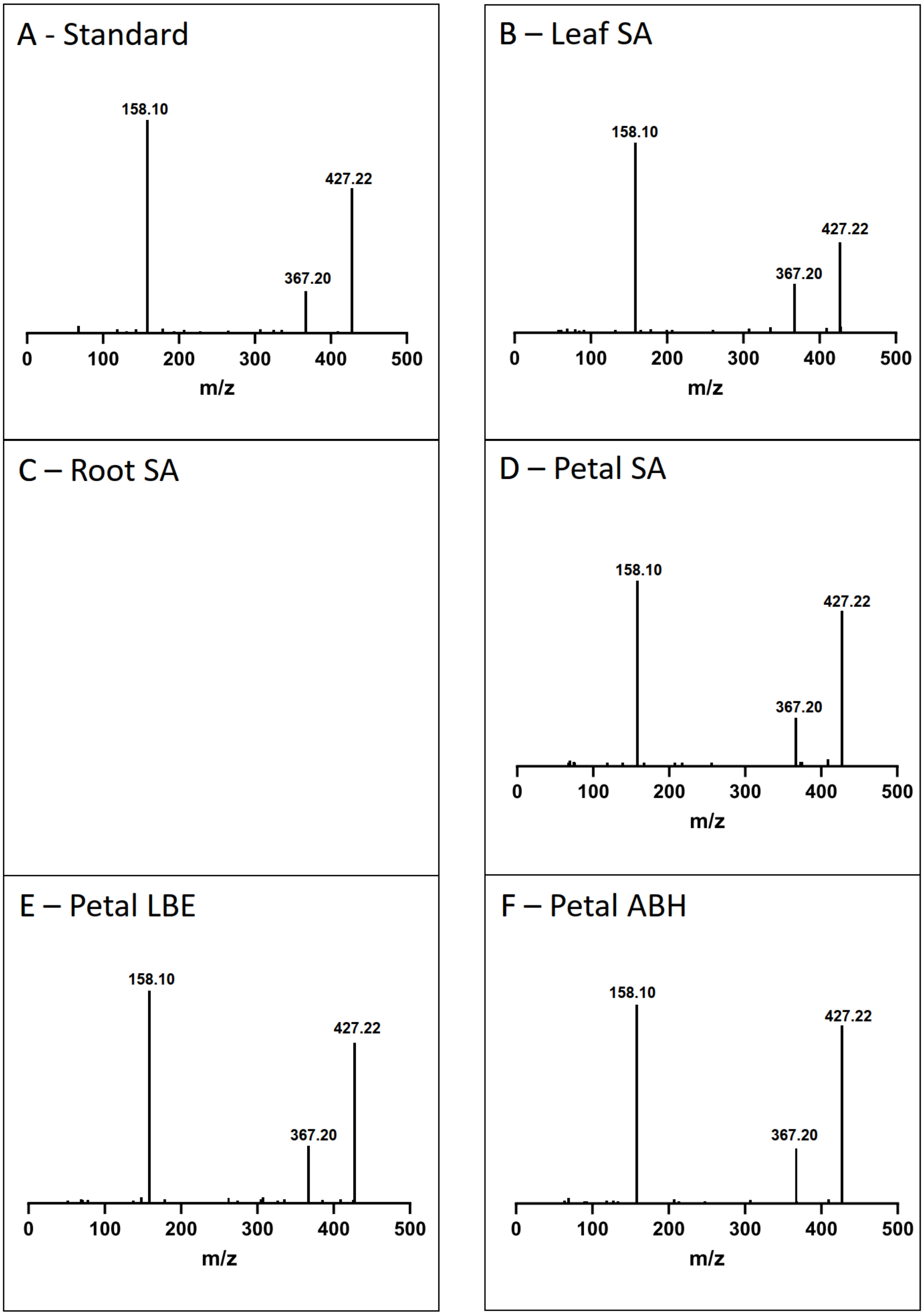

Tabersonine

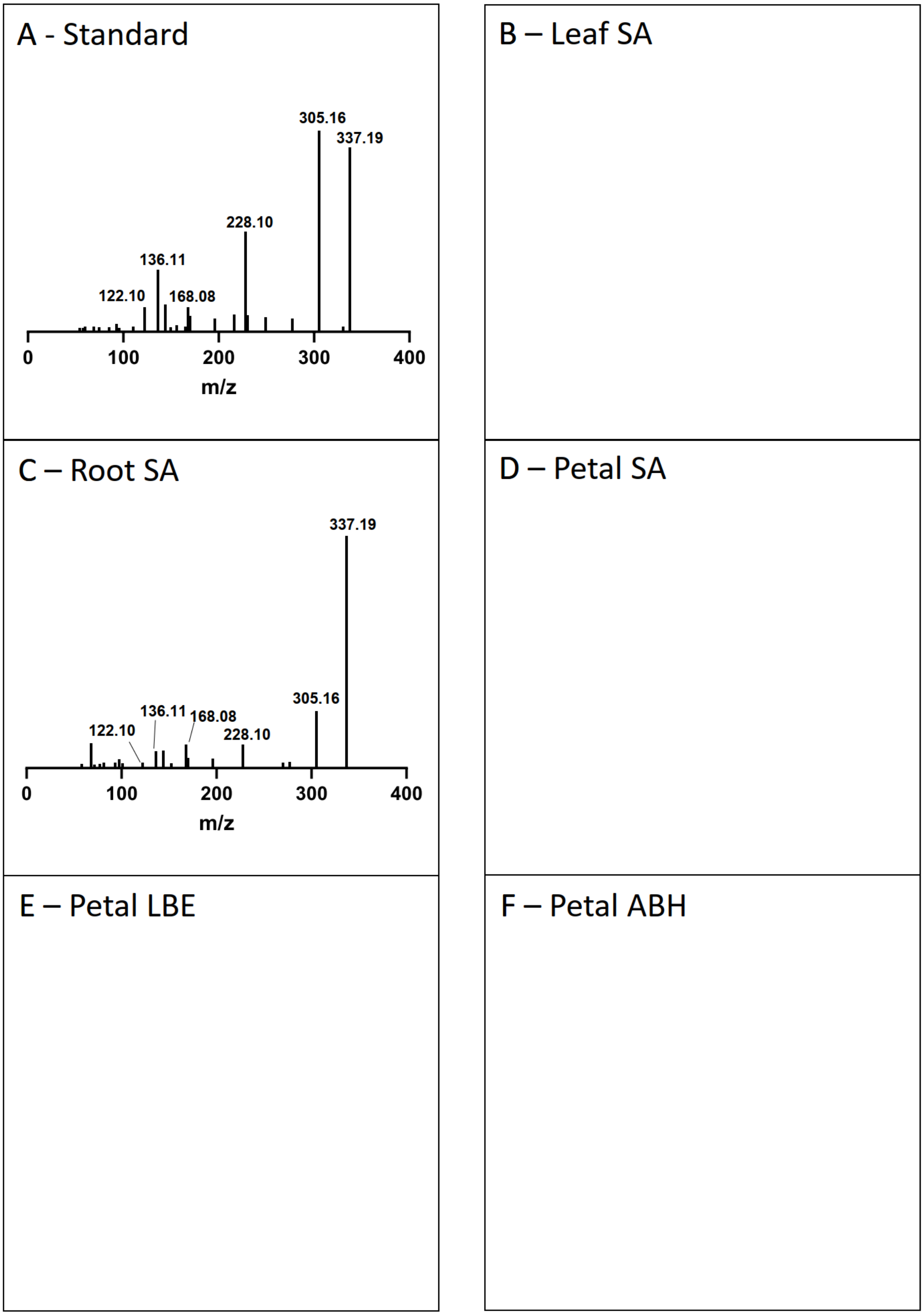

Hörhammericine

A - Standard

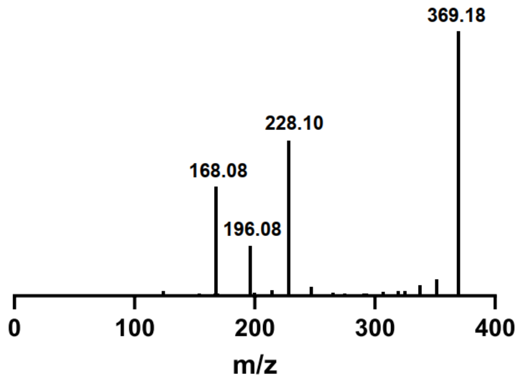

B – Leaf SA

C – Root SA

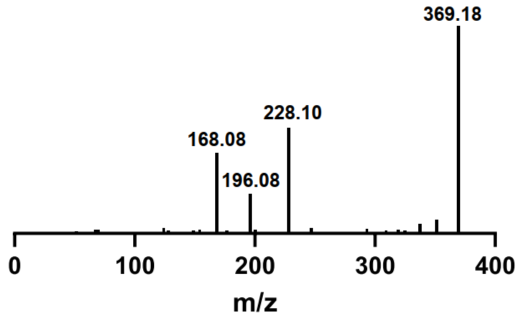

D – Petal SA

E – Petal LBE

F – Petal ABH

Serpentine

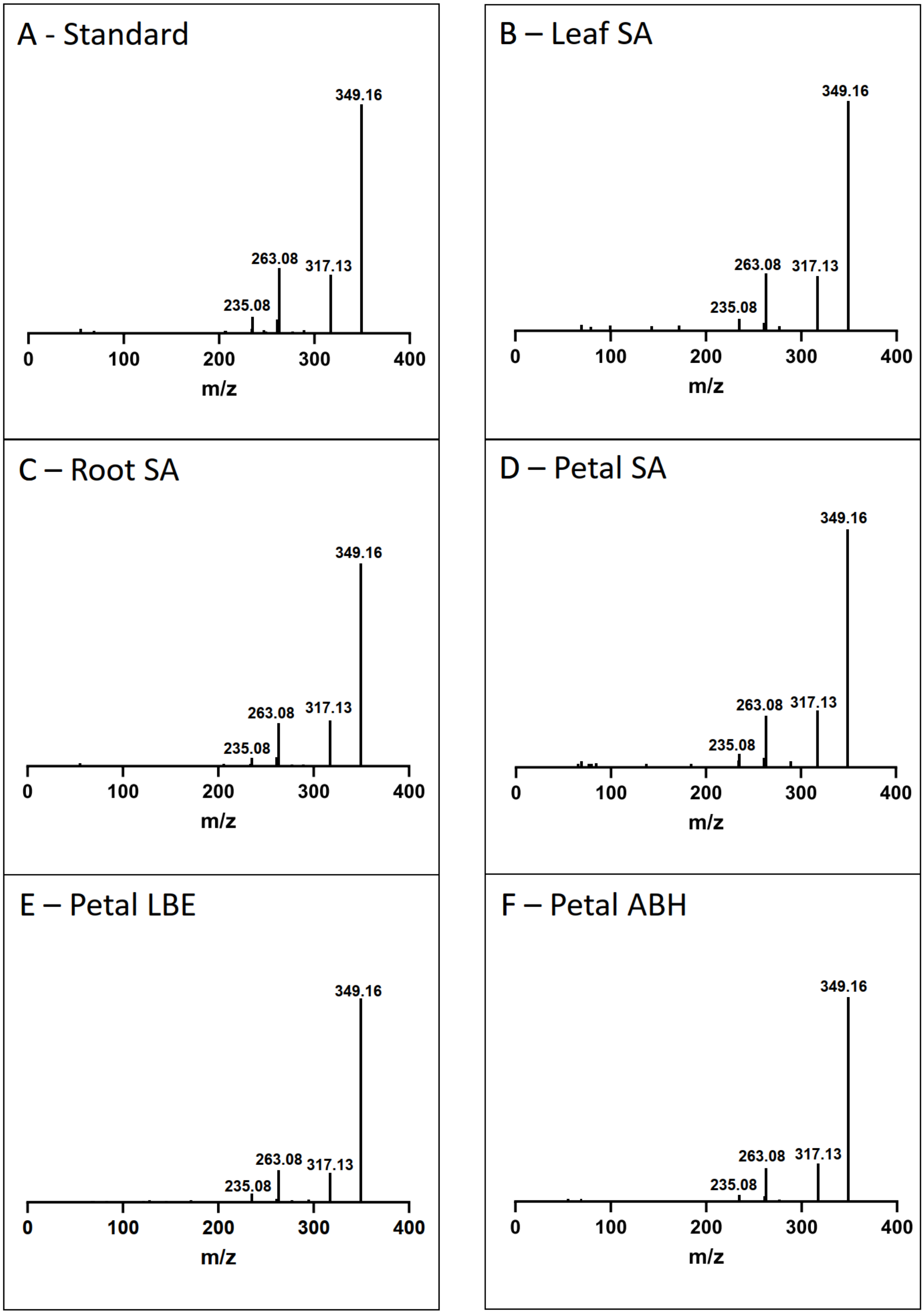
A - Standard
349.16
263.08
317.13
235.08
0
100
200
300
400
m/z
B – Leaf SA
349.16
263.08
317.13
235.08
0
100
200
300
400
m/z
C – Root SA
349.16
263.08
317.13
235.08
0
100
200
300
400
m/z
D – Petal SA
349.16
263.08
317.13
235.08
0
100
200
300
400
m/z
E – Petal LBE
349.16
263.08
317.13
235.08
0
100
200
300
400
m/z
F – Petal ABH
349.16
263.08
317.13
235.08
0
100
200
300
400
m/z

Vindolinine

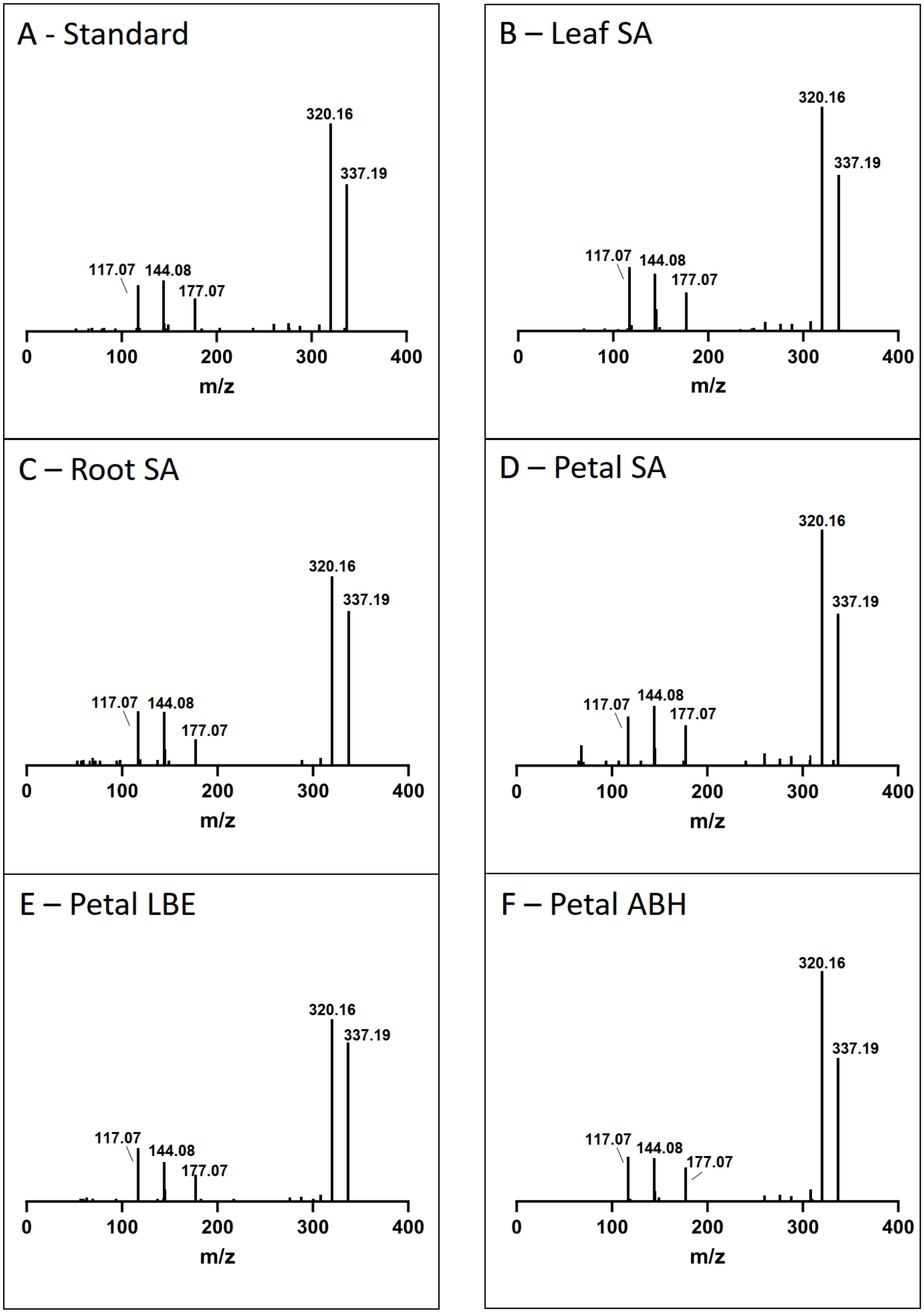

Coronaridine

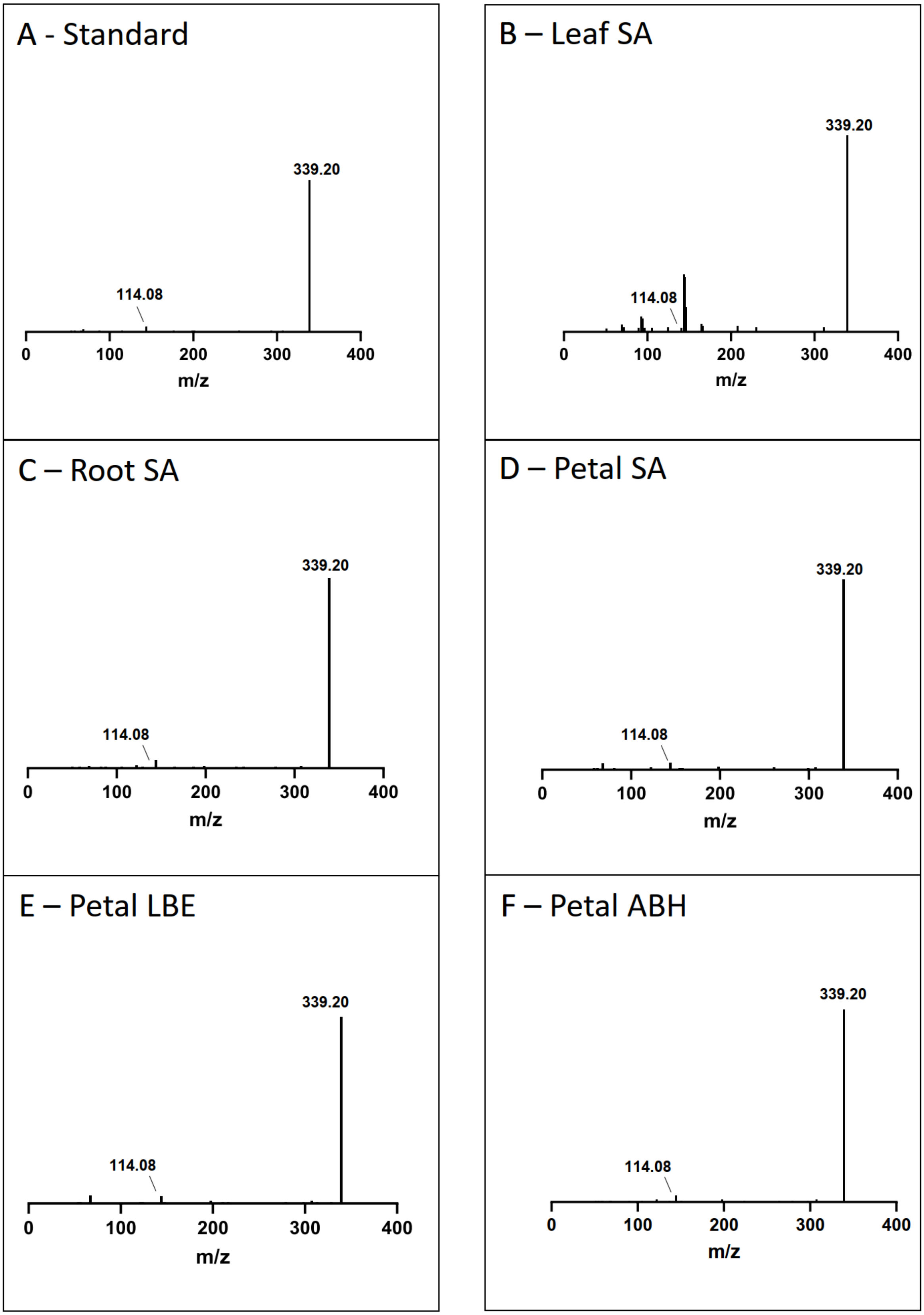

Anhydrovinblastine iminium

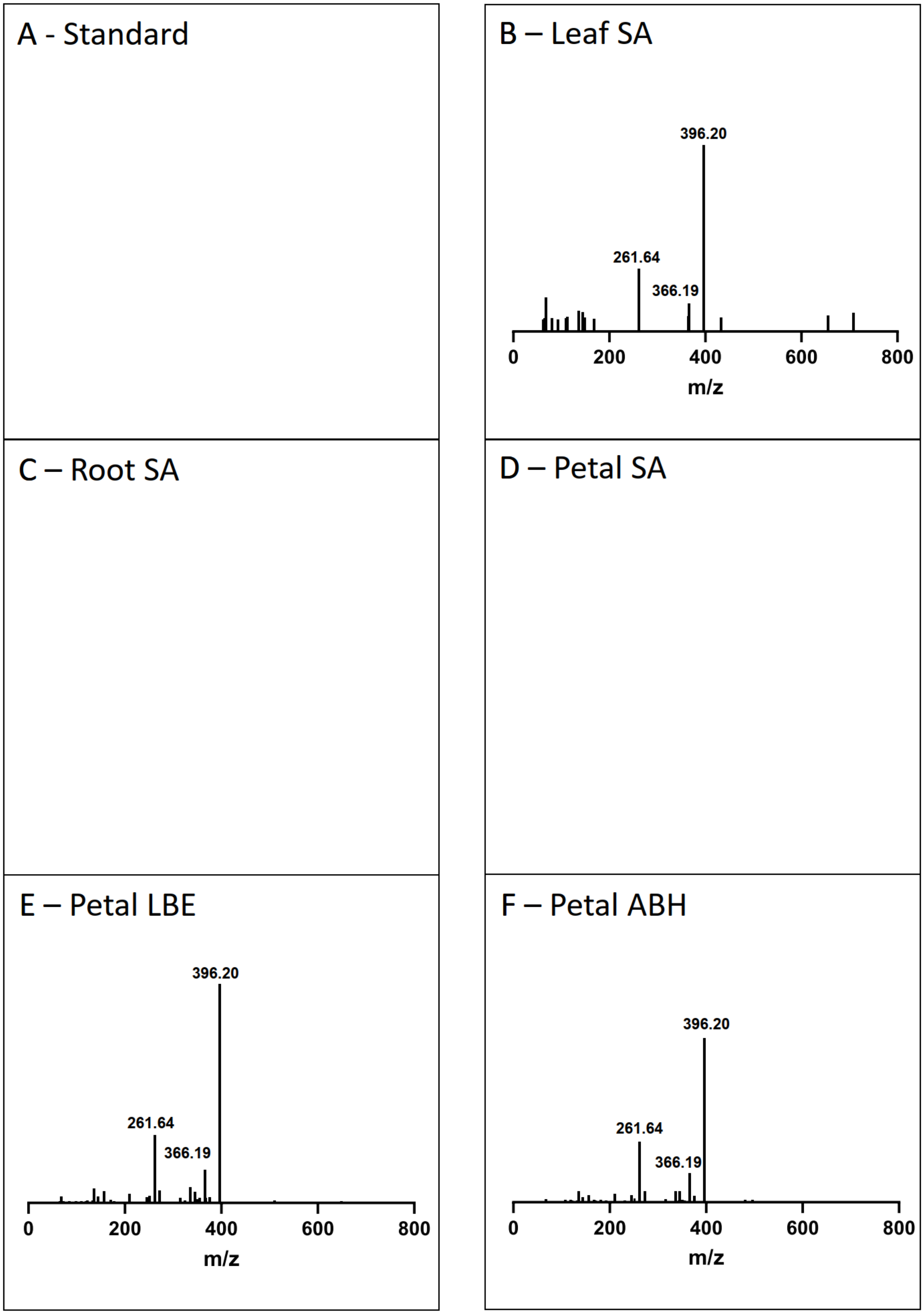

Anhydrovinblastine iminium - MS1

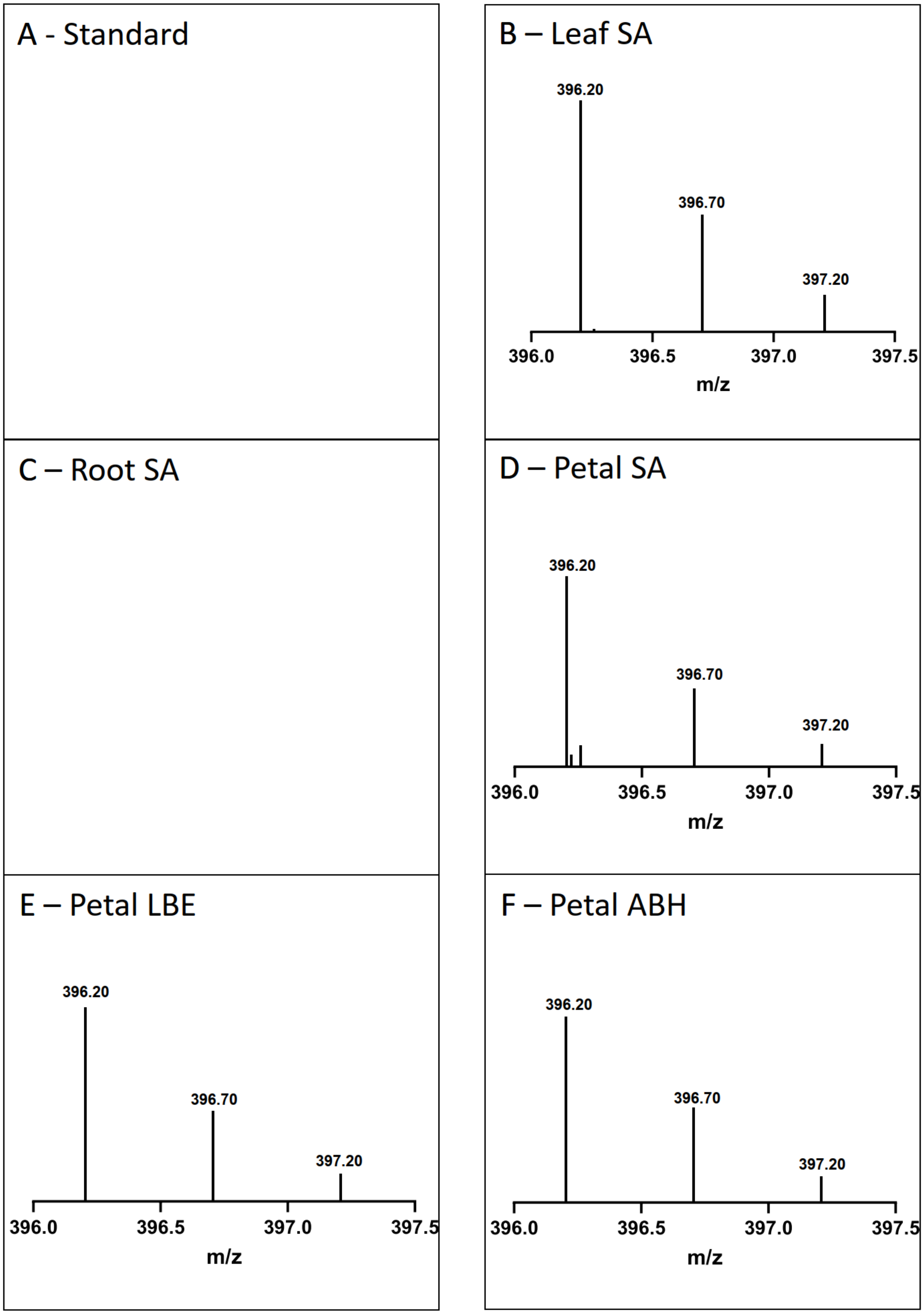

Anhydrovinblastine

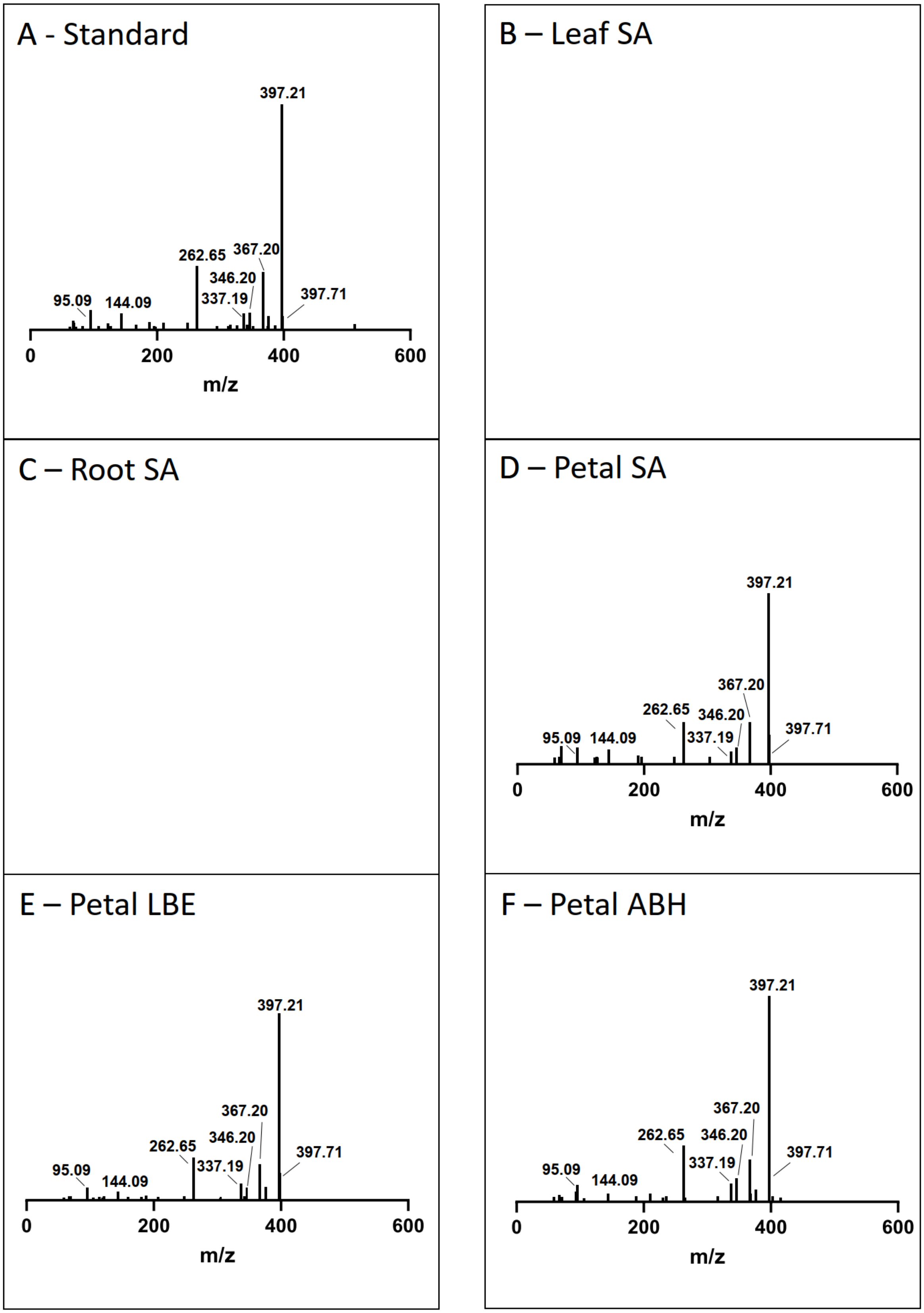

Vinblastine

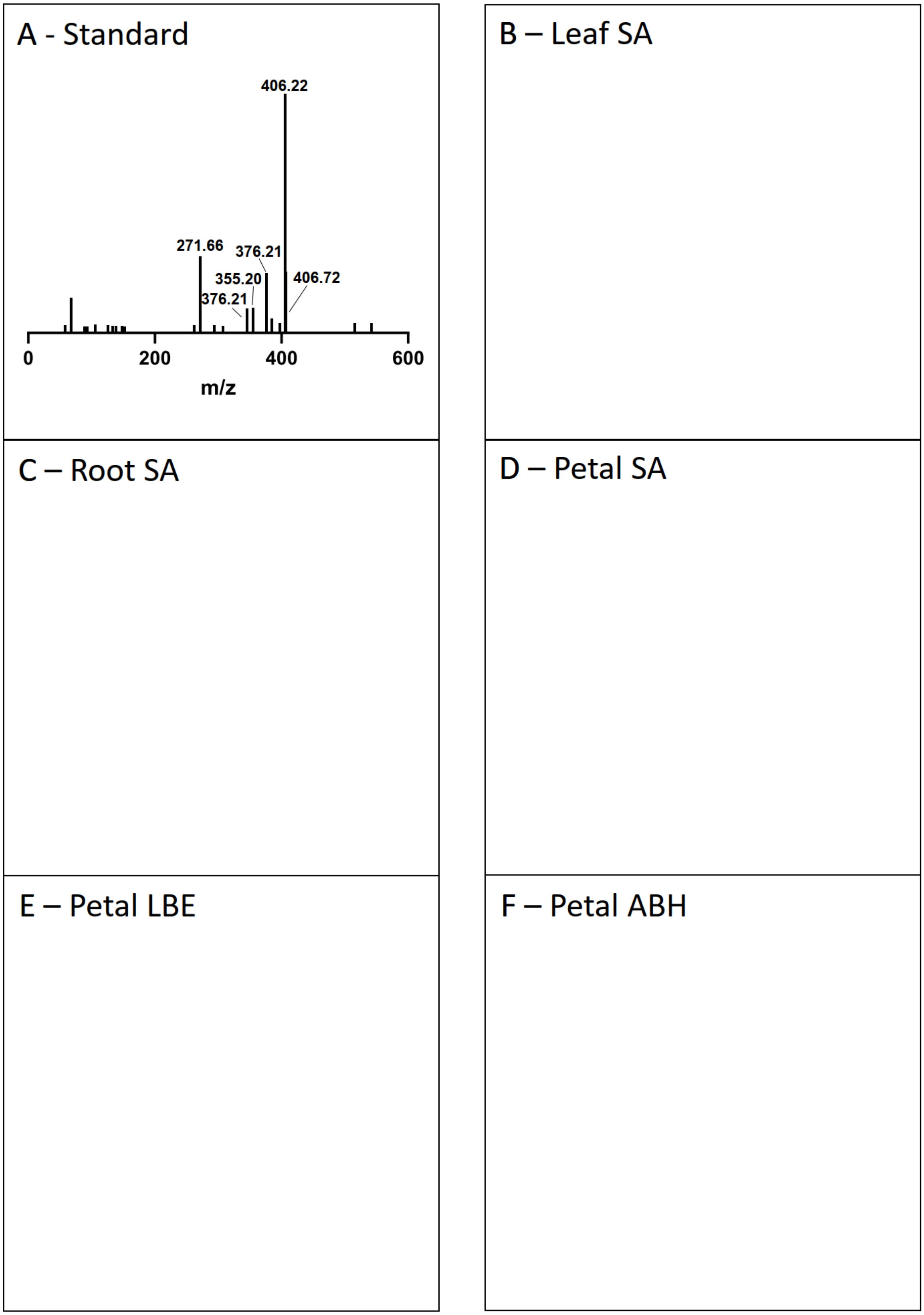

References

1. Dührkop, K. et al. SIRIUS 4: a rapid tool for turning tandem mass spectra into metabolite structure information. *Nat Methods* **16**, 299-302 (2019).
2. Dührkop, K. et al. Systematic classification of unknown metabolites using high-resolution fragmentation mass spectra. *Nat Biotechnol* **39**, 462-471 (2021).
3. Kim, H.W. et al. NPClassifier: A Deep Neural Network-Based Structural Classification Tool for Natural Products. *J Nat Prod* **84**, 2795-2807 (2021).
4. Yuan, C. & Yang, H. Research on K-Value Selection Method of K-Means Clustering Algorithm. *J* **2**, 226-235 (2019).
5. Gu, Z. & Hübschmann, D. Make Interactive Complex Heatmaps in R. *Bioinformatics* **38**, 1460-1462 (2021).
6. Li, C. et al. Single-cell multi-omics in the medicinal plant Catharanthus roseus. *Nature Chemical Biology* **19**, 1031-1041 (2023).
7. Clark, I.C. et al. Microfluidics-free single-cell genomics with templated emulsification. *Nat Biotechnol* **41**, 1557-1566 (2023).
8. Kaminow, B., Yunusov, D. & Dobin, A. STARsolo: accurate, fast and versatile mapping/quantification of single-cell and single-nucleus RNA-seq data. *bioRxiv*, 2021.2005.2005.442755 (2021).
9. Yang, S. et al. Decontamination of ambient RNA in single-cell RNA-seq with DecontX. *Genome Biology* **21**, 57 (2020).
10. Stuart, T. et al. Comprehensive Integration of Single-Cell Data. *Cell* **177**, 1888-1902.e1821 (2019).
11. Kim, J.-Y. et al. Distinct identities of leaf phloem cells revealed by single cell transcriptomics. *The Plant Cell* **33**, 511-530 (2021).
